# Single-nucleus multiple-organ chromatin accessibility landscape in the adult rat

**DOI:** 10.1093/gigascience/giag013

**Published:** 2026-02-03

**Authors:** Ronghai Li, Shanshan Duan, Qiuting Deng, Wen Ma, Chang Liu, Peng Gao, Li Lu, Yue Yuan

**Affiliations:** State Key Laboratory of Genome and Multiomics Technologies, BGI Research, Shenzhen 518083, China; State Key Laboratory of Genome and Multiomics Technologies, BGI Research, Hangzhou 310030, China; College of Life Sciences, University of Chinese Academy of Sciences, Beijing 100049, China; State Key Laboratory of Genome and Multiomics Technologies, BGI Research, Shenzhen 518083, China; State Key Laboratory of Genome and Multiomics Technologies, BGI Research, Shenzhen 518083, China; State Key Laboratory of Genome and Multiomics Technologies, BGI Research, Shenzhen 518083, China; Shanxi Medical University - BGI Collaborative Center for Future Medicine, Shanxi Medical University, Taiyuan 030001, China; Shenzhen Proof-of-Concept Center of Digital Cytopathology, BGI Research, Shenzhen 518083, China; Shanxi Medical University - BGI Collaborative Center for Future Medicine, Shanxi Medical University, Taiyuan 030001, China; BGI, Shenzhen 518083, China; Shanxi Medical University - BGI Collaborative Center for Future Medicine, Shanxi Medical University, Taiyuan 030001, China; School of Basic Medical Sciences, Shanxi Medical University, Taiyuan 030001, China; Key Laboratory of Cellular Physiology of Chinese Ministry of Education, Shanxi Medical University, Taiyuan 030001, China; State Key Laboratory of Genome and Multiomics Technologies, BGI Research, Hangzhou 310030, China

**Keywords:** single-nucleus ATAC-seq, rat cell atlas, single-cell chromatin accessibility, epigenomics, transcription factor, single-cell analysis, cellular classification, cross-organ analysis, cross-species analysis

## Abstract

**Background:**

Chromatin accessibility landscape is the basis of cell-specific gene expression and reflects the regulatory logic underlying cellular identity and function. However, a systematic multi-organ chromatin accessibility atlas in the rat (*Rattus norvegicus*), an important biomedical model organism, has been lacking.

**Results:**

We generated a multi-organ single-nucleus chromatin accessibility landscape of *Rattus norvegicus* using snATAC-seq. For this single-cell atlas, we constructed 25 libraries via snATAC-seq from 9 organs in the adult rat, with a total of over 110,000 cells. Cell classification integrating gene activity scores with known marker genes identified 77 cell types, which were strongly correlated with those in published mouse single-cell transcriptome atlases. We further investigated the enrichment of cell-type- and organ-specific transcription factors, shared and organ-specific features of endothelial and stromal cells, as well as cross-organ macrophage regulatory states, and the conservation and specificity of gene regulatory programs across species.

**Conclusion:**

This single-nucleus chromatin accessibility landscape provides a valuable foundation for dissecting tissue-specific regulatory mechanisms in the rat and facilitates cross-organ and cross-species cell type annotation and functional inference, supporting broader applications of rat model in systems biology and biomedical research.

## Introduction

The Human Cell Atlas project aims to create a comprehensive reference cell atlas of all cells in the human body (the basic unit of life). This will serve as a basis for understanding human health and for diagnosing, monitoring, and treating disease. However, a key scientific question is what insights can be gained from cell atlases. To date, studies of cell atlases have advanced our understanding of anatomy, development, physiology, pathology, and intra- and intercellular regulation at a new level of granularity. They have also advanced our understanding of cellular diversity, revealing the cellular compositions of complex tissues and organs and how cells interact with each other in states of health and disease [1]. The development of single-cell and spatial genomics technologies, as well as the corresponding algorithms, has enabled the mapping of cells across omics, organs, species, developmental states, and diseases with unprecedented resolution. This has facilitated the systematic probing of biological questions related to cell type, spatial location, developmental trajectory, fate determination, the tumor microenvironment, and molecular mechanisms, among others. These advances provide powerful new tools and will open new avenues for clinical medicine, especially in precision medicine and personalized treatment.

Multicellular organisms are composed of specialized tissues that perform distinct physiological functions. Although different tissues and cell types generally share identical or nearly identical genomic DNA sequences, their functional diversity arises primarily from systematic differences in gene expression programs rather than changes in DNA sequence. These stable yet plastic expression states are maintained by epigenetic mechanisms, including chromatin accessibility, histone modifications, and 3D genome organization, which collectively constrain the transcriptional potential and functional identity of individual cell types [2]. Historically, studies of gene expression and epigenetic regulation have relied largely on bulk sequencing approaches that measure population-averaged signals from large numbers of cells. While such methods have been instrumental in defining tissue-level molecular features, they obscure true cell-to-cell heterogeneity and are inherently limited in their ability to resolve rare cell populations or transient regulatory states. The advent and rapid development of single-cell technologies have enabled high-resolution characterization of individual cells at a large scale, providing deeper insight into the biological mechanisms underlying tissue organization and function. Single-cell RNA sequencing (scRNA-seq) captures transcriptional profiles that inform cellular phenotypes [3], whereas single-cell assay for transposase-accessible chromatin by sequencing (scATAC-seq) directly maps the openness of regulatory elements and the potential binding of transcription factors (TFs), thereby pinpointing genomic regions involved in gene regulation [4]. This regulatory-centric view is particularly critical for elucidating the mechanisms governing cell fate specification and differentiation, as well as for identifying the upstream drivers of cellular state transitions in response to physiological stimuli, perturbations, or disease.

The laboratory rat (*Rattus norvegicus*) has been used for >150 years in biomedical research and remains a preferred model in many areas, including physiology, behavior, and the study of complex human diseases [5]. Extensive evidence indicates that rats exhibit a high degree of physiological concordance with humans in metabolic regulation, cardiovascular function, neurobehavioral processes, and endocrine systems. Their larger body size further enables precise physiological measurements, longitudinal sampling, and sophisticated surgical manipulations [6]. Despite these advantages, comparable cross-organ single-cell epigenomic resources in the rat remain notably lacking. This absence has created a critical gap between the widespread application of rat disease models and the ability to interpret molecular and cellular mechanisms at single-cell resolution. In recent years, cross-organ single-cell atlas studies have substantially advanced our understanding of tissue complexity. Large-scale single-cell transcriptomic and epigenomic efforts in humans and mice, such as Tabula Sapiens [7, 8], the Mouse Cell Atlas [9, 10], and HuBMAP [11], have systematically revealed both shared and organ-specific regulatory features across cell types, demonstrating that the same cell type can adopt markedly distinct molecular states in different tissue microenvironments. However, existing single-cell studies in the rat have largely focused on individual organs (e.g., the brain [12, 13], kidney [14], and testes [15]), leaving the cross-organ organization of cell states and regulatory programs largely unexplored. Rather than aiming to replace or compete with mouse atlases, our objective is to establish a multi-organ single-nucleus chromatin accessibility atlas in the adult rat, providing a complementary resource to dissect tissue-specific regulatory programs in a translationally relevant model organism and to serve as a foundational resource for constructing gene regulatory network models and enabling integrative, model-based analyses of large-scale single-cell data.

In our previous work, we constructed a single-nucleus chromatin accessibility landscape of the rat brain and spinal cord, providing valuable resources for understanding region- and cell type-specific gene regulation in the mammalian nervous system [16, 17]. Building upon this foundation, this study extends our efforts to profile chromatin accessibility across 9 major organs in the rat. This multi-organ dataset not only supplements and extends our previous dataset but also provides a valuable resource for the research community, supporting studies of epigenomic diversity and gene regulation across different organs and cell types in the rat.

## Results

### Single-nucleus multiple-organ chromatin accessibility landscape in the adult rat

**Figure 1:**
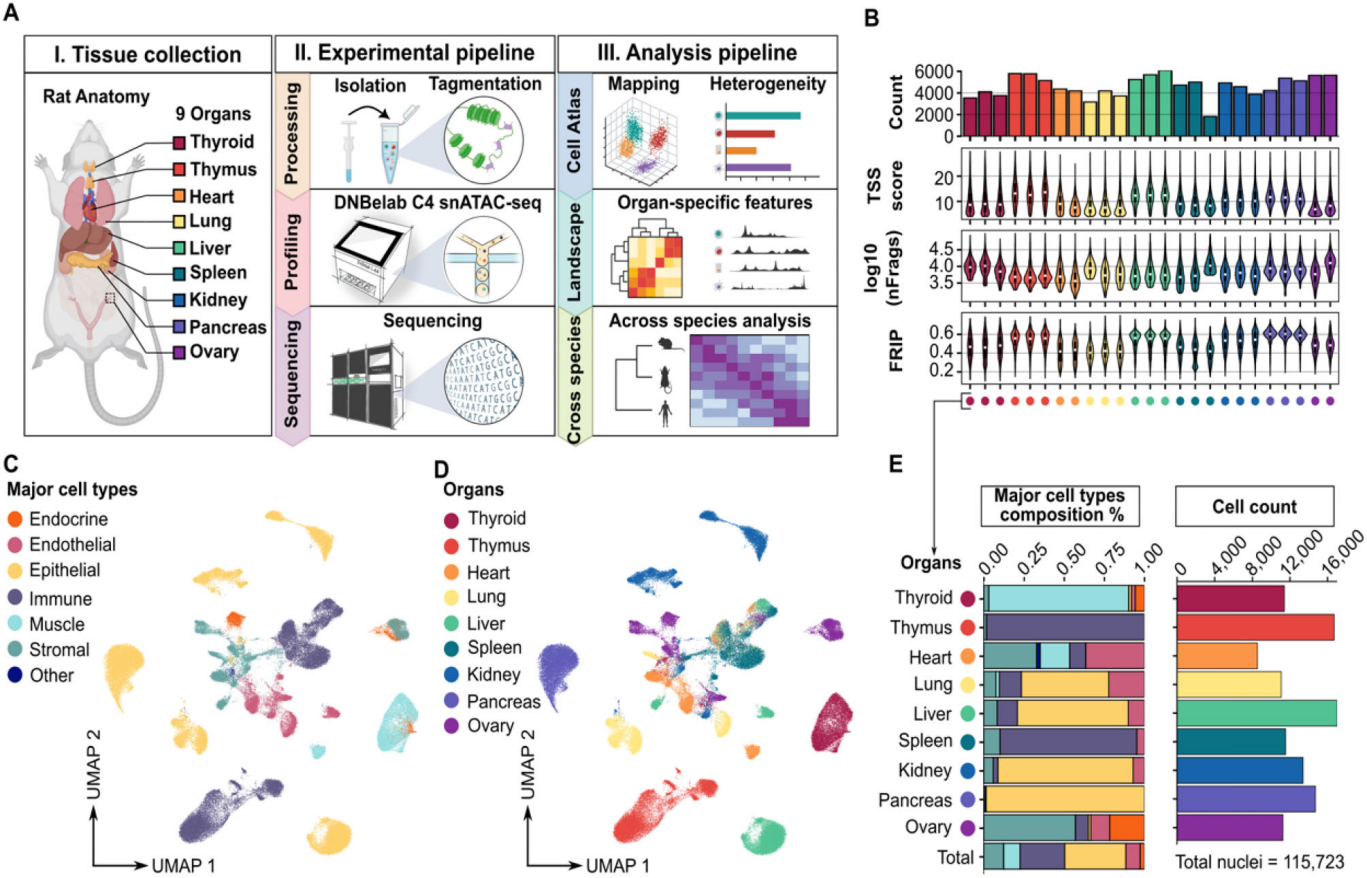
Cross-organ single-nucleus ATAC sequencing atlas of an adult rat. (A) Schematic of the study design, including the tissue collection, experimental pipeline, and analysis pipeline steps (created with BioRender.com). (B) Quality control metrics across the library, with the same color used to represent technical replicates of the same organ. The bar plot shows the number of nuclei in each library. Violin plots show the transcription start site (TSS) enrichment scores, log10 unique nuclear fragment counts, and fraction of reads in peaks for each library. (C) Cross-organ snATAC-seq atlas. The UMAP plot shows all the nuclei identified in this study, colored according to major cell type. (D) Same as (C) but colored according to organ. (E) Stacked bar plot showing the fraction of major cell types in each organ, with the total proportions of major cell types in this study shown in the bottom column (left). The bar plot shows the number of nuclei for each organ, and the total number of nuclei in this study is shown at the bottom (right).

Here, we report the cellular composition and chromatin accessibility landscape of multiple organs in the rat. The dataset consists of single-cell epigenomic data from 115,723 nuclei isolated from 9 organs (namely, the thyroid, thymus, heart, lung, liver, spleen, kidney, pancreas, and ovary) of a single female Sprague‒Dawley (SD) rat aged 7–8 months (Fig. 1A). The organs were dissociated into single-nuclear suspensions in accordance with pre-established methods and then subjected to snATAC-seq via the standard MGI DNBelab C4 scATAC-seq protocol (see the “Method details” section). A total of 25 libraries were generated, with 2 or 3 technical replicates performed for each organ.

To obtain high-quality single-cell profiles, we applied a 3-step data filtering operation to the raw data (see the “Method details” section). First, the initial filtering process excluded ~28,274 cells with a TSS <4 and unique nuclear fragments per cell <1,000, which are typically regarded as low-quality cells (Fig. S1A and B). Next, 6,638 potential doublets, typically situated between clusters (Fig. S1C and D), were filtered out. These potential doublets were predicted by ArchR, which simulates doublets from randomly real cell pairs, projects these synthetic and real cells into a shared LSI/UMAP space, and identifies real cells that repeatedly fall near simulated doublets. A binomial model provides doublet scores and enrichment values, and the highest-scoring cells were removed on a per-sample basis (Fig. S8A).

Then, the dataset was divided by organ of origin for data quality control and cell annotation. This approach was adopted to better capture organ-specific cell types and ensure more accurate cell annotation within each organ. By annotating cells separately in each organ, we were able to identify organ-specific cell types and rare cell populations, thereby improving the overall reliability of the cell type labels. In this phase, the low-quality cell clusters, comprising 3,571 cells, were defined in accordance with the established metrics and removed (Fig. S1E). Ultimately, 115,723 high-quality cells were obtained (Fig. 1B, Figs S7A and S1F–H).

To assess potential batch effects in the scATAC-seq data, we examined UMAP embeddings for each organ prior to any batch correction and colored cells by sample, TSS enrichment, and unique fragment counts (Fig. S9A–I). Across all organs, cells from different technical replicates were well mixed within clusters, with no clusters driven by low-quality cells, indicating minimal technical bias. Although small single-sample–dominant clusters were observed in spleen and pancreas, these persisted after Harmony correction group by sample, suggesting that they likely reflect biological variation rather than batch effects (Fig. S10A and B). In addition, to rule out artifacts from nonlinear embedding (such as UMAP), we examined sample relationships directly in the high-dimensional LSI space, where technical replicates from the same organ clustered closely across the first 3 LSI components, further indicating minimal batch effects (Fig. S12A and B). Accordingly, we did not apply any additional batch-correction algorithms (such as Harmony) when integrated multi-organ dataset in this study (Fig. S10C).

To define cell types, we analyzed each organ independently by performing iterative LSI-based dimensionality reduction and SNN modularity optimization-based clustering (see the “Method details” section). Clusters were annotated based on gene activity scores calculated with ArchR. We combined known cell type-specific expressed marker genes (Table S1) with genes differentially expressed between clusters in this dataset (Data S3) to comprehensively assess and assign their cell type labels (Fig. S2A–I; see the “Method details” section). The marker genes of the major cell types were visualized to assess the accuracy of the global clustering across organs and the relationships between cells from different organs (Fig. S1I). Overall, we identified 6 major cell types: epithelial, endocrine, muscle, immune, endothelial, and stromal (Fig. 1C). Epithelial cells expressed *Cdh1, Krt18*, and *Krt8*. Endocrine cells expressed *Star, Cyp19a1*, and *Cyp11a*. Muscles expressed *Acta1, Myh7*, and *Myh1*. Immune cells expressed *Cd3d, Cd4*, and *Cd163*. Endothelial cells (ECs) expressed *Flt1, Pecam1*, and *Vmf*. Stromal cells expressed *Dcn, Col1a1*, and *Col3a1* (Fig. S1I).

To visualize differences in the chromatin accessibility landscape across organs, we employed UMAP to visualize all cells and differentiate their colors according to their respective cellular origins (Fig. 1D; Data S1 and S2), and further summarized the fractions of major cell types within each organ as well as the number of nuclei from each organ (Fig. 1E). Stromal, immune, and ECs from different organs tend to cluster by cell type rather than by organ of origin or batch (Fig. 1C and D). This phenomenon has been identified in previously published data [9, 18] and may emphasize the commonality of certain cell types in different organs. Furthermore, in accordance with expectations, we observed that immune cells are the major cell types of the thymus and spleen, which are the primary immune organs *in vivo*. However, these cells tended to cluster by organ rather than by cell type (Fig. 1C and D). This phenomenon was also observed in epithelial cells from multiple organs, suggesting that the chromatin accessibility of these cells is distinctly organ-specific. For example, immune cells situated within the thymus are immature, whereas those located within the spleen are mature [19]. The epithelial cells of each organ display distinct morphological, gene expression, and functional characteristics in accordance with their environmental and functional contexts [20]. We additionally applied t-SNE alongside UMAP for visualization and quality control (Fig. S11A–D). While t-SNE can reduce apparent cell crowding and provide clearer local separation, it sacrifices global structure. More generally, all nonlinear manifold learning methods (e.g., UMAP, t-SNE, PHATE, FLE) can distort high-dimensional relationships. To mitigate this, we examined the top IterativeLSI dimensions prior to nonlinear embedding, which clearly recapitulated the biological structure of the data, including cross-organ convergence of endothelial, immune, and stromal cells and organ-specific separation of epithelial and muscle populations (Fig. S12C and D). We caution against overinterpreting 2-dimensional embeddings, which are insufficient to infer biological equivalence between cell populations, and it was sensitive to parameter choices. For example, UMAP layouts can change substantially with parameter adjustments (e.g., n_neighbors or minDist).

### Cross-organ cell type identification and comparison with mouse scRNA-seq data

**Figure 2:**
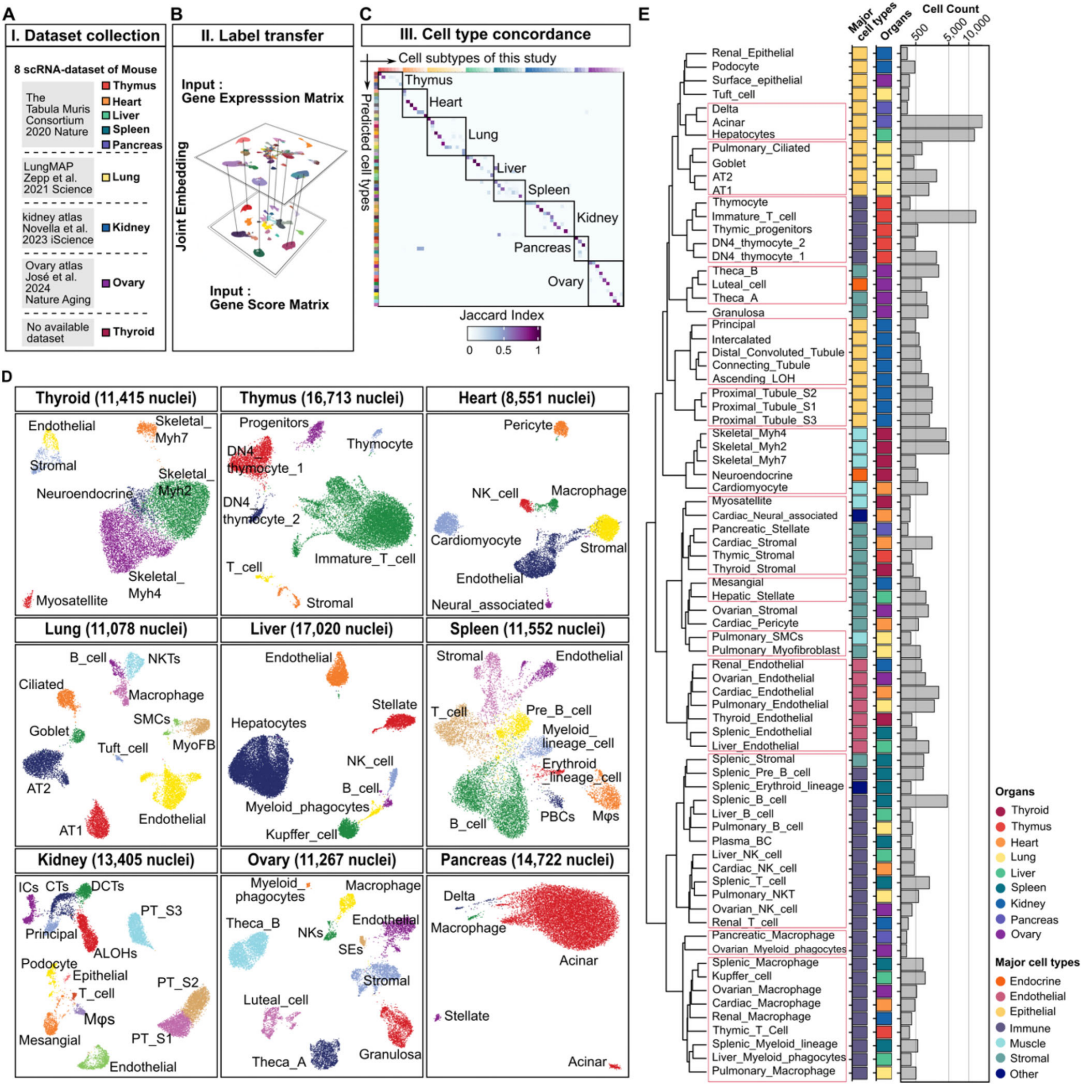
Cross-organ cell type identification and comparison via single-cell RNA sequencing data. (A) The table lists the scRNA datasets for 8 mouse organs, including the thymus, heart, liver, spleen, pancreas, lung, kidney, and ovary. No dataset is available for the thyroid. (B) A 3D plot showing the jointly embedded gene expression matrix and gene score matrix to identify anchor points across the datasets. (C) The Jaccard index was used to quantify the overlap between automated annotation and manual annotation. (D) UMAP plot showing the clusters for each organ, with cells grouped and annotated by cell subtype. (E) Dendrogram showing the hierarchical clustering of cell types based on group-level chromatin accessibility profiles derived from the Peak Matrix. Correlation distance (1 − Pearson’s *r*) and Ward.D2 linkage were used. Feature selection was performed using coefficient-of-variation filtering (top 50%). Clusters supported by approximately unbiased (AU) *P*-values ≥ 95%, estimated by multiscale bootstrap resampling (*n* = 1,000), are indicated. Aligned panels to the right summarize major cell-type classification, organ of origin for each cell subtype, and the number of nuclei per cell subtype.

In addition to utilizing gene scores for the purpose of assigning cluster identity, we can reference published scRNA datasets to facilitate the identification of cluster identity for the snATAC dataset (see the “Method details” section). A total of 8 publicly available mouse scRNA datasets were screened for matching organ origins (Fig. 2A). Unfortunately, no publicly available thyroid scRNA dataset for mice was found, and we did not integrate scRNA data for this sample at this step. We integrated the scRNA dataset with the snATAC dataset for each organ individually via cellular alignment (Fig. 2B). This method employs unsupervised identification of pairs of cells with similar biological states (defined as anchors) between datasets, followed by the joint projection of the features of the 2 modalities into a shared low-dimensional space [21].

To provide a more intuitive assessment of the results of data integration, the predicted score was used to evaluate the accuracy and confidence of integration between cells, whereas the Jaccard index was employed to assess the correlation between transferred labels of the RNA cell subtype (automated annotation) and the labels of the ATAC cell subtype that were manually annotated on the basis of gene scores (manual annotation) (Fig. S3A–H). In total, 77 cell subtypes were identified (Fig. S2A–I, Fig. S4–S4C), and a high degree of correspondence was observed between the automatic and manual cell type identification methods (Fig. 2C). This not only validates and enhances the reliability of the dataset for cell type annotation but also allows further exploration of similarities and differences between mice and rats for cell type annotation in the same organ.

To ensure transparency in the annotation process, we provided detailed descriptions of the manual cell type annotation procedures for each organ in the Supplementary Materials, including the marker genes used (Figs S13–S21), their sources, and any ambiguities encountered during annotation. During label transfer, each cell was assigned a predictedScore. For clusters with low-confidence predictions (predictedScore < 0.5), we refined cell-type labels by integrating newly published single-cell references, cluster-enriched top genes, and the top TF-motif enrichments from our snATAC-seq data. For example, in the lung dataset, we identified a small epithelial-like population with broad epithelial accessibility (e.g., *Cdh1, Krt8, Krt18*) that was initially mapped to “club cells” in the scRNA reference with low confidence (predictedScore < 0.5) (Fig. S3C). However, inspection of its top enriched genes and TF motifs, together with clear accessibility at tuft-cell markers (*Dclk1, Ascl2*) (Fig. S16L), supported annotation as “tuft cells” rather than a generic epithelial state. In the spleen dataset, a cluster mapped to “proerythroblasts” with low confidence (predictedScore < 0.5) (Fig. S3E) was instead annotated as “splenic macrophages” based on canonical macrophage markers (*Cd163, Mrc1, Csf1r, Itgam*) (Fig. S18F), concordant top genes (*Cd163, Vsir, Il10ra*), and enrichment of macrophage-lineage motifs (*Spi1/PU.1* and *Spic*). Notably, disagreements were also observed among high-confidence predictions. For instance, in the liver dataset, a population annotated as “hepatic stellate” based on accessibility at *Bmp5, Hgf*, and *Col3a1* (Fig. S17G) was mapped to “B cells” by scRNA label transfer despite a high predictedScore (>0.8) (Fig. S3D). Re-evaluation of its top genes (*Hand2, Mir143, Fendrr, Col6a2, Bmp5*, and *Prelp*) and top motifs (*ERG*/*FLI1*/*ETS1* and *ETV2*) supported a stellate identity. We also observed differences arising from annotation granularity. For example, in the heart we distinguished macrophages (*Cd163, Mrc1*, and *Csf1r*) and natural killer (NK) cells (*Ly49i3, Klrd1, Klrb1c, Il2rb*, and *Klrk1*) (Fig. S15F and G), whereas the scRNA reference assigned both to a broader leukocyte label (predictedScore > 0.8) (Fig. S3B). Overall, the correspondence between scRNA reference annotations and snATAC-seq clusters was not always strictly one-to-one, reflecting differences in reference dataset quality, annotation granularity, nomenclature conventions, and sample origin, as well as inherent biological variability introduced by different omics modalities. Therefore, we emphasize that automated label transfer should be followed by careful manual curation. Overall, all cell type annotations were rigorously curated through manual validation for each organ, integrating evidence from public reference datasets, cluster-specific top gene accessibility, and enrichment of top TF motifs (Fig. 2D; Data S3 and S4; Supplementary Materials).

The major goal of creating a cross-organ cell atlas is to gain a comprehensive understanding of cell type diversity and their relationships between different organs. To explore similarities and specificities in chromatin accessibility across cell subtypes in different organs, we conducted hierarchical clustering of cell types based on group-level chromatin accessibility profiles derived from the Peak Matrix using pvclust [22], and robustness of the observed groupings was further assessed by consensus clustering across repeated subsampling (Fig. S22A; Fig. S23A and B; Supplementary Materials). The same major cell types from different organs tend to cluster together, such as ECs, stromal cells, epithelial cells, and immune cells (Fig. 2E). This phenomenon is also observed in more finely categorized subpopulations, such as the clustering of macrophages, B cells, and T cells among the immune cells (Fig. 2E). It indicates a comparable pattern of chromatin accessibility and gene activity for these cell types across different organs.

Despite the observation that cells of the same types from disparate organs tend to cluster, we noted that certain cell types exhibit organ-specific clusters, a phenomenon that is particularly evident in epithelial cells, such as those of the liver, kidney, and lung (Fig. 2E). Although these epithelial cells share certain fundamental features, such as *Cdh1, Krt18*, and *Krt8* expression (Fig. S1I), they exhibit notable organ specificity (Fig. S4D). For example, Epithelial cells in the liver (e.g., hepatocytes) specifically express *Alb, Cyp2c7*, and *Tf*, exemplifying their functions in amino acid metabolism, energy metabolism, and detoxification [23]. Epithelial cells in the kidney (e.g., proximal tubules) specifically express *Gpx3, Lrp2, Slc34a1*, and *Slc5a10*, reflecting their unique functions in substance transport, nutrient absorption, and maintenance of homeostasis in the body [24]. Epithelial cells in the lung (e.g., alveolar type 1/2 cells) specifically express *Gprc5a, Sec14l3, Wipf1*, and *Mbip*, consistent with their multiple functions in maintaining lung homeostasis, performing gas exchange, repairing damage, and regulating immune responses [25]. These organ-specific functional requirements produce unique chromatin accessibility patterns in epithelial cells in different organs, which is reflected in the organ-based clustering observed in hierarchical clustering analyses. Notably, hierarchical clustering organizes cell types based on chromatin accessibility similarity, but the dendrogram structure does not inherently encode developmental lineage. Branches with high AU (approximately unbiased) values may reflect meaningful biological similarity, whereas branches with low AU support are unstable and should not be interpreted as biologically meaningful relationships.

In summary, our findings emphasize 2 fundamental cellular relationships between different organs: cell type specificity (similarity across organs) and organ specificity (similarity within the same organ). The same major cell types (e.g., immune cells, ECs, and stromal cells) display comparable chromatin accessibility profiles across different organs, indicating that these cells share common functional attributes in diverse tissues. In contrast, organ specificity denotes the environmental adaptation and functional differentiation of cells within a specific organ (e.g., epithelial cells), resulting in a heightened degree of similarity between different cell types within the same organ.

### Characterization of specific TF motifs across cell types in the adult rat

TFs play crucial regulatory roles in organ development, cell type differentiation, and maintenance of function. To explore differences in TF enrichment among cell types, we aggregated chromatin accessibility data from the same major cell types into “pseudobulk replicates” to improve the signal-to-noise ratio for peak calling (see the “Method details” section). In total, we identified ~450,000 open chromatin regions, encompassing candidate cis-regulatory elements such as promoters and distal regulatory regions, as well as accessible sites within intronic and exonic sequences.

**Figure 3:**
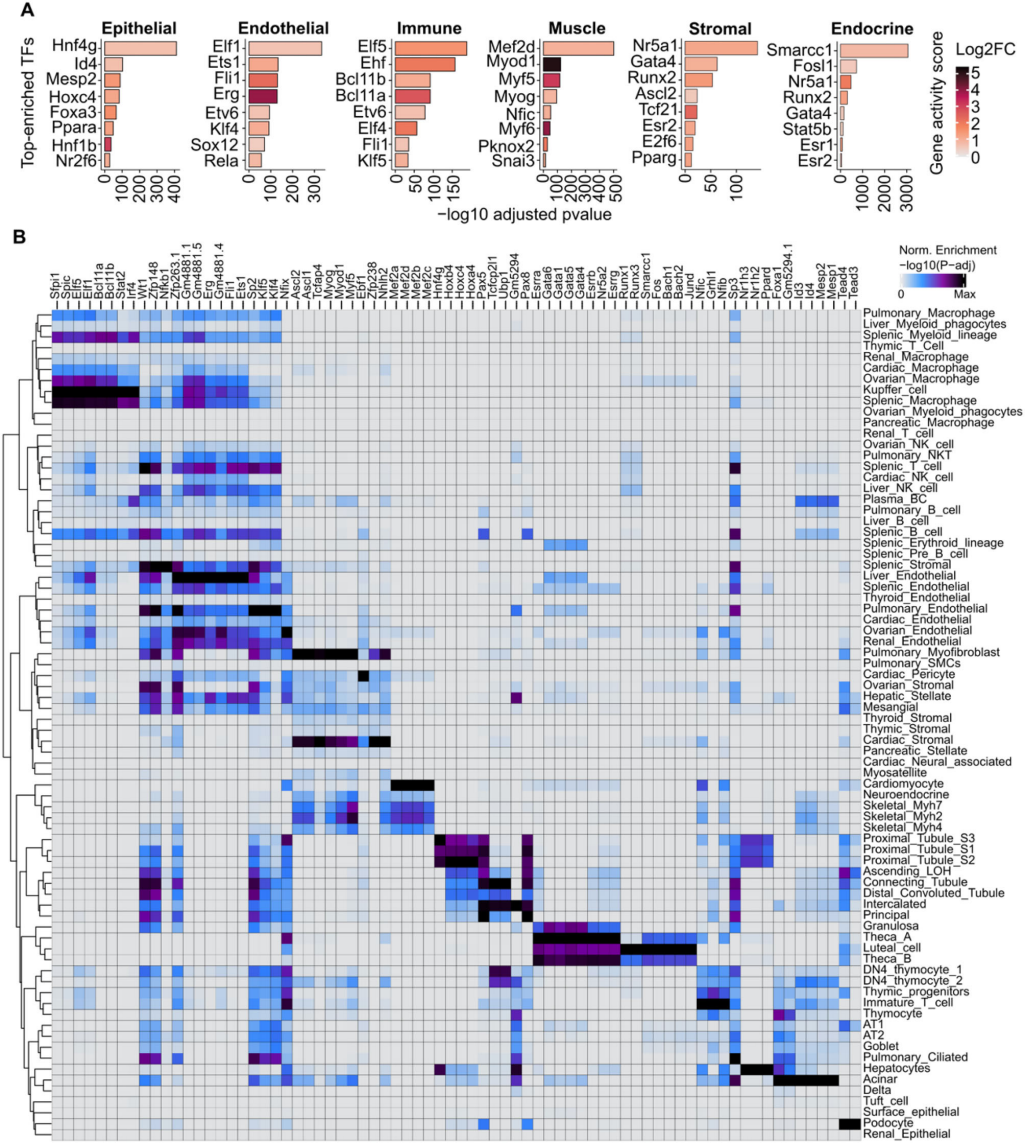
Characterization of specific TF motifs across cell types in the rat. (A) Bar plots showing the top 8 TFs (significance of differential motif activity scores) of each major cell type. The log2-fold change (log2FC) of the gene activity score for each transcription factor is shown. (B) Hypergeometric enrichment of TF motifs in marker peaks for each cell subtype. The columns represent different TFs, and the rows represent the different cell subtypes.

To identify and visualize TFs associated with chromatin accessibility in different major cell types, we combined the gene scores with motif enrichment data to elucidate the link between the activity of specific TFs and gene regulatory potential (Fig. 3A; Data S5; see the “Method details” section). Although this approach does not reflect gene expression levels directly, it reveals the relationship between chromatin accessibility and potential regulators, providing extra information regarding the specific TFs in different cell types. For epithelial cells, factors such as *Hnf4g, Foxa3*, and *Ppara*, which are essential for epithelial differentiation or metabolic regulation, were the most prominent. The ECs were found to be characterized by a significant enrichment of ETS-family TFs, including *Elf1, Ets1, Fli1*, and *Erg*. It reflects the conserved regulatory network that underlies vascular identity. In immune cells, key regulators such as *Bcl11b, Bcl11a*, and *Etv6* were found to be highly enriched, supporting the delineation of lymphoid lineages. The muscle cells exhibited robust activity of myogenic factors, including *Mef2d, Myod1, Myf5*, and *Myog*. This finding is consistent with skeletal muscle specification and differentiation. The stromal cells enriched TFs such as *Nr5a1, Gata4*, and *Runx2*, which play important roles in mesenchymal or steroidogenic cell development. For the endocrine cells, the most enriched TF motif was *Smarcc1*, followed by *Fosl1, Nr5a1, Runx2*, and *Gata4*. It is important to note that the endocrine cell population in this dataset was primarily composed of luteal and pre-luteal cells derived from the ovary. Consequently, the observed motif enrichment appears to reflect the regulatory landscape of these ovarian endocrine cell types and may not represent the full spectrum of endocrine cells from other organs. Collectively, these results validate the biological specificity of the cell-type assignments and highlight both conserved and potentially regulatory programs governing cell identity across tissues.

To identify cell subtype-specific TFs, we employed motif enrichment analysis of chromatin open regions (peaks) specific to each cell subtype. This approach enabled us to ascertain, which TF has regulatory functions within these cell types (Fig. 3B; Data S4). For example, *Sfpi1* [26, 27], a TF linked to the formation of blood cells, is specifically enriched in immune cells (especially Kupffer cells, ovarian macrophages, and splenic macrophages), indicating that it plays a pivotal role in immune cell differentiation and function. *Esrrb*, a TF associated with stem cell development, pluripotency, and germline development, was specifically enriched in certain endocrine cells of the ovary (e.g., luteal, granulosa, and theca cells), indicating that it may be involved in the regulation of ovarian function. *Foxa1* [28] and *Snai2* [29], key regulators of epithelial cell differentiation, were specifically enriched in epithelial cell clusters (e.g., acinar, AT2, and central hepatocytes), reflecting their importance in maintaining epithelial cell characteristics. *Etv2* [30], which is associated with angiogenesis and EC differentiation, was enriched not only in ECs (e.g., liver and ovarian ECs) but also in certain immune cells (e.g., splenic T cells and Kupffer cells), indicating that it may be involved in regulating the interaction between immune cells and the vascular system. These findings demonstrate that distinct TFs influence the differentiation and functional sustenance of cell types, thereby substantiating the intimate correlation between TF activity and cell fate and functional status. JASPAR is a stringently curated motif resource that is widely used in scATAC-seq studies. We therefore additionally incorporated JASPAR-based motif enrichment results across cell types to enable a complementary comparison and to more clearly delineate similarities and distinctions relative to the broader CIS-BP database (Fig. S24A and B; Supplementary Materials).

In summary, our dataset provides a valuable resource for mapping TF motif landscapes in diverse cell types across major organs in the adult rat. This information will facilitate the elucidation of gene regulatory networks in various organs and cell types and the identification of the potential roles of TFs in cell function and provide a crucial foundation for subsequent basic research and disease studies.

### Shared and organ-specific features of endothelial and stromal cells in the rat

**Figure 4:**
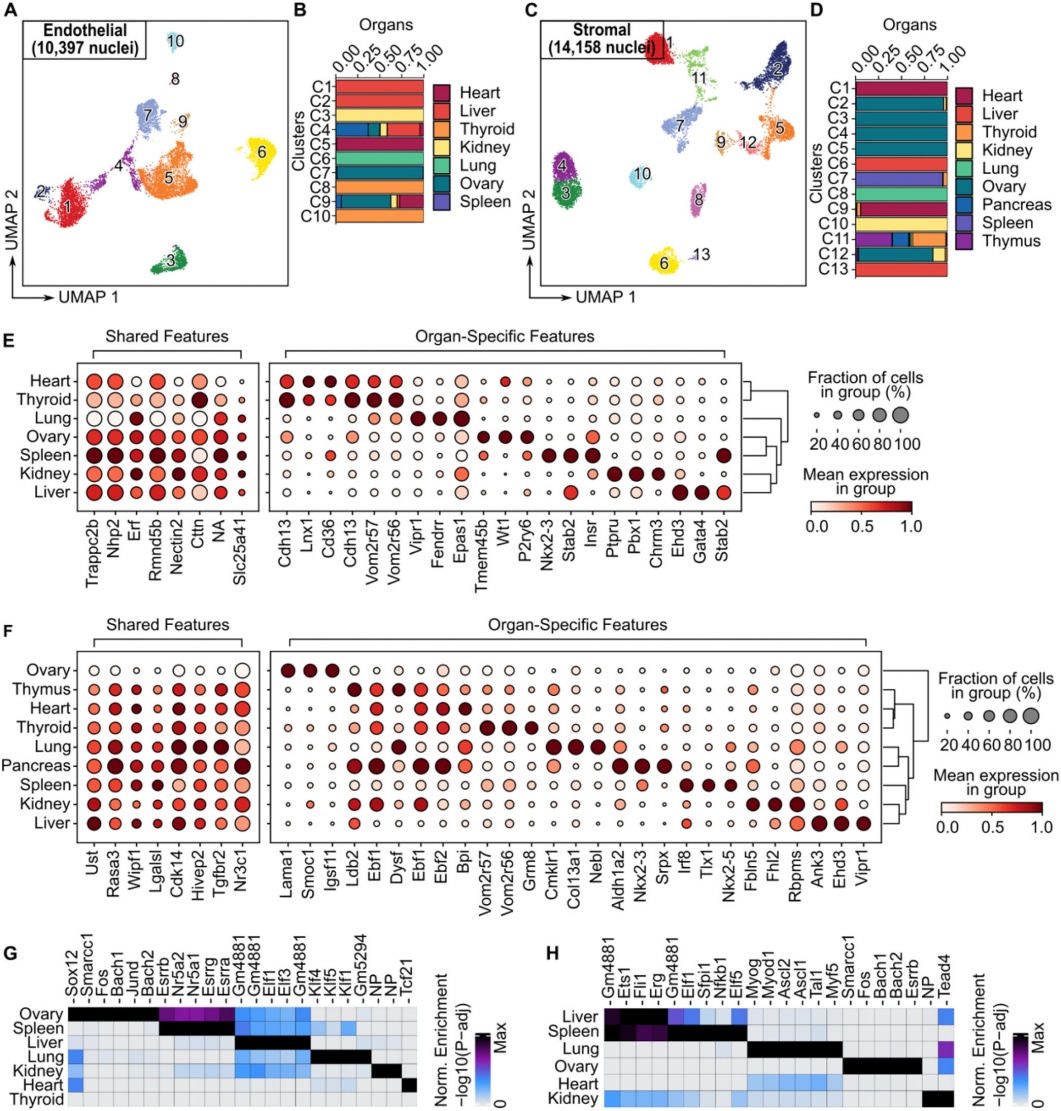
Shared and organ-specific features of endothelial and stromal cells in the adult rat. (A) UMAP plot showing the clustering of endothelial from various organs, colored according to distinct clusters. (B) Stacked bar plot showing the fraction of organs in each cluster, derived from the EC dataset. (C) Same as (A), but for stromal cells from different organs. (D) Same as (B), but for stromal cells from different organs. (E) Dot plots displaying shared and organ-specific gene scores (chromatin accessibility-inferred expression) in ECs from different organs. The size of each dot indicates the percentage of cells expressing each feature, and the color represents the average gene score. (F) Same as (E), but for stromal cells from different organs. (G) Hypergeometric enrichment of TF motifs in ECs from different organs. The columns represent different TFs, and the rows represent the different organs. (H) Same as (G), but for stromal cells from different organs.

As previously described, some cell types (e.g., ECs, stromal cells, and immune cells) exhibit a tendency to cluster across organs, suggesting a highly conserved molecular signature. However, the question remains as to whether there is still an organ-specific regulation of these widely distributed cell types at the level of chromatin accessibility. To address this question, we focused on ECs and stromal cells, which were first isolated from the complete dataset and clustered separately by dimensionality reduction (see the “Method details” section). An investigation into the chromatin accessibility distribution of typical marker genes in ECs (e.g., *Kdr, Vwf*) and stromal cells (e.g., *Dcn, Lum, Col1a1*) was undertaken, with the objective of determining the organ-specificity of open regions in proximity to these marker genes. The results of this investigation revealed significant organ-specificity in cells of different organ origins (Fig. S5A–D). The clustering results further demonstrated that most endothelial clusters were predominantly comprised of cells from a single organ, thereby reflecting the molecular characteristics associated with organ-specificity. It is notable that only a small number of clusters (e.g., C4, C9) exhibited mixed cells from multiple organs, suggesting a degree of molecular conservatism (Fig. 4A and B). The same trend was observed in stromal cells (Fig. 4C and D).

To further elucidate the molecular similarities and differences among endothelial and stromal cells across organs, we performed a systematic comparison of chromatin accessibility and gene activity (Fig. 4E and F, False Discovery Rate (FDR) ≤ 0.01 and Log2FC ≥ 1, Wilcoxon rank-sum test). We found that most of the shared genes represent broadly expressed functional genes involved in fundamental cellular processes and structural maintenance.

The same cell type exhibits distinct chromatin accessibility landscapes and organ-specific functional characteristics across different tissues. In ECs, liver-derived cells were enriched for cellular response to cytokine stimulus and tissue development, reflecting their roles in metabolism and immunomodulation. Lung ECs showed strong enrichment in developmental processes and blood vessel development, highlighting their barrier and gas exchange functions. Spleen ECs were enriched in scavenger receptor activity, consistent with their function in immune clearance. Similarly, stromal cells also exhibited organ-specific enrichment. Spleen stromal cells were associated with immune-related functions, including T cell activation and chemotaxis. Ovarian stromal cells were enriched in lipid metabolic and phosphorus metabolic processes, indicating roles in hormone production. Stromal cells from the heart and thymus showed enrichment in tube development and blood vessel morphogenesis, aligning with their developmental and vascular remodeling functions (Data S7 and S8).

The same major cell type is regulated by different TFs in different organs. The combinations of significantly enriched TFs vary across organs (Fig. 4G and H). For instance, liver ECs are enriched for *Elf1* and *Elf3*; lung ECs for *Klf4* and *Klf5*; and heart ECs for *Tcf21*. Similarly, stromal cells in the spleen are enriched for immune-associated TFs such as *Spi1* and *Nfkb1*, whereas ovarian stromal cells are enriched for *Fos, Smarcc1*, and *Esrrb*. These variations reflect the phenotypic plasticity of cells, shaped by distinct TF regulatory landscapes under the influence of organ-specific microenvironments and tissue contexts.

Macrophages play central roles in tissue homeostasis, immune surveillance, and inflammatory responses, and exhibit pronounced heterogeneity across different organs [31–34]. To systematically interrogate this heterogeneity at the regulatory level, we constructed a cross-organ macrophage snATAC-seq dataset comprising 4,176 nuclei derived from 6 organs, including liver (*n* = 1,304), spleen (*n* = 1,092), ovary (*n* = 557), lung (*n* = 516), heart (*n* = 440), and kidney (*n* = 267) (Fig. S25A; Supplementary Materials). Across this integrated dataset, chromatin accessibility at core macrophage identity genes (*Adgre1, Cd68, Mertk*, and *Csf1r*) is broadly conserved across organs, whereas, in contrast, key TF programs (e.g., *Spi1, Mafb, Cebpa*, and *Pparg::Rxra*) show pronounced tissue- and state-specific enrichment, revealing organ-adapted regulatory heterogeneity within a shared macrophage lineage (Fig. S25B). Building on these observations, chromatin accessibility-based unsupervised and consensus clustering (100 Leiden runs; PAC and dispersion metrics) resolved macrophages into 10 robust subpopulations with distinct regulatory features (Fig. S25C; Fig. S26A and B; Supplementary Materials). Specifically, these included organ-enriched tissue-resident states with accessibility at tissue-associated genes and pathways (e.g., metabolic and xenobiotic programs in liver C1, lipid–homeostatic regulation in spleen C2, extracellular matrix remodeling in ovary C7, and barrier-adjacent lipid and inflammatory programs in lung C9), as well as organ-biased but multi-organ states characterized by cell adhesion, calcium/GPCR signaling, and vascular interaction (C5, C8), and cross-organ shared immunoregulatory states (C3, C4) marked by accessible immune signaling genes and conserved regulators such as PU.1, ETS family factors, RUNX1, and AP-1. Finally, a low-feature cross-organ cluster (C6) showed minimal differential accessibility and no motif enrichment, consistent with a quiescent or transitional state, collectively supporting a hierarchical model in which conserved lineage programs are overlaid by tissue-specific regulatory modules (Fig. S25C–E; Supplementary Materials).

In summary, ECs, stromal cells, and macrophages, despite being broadly conserved across organs, exhibit distinct chromatin accessibility and gene regulatory landscapes shaped by organ-specific microenvironments. By isolating these cell types and analyzing their chromatin accessibility and TF motif activity profiles, both shared and organ-specific molecular features were identified. Shared chromatin accessibility and TF motifs provide a conserved “core” that defines cell identity, while organ-specific regulatory features reflect the adaptive specialization of cellular functions to local microenvironments. Together, these elements form a foundation for cross-organ cell type identification, annotation, and functional interpretation.

### Cross-species analysis revealed similarities and differences in gene expression patterns in human, mouse, and rat heart tissue

**Figure 5:**
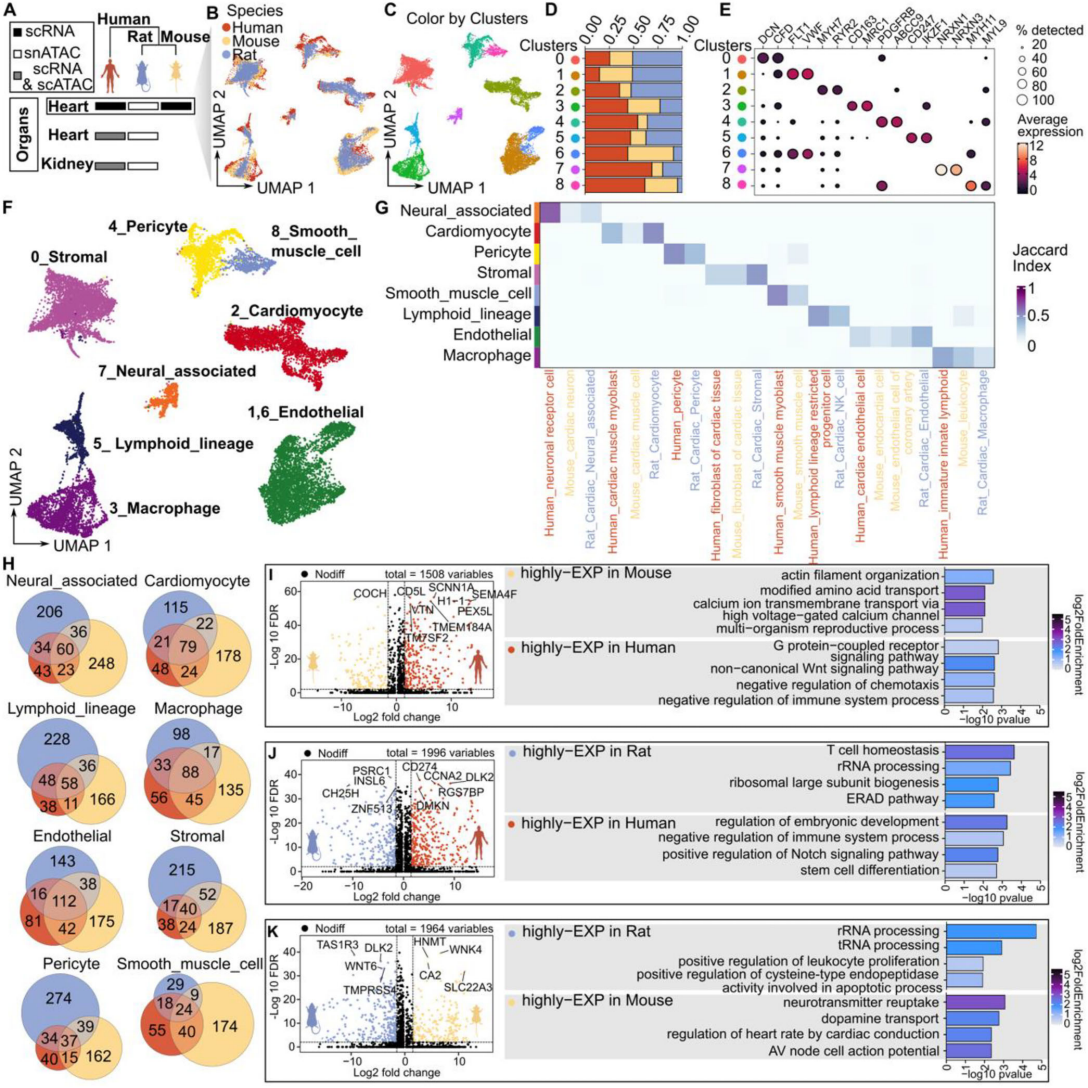
Comparative single-cell transcriptomic and epigenomic analysis across species. (A) Overview of the types of data (scRNA-seq and scATAC-seq/snATAC-seq) collected from heart and kidney tissues across humans, rats, and mice. (B) UMAP plot showing species-specific clustering. (C) UMAP plot showing the clustering of single cells in the integrated dataset, colored according to cluster. (D) Stacked bar plot showing the fraction of each cluster across species (human, mouse, and rat). The different colors represent the different species, with the same color scheme as in (B). (E) Dot plot displaying the gene expression levels of known marker genes of heart tissue across distinct clusters. The size of the dot corresponds to the percentage of cells expressing the genes in each cluster, and the color represents the average expression level. (F) UMAP plot showing the cell types of the integration dataset, colored by cell type, from the manual annotation of the data in (E). (G) Heatmap showing the similarity (Jaccard index) of cell types across different species. The rows represent the manually annotated cell type labels in the cross-species integration dataset, and the columns represent the cell type label with which the cell was annotated in the original dataset. (H) Venn diagrams showing the overlap of differentially expressed genes between species for specific cell types. (I) Volcano plot showing the results of differential gene expression analysis between human and mouse cardiomyocytes. On the right side, the bar plot shows the top biological processes that are significantly enriched among the DEGs between species. (J) Same as I but for the results of DEG analysis between human and rat cardiomyocytes. (K) Same as I but for the results of DEG analysis between mouse and rat cardiomyocytes.

One crucial application for single-cell atlases is in cross-species analyses to gain insights into the origin and evolution of different organs and cell types, the conservation and specificity of species’ gene expression patterns [35, 36], and the identification of species-specific cell types [37]. However, many current cross-species integration algorithms were originally designed for use with scRNA datasets [38]. Furthermore, no standard integration method for scATAC datasets has been established in the field [39]. To further expand the applications of rat single-cell chromatin accessibility mapping, we attempted cross-omics and cross-species integration analyses to explore the conservation and species specificity of gene expression patterns in different organs (Fig. 5A). The objective of this investigation was to ascertain whether gene scores could serve as proxies for molecular features in the context of cross-species dataset integration. To test this hypothesis, a dual-omics dataset comprising heart and kidney samples was analyzed. In brief, highly variable homologous genes were identified across species datasets and used as anchors for data integration via the Seurat V4 CCA method (see the “Method details” section). In the heart, we observed that cells from different datasets were effectively integrated, with high consistency in the clustering of the same cell types and in the gene expression levels of marker genes in the same cell type between species (Fig. S6A–E). For example, cardiomyocytes (CMs) present a high degree of similarity in their marker genes across species. Similar results were observed in the kidney (Fig. S6F–I). The results of our tests further emphasized that cell types are highly conserved across species in terms of certain important molecular mechanisms and functions. Additionally, we demonstrated that the use of gene scores as molecular features is a reliable strategy for cross-species integration of different omics data, thus further supporting the feasibility of comparative cross-species analyses.

This integration strategy was subsequently applied to the integration of human, mouse, and rat datasets, and it was observed that cells between species exhibited a high degree of intermixing in each cluster (Fig. 5B–D). We manually annotated the major cell types in the integrated cardiac dataset on the basis of known gene markers and investigated discrepancies in cell type annotations between species (Fig. 5E–F). Our findings indicated that the major cell types were identified across species, which highlights the conservation of cell types between species (Fig. 5G). Notably, however, cells annotated as pericytes in the human and rat datasets were annotated as smooth muscle cells in the mouse dataset. Similarly, cells annotated as lymphoid lineage-restricted progenitor cells in the human dataset were annotated as NK cells in the rat dataset and as leukocytes in the mouse dataset. We believe that this coclustering of cells assigned different markers in different datasets occurs largely due to differences in annotation granularity, as well as to differences in the dataset or analytical methodology on which the annotation is based. For example, the current human cardiac cell atlas includes 21 immune cell subpopulations [40]. To further explore the extent to which gene expression patterns are shared and differ across homologous cell types in different species, we performed cross-species identification of Differentially Expressed Genes (DEGs) after downsampling the integrated dataset to 200 cells per cell type (see the “Method details” section). We observed a high degree of conservation of gene expression within the same cell type across species. For example, CMs presented 79 overlapping genes, macrophages presented 88 overlapping genes, and ECs presented 112 overlapping genes (Fig. 5H; Data S9). However, most genes were enriched for expression in only one species, reflecting species specificity.

To further examine the species specificity of gene expression patterns, we concentrated our attention on CMs, which exhibit relatively high conservation across species (see the “Method details” section). In general, genes that are more highly expressed in mice (such as *COCH*) are enriched in biological processes such as actin filament organization, amino acid transport, and calcium ion transmembrane transport. On the other hand, genes that are more highly expressed in humans (such as *CD5L, SCNN1A*, and *CD274*) are associated with pathways such as G protein-coupled receptor signaling, Wnt signaling, and immune system regulation (Fig. 5I). Similarly, for the rat dataset, genes such as *PSRC1* and *DLK2* are associated with biological processes including T-cell homeostasis and leukocyte proliferation (Fig. 5J and K). These cross-species comparisons underscore specific regulatory pathways that are more active in one species than in the other, illustrating the complexity of gene expression regulation across evolutionary contexts.

In summary, our results indicate that gene scores derived from chromatin accessibility data can serve as useful proxies for gene expression in cross-species data integration analyses, allowing for the comparative assessment of biologically relevant features across species. Using this approach, we examined the conservation and divergence of cell types and gene expression profiles in human, mouse, and rat hearts. While these results provide an initial resource for studying cross-species differences in heart biology, further analyses incorporating additional data types and functional validation will be required to fully elucidate species-specific gene regulatory mechanisms.

## Discussion

Chromatin accessibility has been identified as an important prerequisite for the regulation of gene expression, as open chromatin regions provide access for TFs and the transcriptional machinery. In recent years, single-cell analysis of transposase-accessible chromatin sequencing (scATAC-seq) has emerged as a powerful tool for elucidating regulatory patterns and cellular heterogeneity. Numerous exciting single-cell chromatin accessibility profiles have been generated via this tool, providing a robust foundation for the analysis of gene regulatory programs across tissues, developmental stages, and cell types within species [8, 10, 36, 41]. However, the absence of a systematic single-cell chromatin accessibility landscape for the rat (*Rattus norvegicus*), an experimental organism frequently employed in psychological, pharmacological, and behavioral studies, motivated us to generate a single-cell chromatin accessibility landscape across organs.

In this study, we used MGI DNBelab C4 snATAC-seq to create a single-cell dataset of chromatin accessibility from 9 organs in the adult rat. This dataset of chromatin accessibility offers new insights into cellular regulation, uncovering gene activity in specific cell types and allowing systematic studies of cell types across various organs. The dataset comprises over 110,000 cells captured from 9 organs, with 77 identified cell types and hundreds of thousands of open chromatin regions. Additionally, we characterize cell type-specific and organ-specific TFs (Data S6), offering detailed insights into the regulatory landscapes of various cell types and organs. This provides a valuable framework for exploring both normal biological processes and disease states.

Cell fate and state are driven by specific TF regulatory networks. Each cell type has a set of “core TFs” that drive and maintain the transcriptional program of that cell type. For instance, ECs consistently show enrichment for ETS family motifs (e.g., *Elf1, Erg, Fli1*), CMs consistently exhibit motif enrichment for *Mef2a, Mef2d*, and *Mef2b*, and proximal tubule cells are characterized by the enrichment of *Hnf4g, Hoxb4*, and *Hoxc4* motifs. We established a chromatin-accessible cellular map across organs in adult rats and were able to query TF motif enrichment at different resolutions (cell subtype, major cell type, and organ). As a resource, it provides a framework for identifying shared and organ-specific regulatory programs, facilitating future efforts in cross-organ cell type annotation, comparison, and discovery. In this study, endothelial and stromal cells were used as examples to illustrate how the same cell type can exhibit both shared and organ-specific molecular signatures across different organs. These differences are shaped by the local microenvironment and functional demands of each organ, which in turn drive distinct chromatin accessibility landscapes and regulatory programs.

As multicellular organisms, we all originate from a single cell, which undergoes successive rounds of proliferation and differentiation to generate diverse cell types that ultimately assemble into tissues and organs with specialized physiological functions. Using snATAC-seq, our multi-organ chromatin accessibility cell atlas of the adult rat systematically delineates the regulatory element landscapes of diverse cell types across organs, revealing a high degree of regulatory diversity underlying tissue organization. Notably, although identical cell types retain shared lineage signatures, they exhibit pronounced tissue-specific TF programs, which in turn drive distinct gene expression programs and shape cell-type–specific functions within their local tissue microenvironments. For example, macrophages from different organs consistently exhibit motif activity at core lineage regulators such as *Spi1* (PU.1), as well as accessibility at canonical macrophage identity genes, including *Adgre1, Cd68, Mertk*, and *Csf1r*, confirming a shared macrophage lineage. In contrast, tissue-enriched macrophage clusters are characterized by the selective enrichment of additional TF motifs, such as *Pparg::Rxra* in lung and spleen macrophages, AP-1 family factors (*Fos/Jun, Atf3*) in ovary macrophages, and *Klf1, Klf4*, and *Cebpa* in lung-derived subsets, which are associated with distinct chromatin accessibility landscapes and functional annotations (Fig. S25). Overall, this study systematically identified and characterized combinations of candidate TFs that are shared or specific to organs and cell types. These TFs may act in a coordinated manner to participate in shaping the tissue- and cell type-specific chromatin accessibility states observed across different organs. The chromatin accessibility profiles generated in this study provide a foundational resource for future efforts to integrate single-cell transcriptomic data and construct gene regulatory network models, enabling the identification of gene modules potentially activated or repressed by tissue-enriched TFs and the biological processes they control. Furthermore, some tools (e.g., CellOracle [42]) can support *in silico* perturbation analyses using GRN and prioritize candidate regulatory interactions for experimental validation using targeted genetic perturbations, ultimately advancing a causal and predictive understanding of tissue-specific gene regulation.

There are several limitations of this work that need to be considered. First, all data presented in this study were derived from a single female adult rat at a single time point (7–8 months of age), and all organs were sampled from the same individual. This may limit the ability to capture the full range of biological diversity, including sex-specific, age-dependent, and inter-individual variation. Future studies should include both male and female rats, multiple developmental stages, and more biological replicates to ensure broader applicability and to capture rare or transient cell populations. To increase the technical stability and reproducibility of the experimental data, multiple technical replicates were conducted for each organ. Second, while entire organs were snap-frozen and homogenized for single-nucleus preparation, no sub-regional anatomical dissection was performed. As a result, the dataset represents organ-wide cellular composition and may not fully capture region-specific cell types within each organ. Third, in cross-species studies, our analyses concentrated on homologous genes and shared cell types, which may have resulted in the omission of some species-specific gene regulatory and expression patterns. We acknowledge these limitations and have sought to clearly outline them to promote scientific rigor and transparency. We hope that by openly addressing these constraints, our work will serve as a robust and reliable resource for the community and a solid foundation for future studies.

In summary, we constructed a single-nucleus multiple-organ chromatin accessibility landscape in the adult rat, which will serve as a valuable resource for investigating gene regulation and cellular diversity across multiple organs. Future studies incorporating greater cell numbers, multiple developmental stages, ages, sexes, spatial information, or experimental conditions will be needed to fully explore dynamic changes in cell states and lineages in the rat.

## Method details

### Tissue dissection and preservation

In this study, one healthy adult female SD rat of 7–8 months of age was used. The animal was purchased from Jiangsu Ailingfei Biotechnology Company Limited. The dissection of rats was conducted by the Guangzhou Institute of Biomedicine and Health, Chinese Academy of Sciences, in accordance with established protocols [43]. In summary, after executing the rats by carbon dioxide asphyxiation, we collected a total of 9 organs, including thyroid, thymus, lung, heart, liver, spleen, pancreas, kidney, and ovary, and preserved them in liquid nitrogen tanks using cryopreservation tubes. The use of rats in the relevant experimental study was approved by the Institutional Review Board on the Ethics Committee of BGI (Permit No. BGI-IRB 23050-T4).

### Single nucleus ATAC sequencing

The single-cell experiment was divided into 3 steps.

Step 1: Preparation of single-cell suspension. The preparation of single-cell suspensions was conducted in accordance with the pre-established method [16]. Briefly, each entire frozen organ was cut into small pieces and transferred into a 2 ml KIMBLE Dounce Tissue Grinder (Sigma, #D8938-1SET) containing 2 ml of ice-cold homogenizing buffer [20 mM Tris pH 8.0 (Thermo Fisher Scientific), 500 mM sucrose (BBI), 50 mM KCl (Thermo Fisher Scientific), 10 mM MgCl_2_ (MILLIPORE), 0.1% NP-40 (Roche), 1× protease inhibitor cocktail (Roche), 1% nuclease-free BSA, and 0.1 mM DTT]. The tissues were homogenized by 15 strokes of the loose Dounce pestle, and the resulting homogenate was filtered through a 70 μM cell strainer (Falcon, # 352350). Subsequently, the filtered homogenate was subjected to 5 strokes of the tight pestle to facilitate the release of nuclei, which were then filtered once more through a 30 μM cell strainer (PLURISELECT, # 43-50030-03) and transferred to a 15 ml centrifuge tube. The filtered lysate was centrifuged at 500 g for 5 min at 4°C. The pellet was then washed twice with 1 ml of ice-cold blocking buffer (1× PBS supplemented with 1% BSA), followed by another step of centrifugation at 500 g for 5 min at 4°C. Finally, the nuclei were resuspended in 50 μl of 1× PBS containing 1% BSA and counted with DAPI.

Step 2: Construction of libraries. Single-nucleus ATAC-seq libraries were prepared using the DNBelab C Series Single-Cell ATAC Library Prep Set (MGI, #1000021878) [44]. Briefly, Chromatin-open regions were indexing *in situ* within the nucleus using Tn5 enzyme, after which the labeled nuclei were loaded into a DNBelab C4 microfluidic device for droplet encapsulation. This process is based on the principle of generating nanodroplets through the flow of 2 immiscible fluids (oil and water) within a microchannel. The droplets serve as discrete reaction chambers, each containing an individual cell nucleus and the requisite biochemical reagents [45]. Subsequently, the process entails PCR pre-amplification, emulsion breaking, bead collection, DNA amplification, and purification. In summary, we generated 25 single-cell ATAC libraries, with at least 2 technical replicates for each tissue sample.

Step 3: Sequencing and alignment. All libraries were sequenced using the bipartite 50 sequencing protocol on the BGISEQ-500 and BGISEQ-T1 platforms of the National Genebank of China (CNGB), with a minimum depth of 50,000 reads per nucleus for the libraries. Raw sequencing reads were demultiplexed using PISA [46], adapters were removed using Cutadapt [47], aligned to the rat genome (Rnor_6.0) using BWA [48], and beads were called and merged using d2c. The fragment file generated from each snATAC-seq library served as the basis for downstream analysis.

### Creating a custom archRGenome for rat

In this study, the analysis of single-cell ATAC data was mainly conducted using ArchR [49] (v.1.0.2). The genome annotation was created using the *createGenomeAnnotation*, the rat genome as “BSgenome.Rnorvegicus.UCSC.rn6.” The gene annotation was generated with *createGeneAnnotation* function, using the TxDb (TxDb.Rnorvegicus.UCSC.rn6.refGene) and OrgDb (org.Rn.eg.db) objects to extract gene-related data, such as TSS, exons, and genes. Finally, the created genome and gene annotations were saved to an RData file. It should be noted that the custom ArchRGenome needs to match the reference genome used to generate the fragments file, which is crucial to avoid errors, such as issues in recognizing TSSs when creating ArrowFiles.

### Preprocessing

The data preprocessing primarily involved 3 key steps:

First, the removal of low-quality nuclei. We used the *createArrowFiles* function to generate Arrow files from the fragment data. The data were filtered to exclude cells with fewer than 1,000 unique nuclear fragments per cell or fewer than 4 TSS enrichment scores per cell, as these metrics are crucial for ensuring that only nuclei with adequate chromatin accessibility and transcriptional activity are retained. During the quality control process, it was observed that many cells present in the thyroid samples exhibited a TSS value below 4, which typically indicates these cells are likely dead or dying, as their nucleosomes have begun to unravel. This unraveling can lead to random transposition events across the entire genome. Despite these cells having high levels of fragmentation (indicating potential chromatin activity), we were still removed from the analysis because they were classified as low-quality cells due to the low TSS enrichment score. This is notwithstanding the possibility that this could occur in certain biological states, such as dormant cells or specific cell types that naturally exhibit low levels of gene expression variability. Nevertheless, we were confident that we had taken the requisite precautions in sample processing. To guarantee the quality and accuracy of subsequent analyses, we retained the cells with high TSS enrichment.

Second, the elimination of potential doublets. We applied the *addDoubletScores* function to infer potential doublets, with the *k* parameter set to 10 to determine the number of nearest neighbors considered in the doublet detection process. Subsequently, we applied the *filterDoublets* function to remove doublets, with the filterRatio parameter set to 1. The filterRatio parameter controls the stringency of doublet removal; a higher filterRatio results in more cells potentially being identified and removed as doublets. For example, with a dataset of 5,000 cells, the maximum number of cells that could be removed as doublets is computed as filterRatio × 5000^2/100000, which can be simplified to filterRatio × 5000 × 0.05.

Third, the exclusion of low-quality cell clusters. To enhance the accuracy of quality control, we used *subsetArchRProject* function to divide the entire dataset by organ for preprocessing. We used a for loop in R to perform the same operations (such as dimensionality reduction, clustering, visualization, marker gene analysis, and heatmap generation) individually for each organ. For the dimensionality reduction and clustering, we employed *addIterativeLSI* and *addClusters* functions in ArchR, setting the parameters as follows: iterations at 3, resolution at c(0.2,0.4), varFeatures at 25,000, dimensions ranging from 1 to 30, and a resolution of 0.2 for clustering. For the visualization, we employed *addUMAP* in ArchR, setting the parameters as follows: nNeighbors at 60 and minDistat at 0.6. For the marker gene analysis, we employed *getMarkerFeatures* and *getMarkers* functions in ArchR, setting the parameters as follows: useMatrix at GeneScoreMatrix, groupBy at Clusters, testMethod at Wilcoxon, and cutoff at FDR ≤ 0.01 and Log2FC ≥ 1. For the heatmap generation, we employed *plotMarkerHeatmap* functions in ArchR, setting the parameters as follows: cutoff at FDR ≤ 0.01 and Log2FC ≥ 1. During the viewing of the UMAP plot with Gene Scores Marker Heatmap, we manually identified low-quality clusters and removed them from the dataset based on the following rules:

- The cluster did not express distinctly specific genes.
- The number of cells of cluster <50.
- The same cell cluster simultaneously expresses marker genes typical of multiple cell types and has a high doublet score.

After removal of low-quality cell clusters, we again performed the same operation as described above with the same parameters individually for each organ until the final clustering results in composite quality requirements.

### Annotation

To manually identify organ-specific cell types and states, we annotated cells within each organ dataset separately before integration, we employed *plotEmbedding* and *plotGroups* functions to visualize known marker genes of cell types individually for each organ, setting the parameters as follows: colorBy at GeneScoreMatrix, groupBy at Clusters. The list of known marker genes of cell types utilized in this study was derived from the aggregation of data from our previous investigation. Each cluster was meticulously annotated based on the established practices and insights provided by previous researchers [50].

- If a cluster expresses <3 markers related to a specific cell type with low expression, it is judged that the cluster does not belong to that cell type.
- If multiple clusters co-express >3 markers related to the same cell type with high expression; it is judged that these clusters all belong to the same cell type.
- If a cluster expresses multiple markers of different cell types and the first 10 marker genes of different cell types significantly mark the same cluster; the cluster is judged to be doublet and removed.

Additionally, we comprehensively considered the highly expressed gene profiles of each cluster while identifying each cell type. For the same cluster that unambiguously expresses marker genes of 2 cell types separately by contour region, the value of resolution in the *addClusters* function was appropriately increased to more accurately identify the cell types. We recommend trying multiple parameters and observing the cluster divisions when performing dimensionality reduction and cluster to balance the clustering granularity and biological interpretability.

### Label transfer

To help with cluster identity assignment, we used *addGeneIntegrationMatrix* function in ArchR to directly align cells from snATAC-seq with cells from scRNA-seq by comparing the snATAC-seq gene score matrix with the scRNA-seq gene expression matrix. This function converts the gene score matrix from the ArchR project into a Seurat object and uses *FindTransferAnchors* function from the Seurat [21] package, which allows you to perform CCA-based integration between the snATAC-seq data and the scRNA-seq data.

To compare predicted cell types from scRNA datasets with manually annotated cell types in snATAC datasets, we created a confusion matrix using the *confusionMatrix* function, calculated the similarity between the 2 sets of labels using the *jaccardIndex* function, added row and column annotations with the *HeatmapAnnotation* function, and then plotted the heatmap with customized aesthetics using the *heatmap* function.

Considering the current dearth of rat cross-organ single-cell RNA datasets, we employed data derived from mouse for integration in the present study. The source information and download links for the mouse scRNA-seq datasets used in the label transfer step are provided in the STAR^★^Methods section of the Supplementary Materials. All the above datasets were obtained from the online website CZ CELLxGENE: Discover [51, 52], with thanks to them for providing free access to convenient, standardized scRNA dataset downloads to facilitate the exploration and sharing of single-cell datasets.

### Subcluster label assignment to full project

As described above, after identifying organ-specific cell types and low-quality clusters in each organ separately, we mapped these label matches back to the entire dataset for further analysis. For redoing dimensionality reduction and clustering, we used *addIterativeLSI* and *addClusters* functions, setting the parameters as follows: iterations at 2, resolution at 0.6, varFeatures at 25,000, dimensions ranging from 1 to 30, and a resolution of 0.2 for clustering.

To visualize the expression of marker genes for each cell type in each organ, we employed the *dotplot* function in Scanpy [53]. We first need to convert the GeneScoreMatrix from ArchR project into a Seurat object. We got the gene score matrix from ArchR project using the *getMatrixFromProject* function and created Seurat object using the *CreateAssayObject* and *CreateSeuratObject* function. The Seurat object was normalized (LogNormalize method), and variable features were identified (vst method with 2,000 features) using *NormalizeData* and *FindVariableFeatures* functions. It was then saved in h5Seurat format using *SaveH5Seurat* function and converted into an h5ad format using *Convert* function, which is compatible with AnnData, often used in Python for further single-cell RNA-seq analysis. Additionally, the metadata from the ArchR project was extracted using *getCellColData* function and saved as a CSV file. In the Scanpy analysis, we used the default parameters to identify differentially expressed genes between cell subtypes and then filtered these results by a minimum fold change of 1. We subset each organ’s data to generate the expression of marker genes using *sc.pl.dotplot* function.

To compute hierarchical clustering of cell subtypes, we employed *sc.tl.dendrogram* functions using “complete” linkage and optimal ordering to understand the hierarchical relationships between different cell subtypes.

### Peaks calling

We created pseudo-bulk replicates, a bulk ATAC-seq experiment, allowing for more robust downstream analyses by reducing noise and enabling statistical comparisons, based on major cell types in the dataset using *addGroupCoverages* function in ArchR. To call peaks using MACS2 [54], we utilized *addReproduciblePeakSet* function to generate reproducible peak set across cells grouped by major cell types in ArchR.

### Motif enrichments

To determine which TFs (proteins that bind to specific DNA sequences to regulate gene expression) are responsible for binding events, we utilized *addMotifAnnotations* function to add motif information to the ArchR project, setting the parameters as follows: motifSet at cisbp [55], species at *Mus musculus*. Although rat-specific motifs are ideal, the limited availability justifies the use of mouse motifs. Many TF binding sites are conserved across closely related species, so using mouse motifs can still provide meaningful insights.

For the motif enrichment analysis, we employed *getMarkerFeatures, peakAnnoEnrichment*, and *plotEnrichHeatmap* functions in ArchR, setting the parameters as follows: useMatrix at PeakMatrix, groupBy at cell subtypes or organs, testMethod at Wilcoxon and cutoff at FDR ≤ 0.1 and Log2FC ≥ 0.5.

For identifying and visualizing the most enriched TFs associated with chromatin accessibility in different major cell types, we identified marker genes (via GeneScoreMatrix) and motif enrichments (via PeakMatrix) across major cell types and merged gene scores with motif enrichment data, linking TF activity to specific gene expression patterns.

### Analysis endothelial and stromal cell across organs

To identify and analyze endothelial and stromal cell diversity and regulatory elements across organs, we first extracted endothelial and stromal cell from the full dataset and recalled specific peaks for cell subtypes (consistent as described in the previous methods but grouped by cell subtype). Dimensionality reduction was then performed using *addIterativeLSI* function, setting the parameters as follows: iterations at 2, resolution at 2.0, varFeatures at 25,000, useMatrix at PeakMatrix, and dimensions ranging from 1 to 35, and a resolution of 0.2 for clustering. Visualized in low-dimensional space using *addUMAP* function with nNeighbors at 40 and minDist at 0.4.

To investigate the molecular heterogeneity of the same cell type across different organs by identifying both organ-specific and conserved gene signatures, we extracted GeneScoreMatrix from the ArchR object using the *getMatrixFromProject* function in ArchR and the gene names were assigned as row names. We then converted it into a Seurat assay object using *CreateAssayObject* function and wrapped it in a Seurat object using *CreateSeuratObject* function. To convert Seurat object to anndata, we first saved Seurat object in the Seurat format (.h5Seurat) using the *SaveH5Seurat* function and then converted it to the annData format (.h5ad) using the *Convert* function from the SeuratDisk package (v0.0.0.90).

Differential expression analysis was performed using Scanpy’s rank_genes_groups() with Wilcoxon rank-sum test across organs. Significantly upregulated genes (log2FC ≥ 1, FDR < 0.01) were identified per organ, and top markers were visualized. Conserved marker genes were defined as those differentially expressed in at least 4 organs in ECs dataset or 7 organs in stromal cells dataset and ranked by average *z*-score-normalized expression across organs. Functional enrichment analysis using gProfiler was then performed separately for conserved and organ-specific marker genes.

To ensure robustness in cross-organ motif enrichment analysis, cell populations with fewer than 500 cells were excluded, as small cell counts can lead to unreliable peak calling and inflated false positives in downstream enrichment analyses. To identify and visualize TF motif enrichment across different organs based on chromatin accessibility data, differentially accessible peaks were first identified using getMarkerFeatures with a Wilcoxon test, accounting for TSS enrichment and fragment count biases. These peak sets were then used for motif enrichment analysis via peakAnnoEnrichment, with significant motifs defined by FDR ≤ 0.01 and log2FC ≥ 1.

### Cross-species integration

The process of cross-species data integration can be divided into 3 steps.

Step 1: Data preprocessing. For the rat dataset, we extracted the GeneScoreMatrix from an ArchR project using *getMatrixFromProject* function and converted it into a Seurat object using *CreateAssayObject* and *CreateSeuratObject* functions. To ensure consistency of gene names across species datasets, we converted gene symbols in the Seurat object to Ensembl IDs using the *bitr* function from the clusterProfiler [56] package, leveraging the org.Rn.eg.db database. To ensure comparability of cell types in cross-species data integration and comparative analyses, we screened for homologous cell types across species and down-sampled according to cell type to ensure that the number of cells of each cell type in the analyses is in a reasonable range (e.g., a minimum of 50 and a maximum of 1,000).

Step 2: Homologous substitution of gene names. We connected to the Ensembl database using the biomaRt [57] package and converted rat and mouse gene symbols to their human homologs.

Step 3: Cross-species integration. We began by compiling the data into a list of Seurat objects. To normalize and standardize the data, we applied the *SCTransform* function with the glmGamPoi method to each dataset in the list, ensuring that all variable genes were retained (return.only.var.genes = F). Next, we selected 3,000 integration features across the datasets using the *SelectIntegrationFeatures* function, which identifies the most consistent and variable genes for integration. We then prepared the data for integration using *PrepSCTIntegration* and identified anchors across the datasets with *FindIntegrationAnchors*, using the selected features and the first 30 principal components (dims = 1:30) to align the datasets. We integrated the data using the *IntegrateData* function, which combined the datasets into a single Seurat object normalized with the SCT method. After integration, we reduced the dimensionality of the data using PCA with *RunPCA*, and then visualized it in a lower-dimensional space using UMAP with *RunUMAP*. Finally, we identified cell clusters by *FindNeighbors* and clustering them with the Louvain algorithm (*FindClusters*), setting the resolution to 0.3 to control the cluster size. To identify the shared and unique DEGs among human, mouse, and rat, we subsetted into separate datasets for human, mouse, and rat and down sampled each dataset to 200 cells per cell type to ensure comparable cell numbers across species. We then identified DEGs for each cell type within each species using the *FindAllMarkers* function with default parameters. To visualize the overlap of DEGs among human, mouse, and rat, we generated a Venn diagram using the eulerr package [58]. To perform differential expression analysis across species (human vs. mouse, human vs. rat, mouse vs. rat), we used the *FindMarkers* function with default parameters. Volcano plots were generated using the EnhancedVolcano package [59]. Gene ontology (GO) enrichment analysis was performed using topGO package [60].

The source information and download links for the human and mouse scRNA-seq/scATAC-seq datasets used for cross-species integration are provided in the STAR^★^Methods section of the Supplementary Materials. All the above datasets were obtained from the online website CZ CELLxGENE: Discover, with thanks to them for providing free access to convenient, standardized scRNA dataset downloads to facilitate the exploration and sharing of single-cell datasets.

## Availability of source code and requirements

Project name: scATACseq-Rat-organs

Project homepage: https://github.com/ronghai-li/scATACseq-Rat-organs

License: MIT license

Operating system: macOS

Programming language: R and Python

Package management: CRAN, Bioconductor and Conda

Hardware requirements: Tested on a laptop with 8-core CPU and 24 GB RAM

## Additional files

**Supplementary Material**. This file includes Supplementary Material and Supplementary Methods, and Figs S1–S26.

**Supplementary Table S1**. The main marker genes were used for annotation in this paper, related to Figs S1 and S2.

**Supplementary Data S1**. The metadata of dataset in this paper, related to Figs 1 and S1.

**Supplementary Data S2**. The data frame that contains the UMAP coordinates for each cell in the ArchR project, related to Fig. 1.

**Supplementary Data S3**. The data frame contains information about top 20 genes identified for each cell subtype, related to Fig. 2.

**Supplementary Data S4**. The data frame contains the results of specific TF binding motifs that are significantly enriched in the accessible chromatin regions (peaks) associated with different cell subtypes, related to Fig. 3.

**Supplementary Data S5**. The data frame contains the results of specific TF binding motifs that are significantly enriched in the accessible chromatin regions (peaks) associated with different major cell types, related to Fig. 3.

**Supplementary Data S6**. The data frame contains the results of specific TF binding motifs that are significantly enriched in the accessible chromatin regions (peaks) associated with different organs, related to Fig. 3.

**Supplementary Data S7**. The data frame contains the results of GO term enrichment of organ-specific gene scores across multiple organs in endothelial cells, related to Fig. 4.

**Supplementary Data S8**. The data frame contains the results of GO term enrichment of organ-specific gene scores across multiple organs in stromal cells, related to Fig. 4.

**Supplementary Data S9**. A comprehensive dataset that consolidates differential expression analysis results across multiple subclasses and species, related to Fig. 5.

## Abbreviations

DEGs: Differentially Expressed Genes; FDR: False Discovery Rate.

## Funding

This work was supported by the Shenzhen Key Laboratory of Single-Cell Omics (ZDSYS20190902093613831).

## Supplementary Material

giag013_Supplemental_Files

giag013_Authors_Response_To_Reviewer_Comments_original_submission

giag013_GIGA-D-25-00323_original_submission

giag013_GIGA-D-25-00323_Revision_1

giag013_Reviewer_1_Report_original_submission Reviewer 1 -- 9/15/2025

giag013_Reviewer_1_Report_Revision_1 Reviewer 1 -- 12/22/2025

giag013_Reviewer_2_Report_original_submission Reviewer 2 -- 9/23/2025

giag013_Reviewer_2_Report_Revision_1 Reviewer 2 -- 12/30/2025

giag013_Reviewer_3_Report_original_submission Reviewer 3 -- 9/25/2025

giag013_Reviewer_3_Report_Revision_1 Reviewer 3 -- 1/16/2026

## Data Availability

The data supporting the findings of this study have been deposited into CNGB Sequence Archive (CNSA) [61] of China National GeneBank DataBase (CNGBdb) [62] with accession number CNP0006032. All raw sequencing data have been deposited in the NCBI Sequence Read Archive (SRA) under BioProject accession PRJNA1312332 (study: SRP620606). Other supporting data are available in GigaDB [63].
