# Supplementary material for: Single-nucleus multiple-organ chromatin accessibility landscape in the adult rat
Source: Gigascience. 2026 Feb 3;15:giag013. doi: 10.1093/gigascience/giag013 (PMC12954174; doi:10.1093/gigascience/giag013)
Supplement: giag013_GIGA-D-25-00323_original_submission [file giag013_giga-d-25-00323_original_submission.pdf]

GigaScience

Single-nucleus multiple-organ chromatin accessibility landscape in the adult rat

--Manuscript Draft--

**Manuscript Number:**	GIGA-D-25-00323
**Full Title:**	Single-nucleus multiple-organ chromatin accessibility landscape in the adult rat
**Article Type:**	Research
**Funding Information:**	Shenzhen Key Laboratory of Single-Cell Omics (ZDSYS20190902093613831) — Not applicable
**Abstract:**	The chromatin accessibility landscape is the basis of cell-specific gene expression. We generated a multi organ, single-nucleus chromatin accessibility landscape from the model organism Rattus norvegicus. For this single-cell atlas, we constructed 25 libraries via snATAC-seq from nine organs in the rat, with a total of over 110,000 cells. Cell classification integrating gene activity scores with known marker genes identified 77 cell types, which were strongly correlated with those in published mouse single-cell transcriptome atlases. We further investigated the enrichment of cell type- and organ-specific transcription factors (TFs), Shared and organ-specific features of endothelial and stromal cells, and the conservation and specificity of gene regulatory programs across species. Together, these findings provide a valuable foundation for dissecting tissue-specific regulatory logic and for advancing cross-organ and cross-species cell type annotation and functional inference in the rat model.
**Corresponding Author:**	Yue Yuan, Ph.D.<br>BGI Group<br>Hangzhou, ZJ CHINA
**Corresponding Author Secondary Information:**	
**Corresponding Author's Institution:**	BGI Group
**Corresponding Author's Secondary Institution:**	
**First Author:**	Ronghai Li
**First Author Secondary Information:**	
**Order of Authors:**	Ronghai Li<br>Shanshan Duan<br>Qiuting Deng<br>Wen Ma<br>Chang Liu<br>Peng Gao<br>Li Lu<br>Yue Yuan
**Order of Authors Secondary Information:**	
**Additional Information:**	
**Question**	**Response**
Are you submitting this manuscript to a special series or article collection?	No
**Experimental design and statistics**	Yes

Powered by Editorial Manager® and ProduXion Manager® from Aries Systems Corporation

Full details of the experimental design and statistical methods used should be given in the Methods section, as detailed in our Minimum Standards Reporting Checklist. Information essential to interpreting the data presented should be made available in the figure legends.<br><br>Have you included all the information requested in your manuscript?	
**Resources**<br><br>A description of all resources used, including antibodies, cell lines, animals and software tools, with enough information to allow them to be uniquely identified, should be included in the Methods section. Authors are strongly encouraged to cite Research Resource Identifiers (RRIDs) for antibodies, model organisms and tools, where possible.<br><br>Have you included the information requested as detailed in our Minimum Standards Reporting Checklist?	Yes
**Availability of data and materials**<br><br>All datasets and code on which the conclusions of the paper rely must be either included in your submission or deposited in publicly available repositories (where available and ethically appropriate), referencing such data using a unique identifier in the references and in the "Availability of Data and Materials" section of your manuscript.<br><br>Have you have met the above requirement as detailed in our Minimum Standards Reporting Checklist?	Yes
GigaScience has policies and guidelines in place for the use of generative AI-	No

writing tools such as ChatGPT. If you have used such writing tools to assist with writing the manuscript this must be declared and cited in the text. Authors should not list AI-writing tools and other AI-assisted technologies as an author or co-author and should acknowledge that they are fully responsible for text generated or refined by AI-writing tools.<p><br><br>A summary of use (particularly in the introduction or among methods) needs to be included at the end of the paper, and the outputs should also be included as a supplementary file hosted in GigaDB or other open repositories. Please read our guidelines for more information. <p><br><br>By submitting to GigaScience, you are aware of the journal's AI-writing tools policy, and if you have declared use of such tools below, you have acknowledged this where appropriate in your manuscript and have made a summary of use and outputs available. <p><br><b>AI-assisted writing tools have been used in the preparation of this manuscript?	

Powered by Editorial Manager® and ProduXion Manager® from Aries Systems Corporation

Manuscript Click here to access/download;Manuscript;Manuscript_Rat_atlas.pdf

Single-nucleus multiple-organ chromatin accessibility landscape in the adult rat

Ronghai Li[1], Shanshan Duan[2,3], Qiuting Deng[1], Wen Ma[1], Chang Liu[1,4,5], Peng Gao[4,6], Li Lu[4,7,8]✉ & Yue Yuan[2]✉

[1]BGI Research, Shenzhen 518083, China

[2]BGI Research, Hangzhou 310030, China

[3]College of Life Sciences, University of Chinese Academy of Sciences, Beijing 100049, China

[4]Shanxi Medical University - BGI Collaborative Center for Future Medicine, Shanxi Medical University, Taiyuan 030001, China

[5]Shenzhen Proof-of-Concept Center of Digital Cytopathology, BGI Research, Shenzhen 518083, China

[6]BGI, Shenzhen 518083, China

[7]School of Basic Medical Sciences, Shanxi Medical University, Taiyuan 030001, China

[8]Key Laboratory of Cellular Physiology of Chinese Ministry of Education, Shanxi Medical University, Taiyuan 030001, China

✉e-mail: luli@sxmu.edu.cn, yuanyue@genomics.cn

Abstract

The chromatin accessibility landscape is the basis of cell-specific gene expression. We generated a multi organ, single-nucleus chromatin accessibility landscape from the model organism *Rattus norvegicus*. For this single-cell atlas, we constructed 25 libraries via snATAC-seq from nine organs in the rat, with a total of over 110,000 cells. Cell classification integrating gene activity scores with known marker genes identified 77 cell types, which were strongly correlated with those in published mouse single-cell transcriptome atlases. We further investigated the enrichment of cell type- and organ-specific transcription factors (TFs), Shared and organ-specific features of endothelial and stromal cells, and the conservation and specificity of gene regulatory programs across species. Together, these findings provide a valuable foundation for dissecting tissue-specific regulatory logic and for advancing cross-organ and cross-species cell type annotation and functional inference in the rat model.

Keywords

Single-nucleus ATAC-seq • Rat cell atlas • Single-cell chromatin accessibility • Epigenomics • Transcription factor • Single-cell analysis • Cellular classification • Cross-organ analysis • Cross-species analysis

Introduction

The Human Cell Atlas (HCA) project aims to create a comprehensive reference cell atlas of all cells in the human body (the basic unit of life). This will serve as a basis for understanding human health and for diagnosing, monitoring and treating disease.

However, a key scientific question is what insights can be gained from cell atlases. To date, studies of cell atlases have advanced our understanding of anatomy, development, physiology, pathology, and intra- and intercellular regulation at a new level of granularity. They have also advanced our understanding of cellular diversity, revealing the cellular compositions of complex tissues and organs and how cells interact with each other in states of health and disease[1]. The development of single-cell and spatial genomics technologies, as well as the corresponding algorithms, has enabled the mapping of cells across omics, organs, species, developmental states, and diseases with unprecedented resolution. This has facilitated the systematic probing of biological questions related to cell type, spatial location, developmental trajectory, fate determination, the tumour microenvironment, and molecular mechanisms, among others. These advances provide powerful new tools and will open new avenues for clinical medicine, especially in precision medicine and personalized treatment.

The rat (*Rattus norvegicus*) has long played a key role in scientific research as a model animal for studies of disease mechanisms, drug development and physiology. In recent years, the development and application of single-cell technology has facilitated considerable advances in the mapping of single cells within organs in the rat. For example, single-cell atlases of individual organs (e.g., the brain[2,3], kidney[4], and testes[5]) have provided valuable insights into the cellular compositions and gene regulatory mechanisms of these organs. Furthermore, such studies are progressively expanding to produce more systemic single-cell atlases encompassing multiple organs[6], elucidating the cellular heterogeneity and synergistic interactions between different organs. In

parallel, cross-species single-cell mapping analyses of individual organs are also being conducted. For example, recent studies utilizing scRNA-seq of lung tissues derived from mice, rats, pigs and humans have revealed evolutionarily conserved mechanisms in alveolar intercellular communication[7]. In our previous work, we constructed single-nucleus chromatin accessibility landscape of the rat brain and spinal cord, providing valuable resources for understanding region- and cell type-specific gene regulation in the mammalian nervous system[8,9]. Building upon this foundation, the present study extends our efforts to profile chromatin accessibility across nine major organs in the rat. This multi-organ dataset not only supplements and extends our previous dataset but also provides a valuable resource for the research community, supporting studies of epigenomic diversity and gene regulation across different organs and cell types in the rat.

Results

Single-nucleus multiple-organ chromatin accessibility landscape in the adult rat

Here, we report the cellular composition and chromatin accessibility landscape of multiple organs in the rat. The dataset consists of single-cell epigenomic data from 115,723 nuclei isolated from nine organs (namely, the thyroid, thymus, heart, lung, liver, spleen, kidney, pancreas and ovary) of a single female Sprague–Dawley rat aged 7–8 months (Figure 1A). The organs were dissociated into single-nuclear suspensions in accordance with preestablished methods and then subjected to snATAC-seq via the standard MGI DNBelab C4 scATAC-seq protocol (STAR Methods). A total of 25 libraries were generated, with two or three technical replicates performed for each organ.

To obtain high-quality single-cell profiles, we applied a three-step data filtering operation to the raw data (STAR Methods). First, the initial filtering process excluded approximately 28,274 cells with a TSS less than 4 and unique nuclear fragments per cell less than 1,000, which are typically regarded as low-quality cells (Figures S1A and S1B). Next, 6,638 potential doublets, typically situated between clusters (Figures S1C and S1D), were filtered out. These doublets were predicted with an algorithmic approach that involved mixing the reads from thousands of individual cell combinations to synthesize simulated duplexes, which are most likely to be doublets.

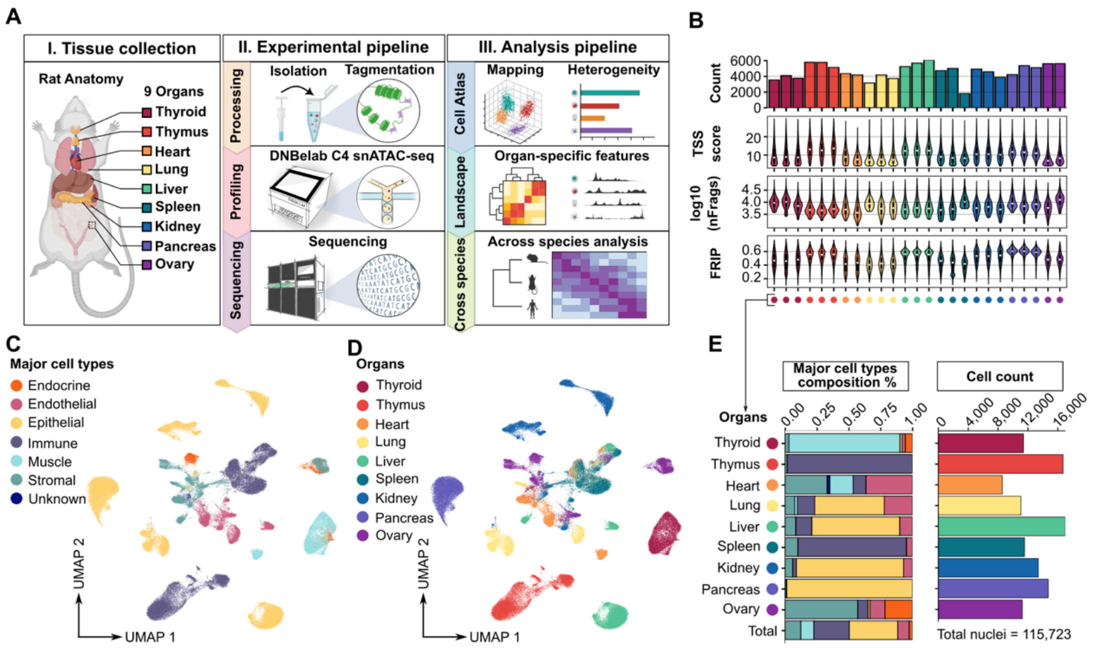

Figure 1. Cross-organ single-nucleus ATAC sequencing atlas of an adult rat. **A**. Schematic of the study design, including the tissue collection, experimental pipeline, and analysis pipeline steps (Created in BioRender.com). **B**. Quality control metrics across the library, with the same colour used to represent technical replicates of the same organ. The bar plot shows the number of nuclei in each library. Violin plots show the transcription start site (TSS) enrichment scores, log10 unique nuclear fragment counts, and fraction of reads in peaks (FRIP) for each library. **C**. Cross-organ snATAC-seq atlas. The UMAP plot shows all the nuclei identified in this study, coloured according to major cell type. **D**. Same as C but coloured according to organ. **E**. Stacked bar plot showing the fraction of major cell types in each organ, with the total proportions of major cell types in this study shown in the bottom column (left). The bar

plot shows the number of nuclei for each organ, and the total number of nuclei in this study is shown at the bottom (right).

Then, the dataset was divided by organ of origin for data quality control and cell annotation. This approach was adopted to better capture organ-specific cell types and ensure more accurate cell annotation within each organ. By annotating cells separately in each organ, we were able to identify organ-specific cell types and rare cell populations, thereby improving the overall reliability of the cell type labels. In this phase, the low-quality cell clusters, comprising 3,571 cells, were defined in accordance with the established metrics and removed (Figure S1E). Ultimately, 115,723 high-quality cells were obtained (Figures 1B and S1F-S1H).

To define cell types, we analysed each organ independently by performing iterative LSI-based dimensionality reduction and SNN modularity optimization-based clustering (STAR Methods). Clusters were annotated based on gene activity scores calculated with ArchR. We combined known cell type-specific expressed marker genes (Table S1) with genes differentially expressed between clusters in this dataset (Data S3) to comprehensively assess and assign their cell type labels (Figures S2A-S2I; STAR Methods). The marker genes of the major cell types were visualized to assess the accuracy of the global clustering across organs and the relationships between cells from different organs (Figure S1I). Overall, we identified 6 major cell types: epithelial, endocrine, muscle, immune, endothelial and stromal (Figure 1C). Epithelial cells expressed *Cdh1*, *Krt18* and *Krt8*. Endocrine cells expressed *Star*, *Cyp19a1* and *Cyp11a*. Muscles expressed *Acta1*, *Myh7* and *Myh1*. Immune cells expressed *Cd3d*, *Cd4* and

Cd163. Endothelial cells expressed *Flt1*, *Pecam1* and *Vmf*. Stromal cells expressed *Dcn*, *Col1a1* and *Col3a1* (Figure S1I).

To visualize differences in the chromatin accessibility landscape across organs, we employed UMAP to visualize all cells and differentiate their colours according to their respective cellular origins (Figure 1D; Data S1 and S2). Stromal cells, immune cells, and endothelial cells from different organs tend to be clustered together (i.e., by cell type) rather than clustered according to the organ of origin or sample batch (Figure 1E). This phenomenon has been identified in previously published data[10,11] and may emphasize the commonality of certain cell types in different organs. Furthermore, in accordance with expectations, we observed that immune cells are the major cell types of the thymus and spleen, which are the primary immune organs in vivo. However, these cells tended to cluster by organ rather than by cell type (Figure 1E). This phenomenon was also observed in epithelial cells from multiple organs, suggesting that the chromatin accessibility of these cells is distinctly organ specific. For example, immune cells situated within the thymus are immature, whereas those located within the spleen are mature[12]. The epithelial cells of each organ display distinct morphological, gene expression and functional characteristics in accordance with their environmental and functional contexts[13].

Cross-organ cell type identification and comparison with mouse single-cell RNA sequencing data

In addition to utilizing gene scores for the purpose of assigning cluster identity, we can reference published scRNA datasets to facilitate the identification of cluster identity

for the snATAC dataset (STAR Methods). A total of 8 publicly available mouse scRNA datasets were screened for matching organ origins (Figure 2A). Unfortunately, no publicly available thyroid scRNA dataset for mice was found, and we did not integrate scRNA data for this sample at this step. We integrated the scRNA dataset with the snATAC dataset for each organ individually via cellular alignment (Figure 2B). This method employs unsupervised identification of pairs of cells with similar biological states (defined as anchors) between datasets, followed by the joint projection of the features of the two modalities into a shared low-dimensional space[14].

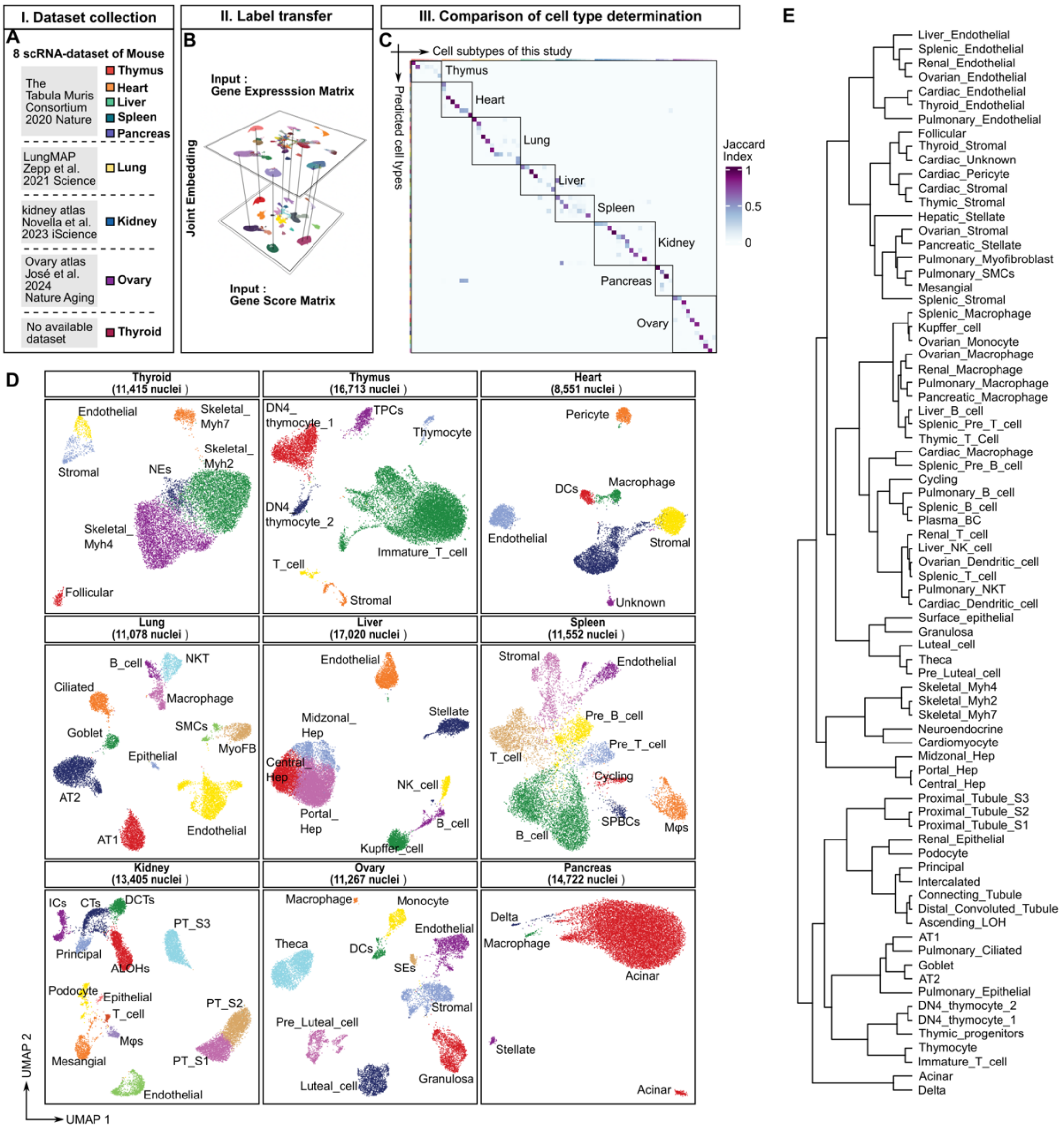

Figure 2. Cross-organ cell type identification and comparison via single-cell RNA sequencing data. **A**. The table lists the scRNA datasets for eight mouse organs, including the thymus, heart, liver, spleen, pancreas, lung, kidney, and ovary. No dataset is available for the thyroid. **B.** A 3D plot showing the jointly embedded gene expression matrix and gene score matrix to identify anchor points across the datasets. **C**. The Jaccard index was used to quantify the overlap between automated annotation and manual annotation. **D**. UMAP plot showing the clusters for each organ, with cells coloured and labelled by cell subtype. **E**. Hierarchical clustering dendrogram illustrating the relationships and similarities between the cell subtypes across organs.

To provide a more intuitive assessment of the results of data integration, the predicted score was used to evaluate the accuracy and confidence of integration between cells, whereas the Jaccard index was employed to assess the correlation between transferred labels of the RNA cell subtype (automated annotation) and the labels of the ATAC cell subtype that were manually annotated on the basis of gene scores (manual annotation) (Figures S2A–S2H). In total, 77 cell subtypes were identified (Figure 2D, Figures S3A–S3I), and a high degree of correspondence was observed between the automatic and manual cell type identification methods (Figure 2C). This not only validates and enhances the reliability of the dataset for cell type annotation but also allows further exploration of similarities and differences between mice and rats for cell type annotation in the same organ.

The major goal of creating a cross-organ cell atlas is to gain a comprehensive understanding of cell type diversity and their relationships between different organs. We employed the gene score matrix to cluster average gene expression profiles from 77 cell subtypes, with the objective of exploring similarities and specificities in chromatin accessibility across cells in different organs (Figure 2E). The same major cell types from different organs tend to cluster together, such as endothelial cells,

stromal cells, epithelial cells, and immune cells (Figure S4A and S4B). This phenomenon is also observed in more finely categorized subpopulations, such as the clustering of macrophages, B cells, and T cells among the immune cells. It indicates a comparable pattern of chromatin accessibility and gene activity for these cell types across different organs (Figure S4C).

Despite the observation that cells of the same types from disparate organs tend to cluster, we noted that certain cell types exhibit organ-specific clusters, a phenomenon that is particularly evident in epithelial cells, such as those of the liver, kidney, and lung (Figure S4A and S4B). Although these epithelial cells share certain fundamental features, such as *Cdh1*, *Krt18*, and *Krt8* expression (Figure S1I), they exhibit notable organ specificity (Figure S4D). For example, Epithelial cells in the liver (e.g., hepatocytes) specifically express *Alb*, *Cyp2c7*, and *Tf*, exemplifying their functions in amino acid metabolism, energy metabolism, and detoxification[15]. Epithelial cells in the kidney (e.g., proximal tubules) specifically express *Gpx3*, *Lrp2*, *Slc34a1*, and *Slc5a10*, reflecting their unique functions in substance transport, nutrient absorption, and maintenance of homeostasis in the body[16]. Epithelial cells in the lung (e.g., alveolar type 1/2 cells) specifically express *Gprc5a*, *Sec14l3*, *Wipf1* and *Mbip*, consistent with their multiple functions in maintaining lung homeostasis, performing gas exchange, repairing damage and regulating immune responses[17]. These organ-specific functional requirements produce unique chromatin accessibility patterns in epithelial cells in different organs, which is reflected in the organ-based clustering observed in hierarchical clustering analyses.

In summary, our findings emphasize two fundamental cellular relationships between different organs: cell type specificity (similarity across organs) and organ specificity (similarity within the same organ). The same major cell types (e.g., immune cells, endothelial cells and stromal cells) display comparable chromatin accessibility profiles across different organs, indicating that these cells share common functional attributes in diverse tissues. In contrast, organ specificity denotes the environmental adaptation and functional differentiation of cells within a specific organ (e.g., epithelial cells), resulting in a heightened degree of similarity between different cell types within the same organ.

Characterization of specific TF motifs across cell types in the adult rat

Transcription factors (TFs) play crucial regulatory roles in organ development, cell type differentiation and maintenance of function. To explore differences in TFs enrichment among cell types, we aggregated chromatin accessibility data from the same major cell types into 'pseudobulk replicates' to improve the signal–to–noise ratio for peak calling (STAR Methods). In total, we identified approximately 450,000 open chromatin regions, encompassing candidate cis-regulatory elements such as promoters and distal regulatory regions, as well as accessible sites within intronic and exonic sequences.

To identify and visualize TFs associated with chromatin accessibility in different major cell types, we combined the gene scores with motif enrichment data to elucidate the link between the activity of specific TFs and gene regulatory potential (Figure 3A; Data S5; STAR Methods). Although this approach does not reflect gene expression

levels directly, it reveals the relationship between chromatin accessibility and potential regulators, providing extra information regarding the specific TFs in different cell types. For epithelial cells, factors such as *Hnf4g*, *Foxa3*, and *Ppara*, which are essential for epithelial differentiation or metabolic regulation, were most prominent. The endothelial cells were found to be characterized by a significant enrichment of ETS-family TFs, including *Elf1*, *Ets1*, *Fli1*, and *Erg*. It reflects the conserved regulatory network that underlies vascular identity. In immune cells, key regulators such as *Bcl11b*, *Bcl11a*, and *Etv6* were found to be highly enriched, supporting the delineation of lymphoid lineages. The muscle cells exhibited robust activity of myogenic factors, including *Mef2d*, *Myod1*, *Myf5*, and *Myog*. This finding is consistent with skeletal muscle specification and differentiation. The stromal cells enriched TFs such as *Nr5a1*, *Gata4*, and *Runx2*, which play important roles in mesenchymal or steroidogenic cell development. For the endocrine cells, the most enriched TF motif was *Smarcc1*, followed by *Fosl1*, *Nr5a1*, *Runx2*, and *Gata4*. It is important to note that the endocrine cell population in this dataset was primarily composed of luteal and pre-luteal cells derived from the ovary. Consequently, the observed motif enrichment appears to reflect the regulatory landscape of these ovarian endocrine cell types and may not represent the full spectrum of endocrine cells from other organs. Collectively, these results validate the biological specificity of the cell type assignments and highlight both conserved and potentially regulatory programs governing cell identity across tissues.

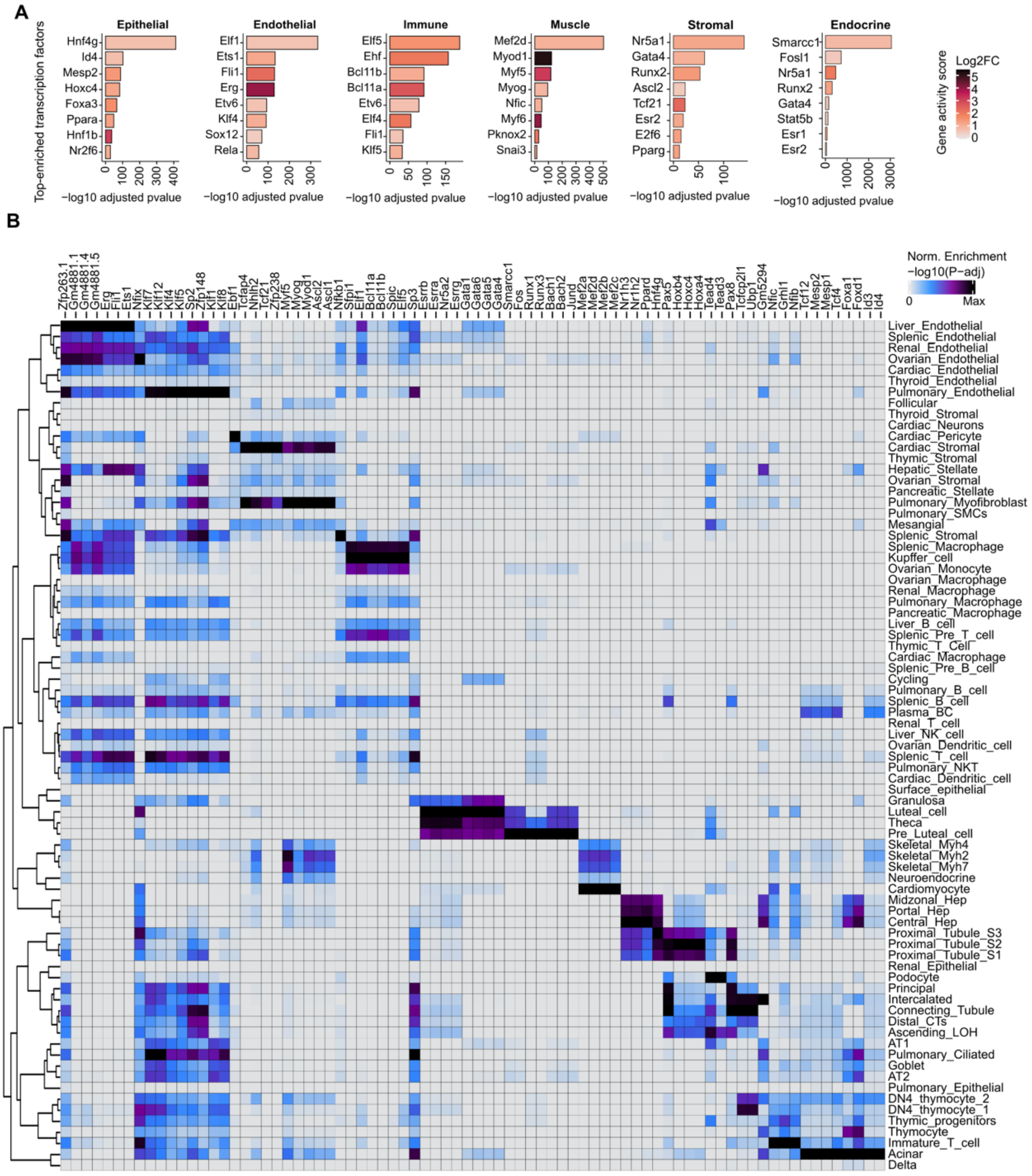

Figure 3. Characterization of specific TF motifs across cell types in the rat. **A.** Bar plots showing the top 8 TFs (significance of differential motif activity scores) of each major cell type. The colour of the bars indicates the log2-fold change (log2FC) in the gene activity score, with darker red representing a higher degree of differential activity. **B.** Hypergeometric enrichment of TF motifs in marker peaks for each cell subtype. The columns represent different TFs, and the rows represent the different cell subtypes.

To identify cell subtype-specific TFs, we employed motif enrichment analysis of chromatin open regions (peaks) specific to each cell subtype. This approach enabled us to ascertain which TF has regulatory functions within these cell types (Figure 3B; Data

S4). For example, *Sfpi1*[18,19], a TF linked to the formation of blood cells, is specifically enriched in immune cells (especially Kupffer cells, ovarian monocytes, and splenic macrophages), indicating that it plays a pivotal role in immune cell differentiation and function. *Esrrb*, a TF associated with stem cell development, pluripotency, and germline development, was specifically enriched in certain endocrine cells of the ovary (e.g., luteal, granulosa, and theca cells), indicating that it may be involved in the regulation of ovarian function. *Foxa1*[20] and *Snai2*[21], key regulators of epithelial cell differentiation, were specifically enriched in epithelial cell clusters (e.g., acinar, AT2, and central hepatocytes), reflecting their importance in maintaining epithelial cell characteristics. *Etv2*[22], which is associated with angiogenesis and endothelial cell differentiation, was enriched not only in endothelial cells (e.g., liver and ovarian endothelial cells) but also in certain immune cells (e.g., splenic T cells and Kupffer cells), indicating that it may be involved in regulating the interaction between immune cells and the vascular system. These findings demonstrate that distinct TFs influence the differentiation and functional sustenance of cell types, thereby substantiating the intimate correlation between TF activity and cell fate and functional status.

In summary, our dataset provides a valuable resource for mapping transcription factor motif landscapes in diverse cell types across major organs in the adult rat. This information will facilitate the elucidation of gene regulatory networks in various organs and cell types and the identification of the potential roles of TFs in cell function and provide a crucial foundation for subsequent basic research and disease studies.

Shared and organ-specific features of endothelial and stromal cells in the rat

As previously described, some cell types (e.g. endothelial cells, stromal cells, and immune cells) exhibit a tendency to cluster across organs, suggesting a highly conserved molecular signature. However, the question remains as to whether there is still an organ-specific regulation of these widely distributed cell types at the level of chromatin accessibility. To address this question, we focused on endothelial cells and stromal cells, which were first isolated from the complete dataset and clustered separately by dimensionality reduction (STAR Methods). An investigation into the chromatin accessibility distribution of typical marker genes in endothelial cells (e.g., *Kdr*, *Vwf*) and stromal cells (e.g., *Dcn*, *Lum*, *Col1a1*) was undertaken, with the objective of determining the organ-specificity of open regions in proximity to these marker genes. The results of this investigation revealed significant organ-specificity in cells of different organ origin (Figure S5A-S5D). The clustering results further demonstrated that most endothelial clusters were predominantly comprised of cells from a single organ, thereby reflecting the molecular characteristics associated with organ-specificity. It is notable that only a small number of clusters (e.g., C4, C9) exhibited mixed cells from multiple organs, suggesting a degree of molecular conservatism (Figure 4A and 4B). The same trend was observed in stromal cells (Figure 4C and 4D).

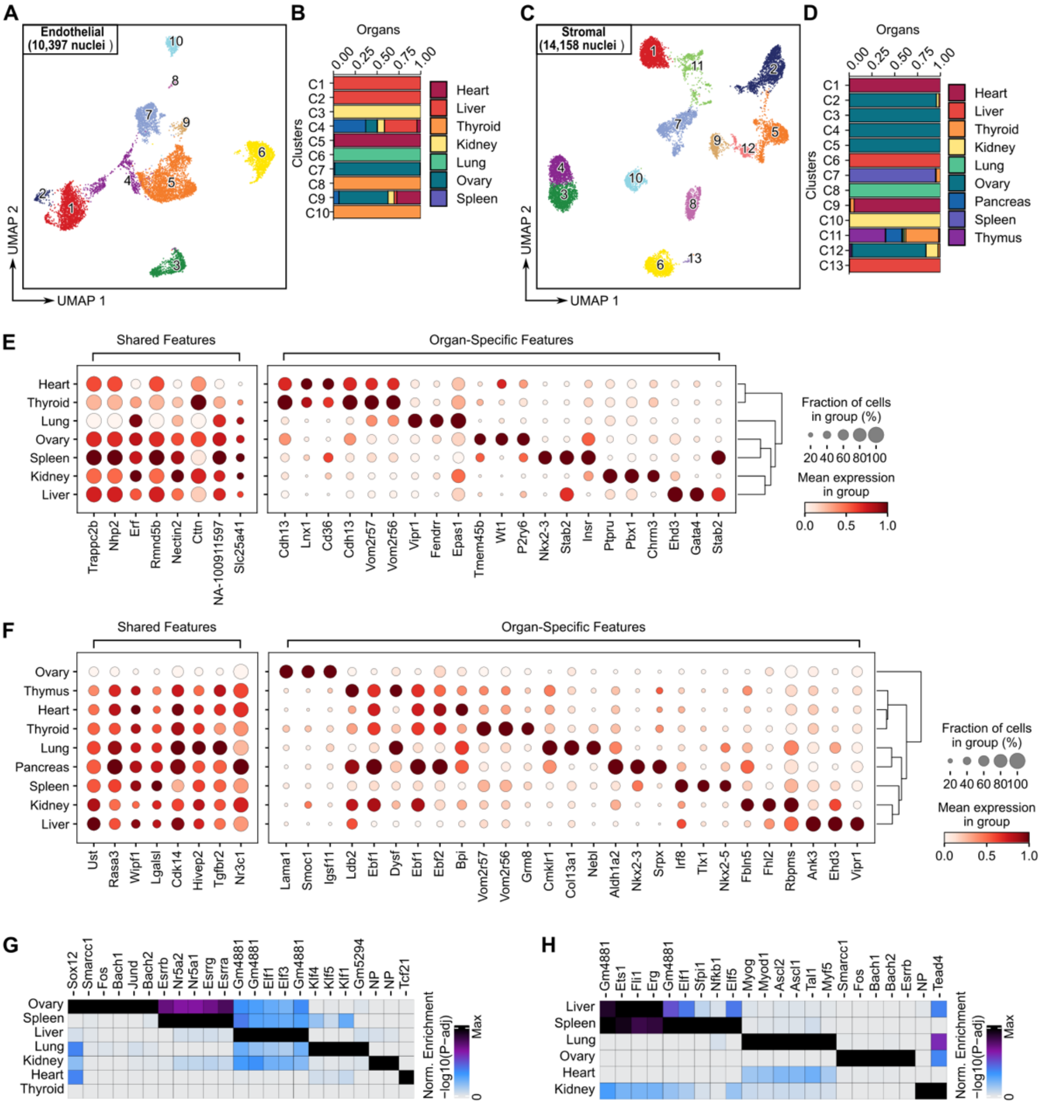

Figure 4. Shared and organ-specific features of endothelial and stromal cells in the adult rat. **A.** UMAP plot showing the clustering of endothelial from various organs, coloured according to distinct clusters. **B.** Stacked bar plot showing the fraction of organ in each cluster, derived from the endothelial cell dataset. **C.** Same as A, but for stromal cell from different organs. **D.** Same as B, but for stromal cell from different organs. **E.** Dot plots displaying shared and organ-specific gene scores (chromatin accessibility-inferred expression) in endothelial cells from different organs. The size of each dot indicates the percentage of cells expressing each feature, and the colour represents the average gene score. **D.** Same as E, but for stromal cell from different organs. **G.** Hypergeometric enrichment of TF motifs in endothelial cells from different organs. The columns represent different TFs, and the rows represent the different organs. **H.** Same as G, but for stromal cell from different organs.

To further elucidate the molecular similarities and differences among endothelial and stromal cells across organs, we performed a systematic comparison of chromatin accessibility and gene activity (Figure 4E and 4F, FDR <= 0.01 & Log2FC >= 1, Wilcoxon rank-sum test). We found that most of the shared genes represent broadly expressed functional genes involved in fundamental cellular processes and structural maintenance.

The same cell type exhibits distinct chromatin accessibility landscapes and organ-specific functional characteristics across different tissues. In endothelial cells, liver-derived cells were enriched for cellular response to cytokine stimulus and tissue development, reflecting their roles in metabolism and immunomodulation. Lung endothelial cells showed strong enrichment in developmental processes and blood vessel development, highlighting their barrier and gas exchange functions. Spleen endothelial cells were enriched in scavenger receptor activity, consistent with their function in immune clearance. Similarly, stromal cells also exhibited organ-specific enrichment. Spleen stromal cells were associated with immune-related functions, including T cell activation and chemotaxis. Ovarian stromal cells were enriched in lipid metabolic and phosphorus metabolic processes, indicating roles in hormone production. Stromal cells from the heart and thymus showed enrichment in tube development and blood vessel morphogenesis, aligning with their developmental and vascular remodeling functions (Data S7 and S8).

The same major cell type is regulated by different TFs in different organs. The combinations of significantly enriched TFs vary across organs (Figure 4G and 4H).

For instance, liver endothelial cells are enriched for *Elf1* and *Elf3*; lung endothelial cells for *Klf4* and *Klf5*; and heart endothelial cells for *Tcf21*. Similarly, stromal cells in the spleen are enriched for immune-associated TFs such as *Spi1* and *Nfkb1*, whereas ovarian stromal cells are enriched for *Fos*, *Smarcc1*, and *Esrrb*. These variations reflect the phenotypic plasticity of cells, shaped by distinct TF regulatory landscapes under the influence of organ-specific microenvironments and tissue contexts.

In summary, Endothelial and stromal cells, despite being broadly conserved across organs, exhibit distinct chromatin accessibility and gene regulatory landscapes shaped by organ-specific microenvironments. By isolating these cell types and analyzing their chromatin accessibility and gene activity profiles, both shared and organ-specific molecular features were identified. Shared chromatin accessibility and TF motifs provide a conserved “core” that defines cell identity, while organ-specific regulatory features reflect the adaptive specialization of cellular functions to local microenvironments. Together, these elements form a foundation for cross-organ cell type identification, annotation, and functional interpretation.

Cross-species analysis revealed similarities and differences in gene expression patterns in human, mouse and rat heart tissue

One crucial application for single-cell atlases is in cross-species analyses to gain insights into the origin and evolution of different organs and cell types, the conservation and specificity of species' gene expression patterns[23,24], and the identification of species-specific cell types[25]. However, many current cross-species integration

algorithms were originally designed for use with scRNA datasets[26]. Furthermore, no standard integration method for scATAC datasets has been established in the field[27]. To further expand the applications of rat single-cell chromatin accessibility mapping, we attempted cross-omics and cross-species integration analyses to explore the conservation and species specificity of gene expression patterns in different organs (Figure 5A). The objective of this investigation was to ascertain whether gene scores could serve as proxies for molecular features in the context of cross-species dataset integration. To test this hypothesis, a dual-omics dataset comprising heart and kidney samples was analysed. In brief, highly variable homologous genes were identified across species datasets and used as anchors for data integration via the Seurat V4 CCA method (STAR Methods). In the heart, we observed that cells from different datasets were effectively integrated, with high consistency in the clustering of the same cell types and in the gene expression levels of marker genes in the same cell type between species (Figure S6A-S6E). For example, CMs present a high degree of similarity in their marker genes across species. Similar results were observed in the kidney (Figure S6F-S6I). The results of our tests further emphasized that cell types are highly conserved across species in terms of certain important molecular mechanisms and functions. Additionally, we demonstrated that the use of gene scores as molecular features is a reliable strategy for cross-species integration of different omics data, thus further supporting the feasibility of comparative cross-species analyses.

This integration strategy was subsequently applied to the integration of human, mouse, and rat datasets, and it was observed that cells between species exhibited a high

degree of intermixing in each cluster (Figure 5B-5D). We manually annotated the major cell types in the integrated cardiac dataset on the basis of known gene markers and investigated discrepancies in cell type annotations between species (Figure 5E-5F). Our findings indicated that the major cell types were identified across species, which highlights the conservation of cell types between species (Figure 5G). Notably, however, cells annotated as pericytes in the human and rat datasets were annotated as smooth muscle cells in the mouse dataset. Similarly, cells annotated as lymphoid lineage-restricted progenitor cells in the human dataset were annotated as dendritic cells in the rat dataset and as leukocytes in the mouse dataset. We believe that this coclustering of cells assigned different markers in different datasets occurs largely due to differences in annotation granularity, as well as to differences in the dataset or analytical methodology on which the annotation is based. For example, the current human cardiac cell atlas includes 21 immune cell subpopulations[28]. To further explore the extent to which gene expression patterns are shared and differ across homologous cell types in different species, we performed cross-species identification of DEGs after downsampling the integrated dataset to 200 cells per cell type (STAR Methods). We observed a high degree of conservation of gene expression within the same cell type across species. For example, CMs presented 79 overlapping genes, macrophages presented 88 overlapping genes, and ECs presented 112 overlapping genes (Figure 5H; Data S9). However, most genes were enriched for expression in only one species, reflecting species specificity.

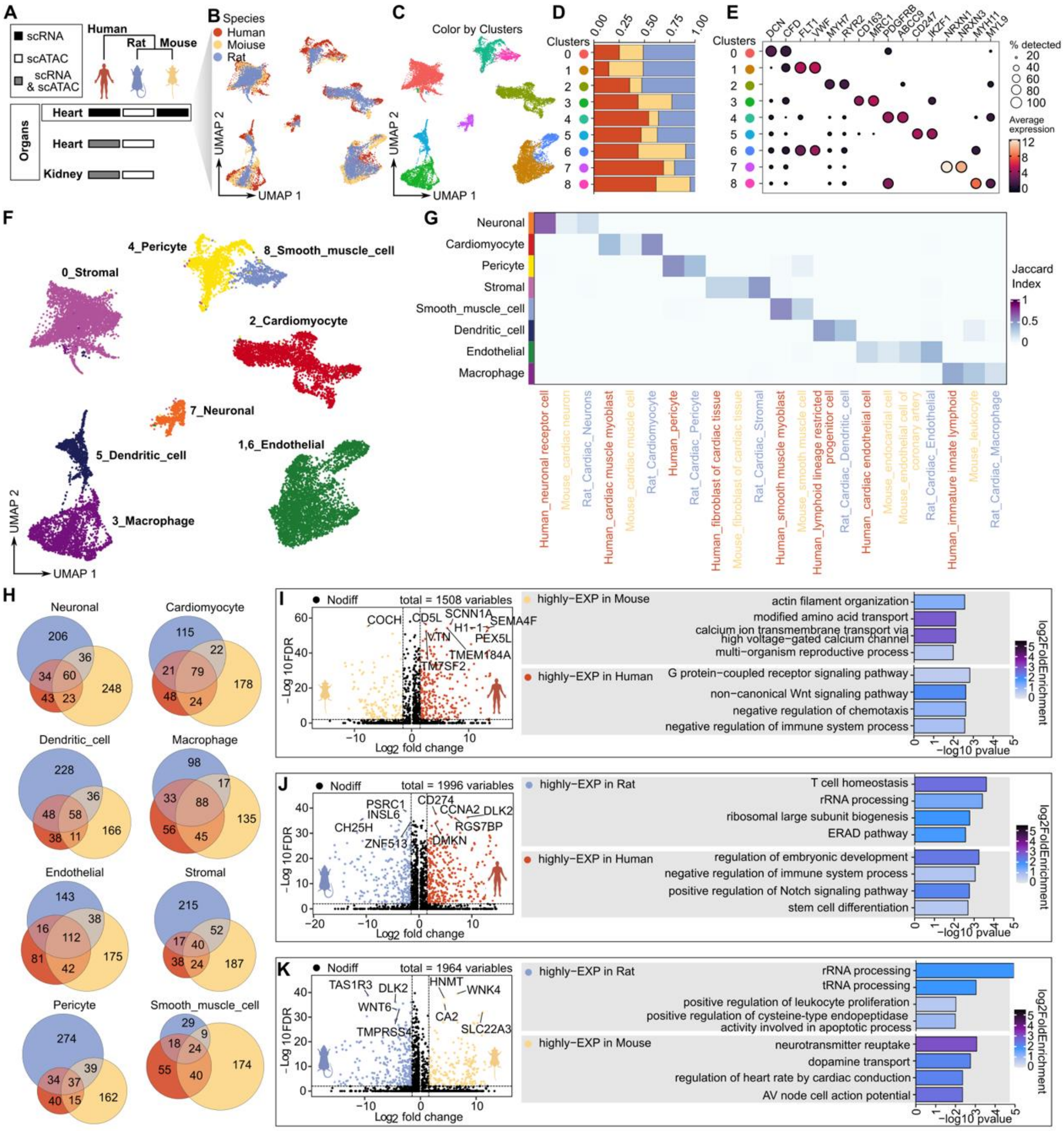

Figure 5. Comparative single-cell transcriptomic and epigenomic analysis across species. **A.** Overview of the types of data (scRNA-seq and scATAC-seq) collected from heart and kidney tissues across humans, rats, and mice. **B.** UMAP plot showing species-specific clustering. **C.** UMAP plot showing the clustering of single cells in the integrated dataset, coloured according to cluster. **D**. Stacked bar plot showing the fraction of each cluster across species (human, mouse, and rat). The different colours represent the different species, with the same colour scheme as in B. **E**. Dot plot displaying the gene expression levels of known marker genes of heart tissue across distinct clusters. The size of the dot corresponds to the percentage of cells expressing the genes in each cluster, and the colour represents the average expression level. **F.** UMAP plot showing the cell types of the integration dataset, coloured by cell type, from the manual annotation of the data in E.**G.** Heatmap showing the similarity (Jaccard index) of cell types across different species. The rows represent the manually annotated cell type labels in the cross-species integration dataset, and the columns represent the cell type label with which the cell was annotated in the original dataset. **H.** Venn

diagrams showing the overlap of differentially expressed genes between species for specific cell types. **I**. Volcano plot showing the results of differential gene expression analysis between human and mouse cardiomyocytes. On the right side, the bar plot shows the top biological processes that are significantly enriched among the DEGs between species. **J**. Same as I but for the results of DEG analysis between human and rat cardiomyocytes. **K**. Same as I but for the results of DEG analysis between mouse and rat cardiomyocytes.

To further examine the species specificity of gene expression patterns, we concentrated our attention on cardiomyocytes, which exhibit relatively high conservation across species (STAR Methods). In general, genes that are more highly expressed in mice (such as *COCH*) are enriched in biological processes such as actin filament organization, amino acid transport, and calcium ion transmembrane transport. On the other hand, genes that are more highly expressed in humans (such as *CD5L*, *SCNN1A*, and *CD274*) are associated with pathways such as G protein-coupled receptor signalling, Wnt signalling, and immune system regulation (Figure 5I). Similarly, for the rat dataset, genes such as *PSRC1* and *DLK2* are associated with biological processes including T-cell homeostasis and leukocyte proliferation (Figure 5J and 5K). These cross-species comparisons underscore specific regulatory pathways that are more active in one species than in the other, illustrating the complexity of gene expression regulation across evolutionary contexts.

In summary, our results indicate that gene scores derived from chromatin accessibility data can serve as useful proxies for gene expression in cross-species data integration analyses, allowing for the comparative assessment of biologically relevant features across species. Using this approach, we examined the conservation and divergence of cell types and gene expression profiles in human, mouse, and rat hearts.

While these results provide an initial resource for studying cross-species differences in heart biology, further analyses incorporating additional data types and functional validation will be required to fully elucidate species-specific gene regulatory mechanisms.

Discussion

The central dogma of molecular biology is that gene expression commences with the transcription of a DNA sequence, and chromatin accessibility has been identified as an important prerequisite for the regulation of gene expression. In recent years, single-cell analysis of transposase-accessible chromatin sequencing (scATAC-seq) has emerged as a powerful tool for elucidating regulatory patterns and cellular heterogeneity. Numerous exciting single-cell chromatin accessibility profiles have been generated via this tool, providing a robust foundation for the analysis of gene regulatory programs across tissues, developmental stages, and cell types within species[24,29–31]. However, the absence of a systematic single-cell chromatin accessibility landscape for the rat (*Rattus norvegicus*), an experimental organism frequently employed in psychological, pharmacological, and behavioural studies, motivated us to generate a single-cell chromatin accessibility landscape across organs.

In this study, we used MGI DNBelab C4 scATAC-seq to create a single-cell dataset of chromatin accessibility from nine organs in the adult rat. This dataset of chromatin accessibility offers new insights into cellular regulation, uncovering gene activity in specific cell types and allowing systematic studies of cell types across various organs. The dataset comprises over 110,000 cells captured from nine organs,

with 77 identified cell types and hundreds of thousands of open chromatin regions. Additionally, we characterize cell type-specific and organ-specific TFs (Data S6), offering detailed insights into the regulatory landscapes of various cell types and organs. This provides a valuable framework for exploring both normal biological processes and disease states.

Despite the similarity in chromatin accessibility observed between endothelial and stromal cells when examining the heterogeneity of cell types across different organs, we continued to assign distinct cell labels according to the organ of origin. This aspect is often overlooked in single-cell atlases from a single organ and, in our view, impedes cross-atlas comparisons. As single-cell technology has developed and cross-organ single-cell atlases have become more widely used, we anticipate that a consensus will be reached within the field to create a more standardized and uniform cell nomenclature. The approach adopted in this study, which entailed dividing the organ dataset for cell annotation and subsequently integrating cell labels from disparate organs, proved more conducive to precise cell type definition. This was evidenced by the observation that immune cells from different organs tended to exhibit similar characteristics and cluster together while retaining some organ specificity. This has the effect of blurring the boundaries between cell types and increasing the difficulty of cell type definition.

Cell fate and state are driven by specific transcription factor regulatory networks. Each cell type has a set of “core TFs” that drive and maintain the transcriptional program of that cell type. For instance, endothelial cells consistently show enrichment for ETS family motifs (e.g., *Elf1*, *Erg*, *Fli1*), cardiomyocytes consistently exhibit motif

enrichment for *Mef2a*, *Mef2d*, and *Mef2b*, and proximal tubule cells are characterized by the enrichment of *Hnf4g*, *Hoxb4*, and *Hoxc4* motifs. We established a chromatin-accessible cellular map across organs in adult rats and were able to query TF motif enrichment at different resolutions (cell subtype, major cell type and organ). As a resource provides a framework for identifying shared and organ-specific regulatory programs, facilitating future efforts in cross-organ cell type annotation, comparison, and discovery. In this study, endothelial and stromal cells were used as examples to illustrate how the same cell type can exhibit both shared and organ-specific molecular signatures across different organs. These differences are shaped by the local microenvironment and functional demands of each organ, which in turn drive distinct chromatin accessibility landscapes and regulatory programs.

There are several limitations of this work that need to be considered. First, all data presented in this study were derived from a single female adult rat at a single time point (7–8 months of age), and all organs were sampled from the same individual. This may limit the ability to capture the full range of biological diversity, including sex-specific, age-dependent, and inter-individual variation. Future studies should include both male and female rats, multiple developmental stages, and more biological replicates to ensure broader applicability and to capture rare or transient cell populations. To increase the technical stability and reproducibility of the experimental data, multiple technical replicates were conducted for each organ. Second, while entire organs were snap-frozen and homogenized for single-nucleus preparation, no sub-regional anatomical dissection was performed. As a result, the dataset represents organ-wide cellular composition and

may not fully capture region-specific cell types within each organ. Third, in cross-species studies, our analyses concentrated on homologous genes and shared cell types, which may have resulted in the omission of some species-specific gene regulatory and expression patterns. We acknowledge these limitations and have sought to clearly outline them to promote scientific rigor and transparency. We hope that by openly addressing these constraints, our work will serve as a robust and reliable resource for the community and a solid foundation for future studies.

In summary, we constructed a single-nucleus multiple-organ chromatin accessibility landscape in the adult rat, which will serve as a valuable resource for investigating gene regulation and cellular diversity across multiple organs. Future studies incorporating greater cell numbers, multiple developmental stages, ages, sexes, spatial information, or experimental conditions will be needed to fully explore dynamic changes in cell states and lineages in the rat.

STAR★Methods

Key resources table

REAGENT or RESOURCE	SOURCE	IDENTIFIER
**Biological samples**		
Sprague-Dawley (SD) rat, Female, 7-8 months, healthy	Jiangsu Ailingfei Biotechnology Company Limited	N/A
**Chemicals, peptides, and recombinant proteins**		
Tris-HCI, pH 8.0	Thermo Fisher Scientific	Cat# 15568025
Sucrose	BBI	Cat# SB0498
KCl	Thermo Fisher Scientific	Cat# AM9640G
MgCl2	MILLIPORE	Cat# 20-303
NP-40	ROCHE	Cat# 11332473001
Protease Inhibitor Cocktail Tablets in EASYpacks	ROCHE	Cat# 4693116001
BSA	BBI	Cat# A600332-0005
DTT	SIGMA	Cat# 646563
PBS PH 7.4	GIBCO	Cat# 10010-031
DAPI Staining Solution	beyotime	Cat# C1006
**Critical commercial assays**		
DNBelab C Series Single-Cell ATAC Library Prep Set	MGI	Cat# 1000021878
Deposited data		
All raw scATAC-seq data	This paper	CNGBdb: CNP0006032
**Software and algorithms**		
R (v4.3.0)	R Core Team	https://www.R-project.org
PISA (v1.2) R package	Shi et al., 2022[32]	https://github.com/shiquan/PISA
Cutadapt (v3.5) R package	Kechin et al., 2017[33]	https://github.com/marcelm/cutadapt
BWA (v0.7.17) R package	Li et al., 2009[34]	https://github.com/lh3/bwa
d2c (v1.4.0) R package	MGI	https://github.com/STOmics/d2c
ArchR (v1.0.2) R package	Granja et al., 2021[35]	https://github.com/GreenleafLab/ArchR

Seurat (v4.4.0) R package	Stuart et al., 2019[14]	https://github.com/satijalab/seurat
ggplot2 (v3.4.2) R package	Wickham et al., 2016[36]	https://github.com/tidyverse/ggplot2/
dplyr (v1.1.4) R package	Wickham et al., 2023[37]	https://github.com/tidyverse/dplyr/
chromVARmotifs (v0.2.0) R package	Schep et al., 2017[38]	https://github.com/GreenleafLab/chromVARmotifs
ComplexHeatmap (v2.18.0) R package	Gu 2022[39]	https://github.com/jokergoo/ComplexHeatmap
BSgenome.Rnorvegicus.UCSC.rn6 (v1.4.1) R package	The Bioconductor Dev Team 2014[40]	https://www.bioconductor.org/packages/release/data/annotation/html/BSgenome.Rnorvegicus.UCSC.rn6.html
TxDb.Rnorvegicus.UCSC.rn6.refGene (v3.4.6) R package	Bioconductor Core Team, Bioconductor Package Maintainer 2019[41]	https://www.bioconductor.org/packages/release/data/annotation/html/TxDb.Rnorvegicus.UCSC.rn6.refGene.html
ensembldb (v2.26.0) R package	Rainer et al., 2019[42]	https://github.com/jorainer/ensembldb
org.Rn.eg.db (v3.18.0) R package	Carlson M 2019[43]	https://www.bioconductor.org/packages/release/data/annotation/html/org.Rn.eg.db.html
scales (v1.3.0) R package	Wickham et al., 2023[44]	https://github.com/r-lib/scales/
SeuratDisk (v0.0.0.9021) R package	Hoffman et al., 2023[45]	https://github.com/mojaveazure/seurat-disk
TFBSTools (v1.40.0) R package	Tan et al., 2016[46]	https://github.com/ge11232002/TFBSTools
biomaRt (v2.58.0) R package	Durinck et al., 2009[47]	https://github.com/grimbough/biomaRt

glmGamPoi (v1.14.3) R package	Ahlmann-Eltze et al., 2021[48]	https://github.com/const-ae/glmGamPoi
eulerr (v7.0.2) R package	Larsson J 2024[49]	https://github.com/jolars/eulerr/
limma (v3.58.1) R package	Ritchie et al., 2015[50]	https://github.com/gangwug/limma
EnhancedVolcano (v1.20.0) R package	Kevin Blighe et al., 2023[51]	https://github.com/kevinblighe/EnhancedVolcano
topGO (v2.54.0) R package	Adrian Alexa et al., 2023[52]	https://github.com/ycl6/topGO-feat
org.Hs.eg.db (v3.19.1) R package	Marc Carlson 2024[53]	https://bioconductor.org/packages/release/data/annotation/html/org.Hs.eg.db.html
stringr (v1.5.1) R package	Hadley Wickham 2023[54]	https://github.com/tidyverse/stringr
Python (v3.10.14)	Python Software Foundation	https://www.python.org/
scanpy (v1.10.2)	Wolf et al., 2018[55]	https://github.com/scverse/scanpy
Other		
Mouse_Thymus_scRNA_dataset	Almanzar et al., 2020[56]	https://datasets.cellxgene.cziscience.com/f4c7c450-16da-4279-a67c-ae6da7e68f958.rds
Mouse_Heart_scRNA_dataset	Almanzar et al., 2020[56]	https://datasets.cellxgene.cziscience.com/e6addc4d-7227-4f6e-a7fa-ae43df336e61.rds
Mouse_Lung_scRNA_dataset	Zepp et al., 2021[57]	https://datasets.cellxgene.cziscience.com/8c70bdd3-8701-40d4-b229-5d14bcf9606c.rds

Mouse_Liver_scRNA_dataset	Almanzar et al., 2020[56]	https://datasets.cellxgene.cziscience.com/e344eaed-332d-4a6e-bfe0-3011013fd3de.rds
Mouse_Spleen_scRNA_dataset	Almanzar et al., 2020[56]	https://datasets.cellxgene.cziscience.com/fab59986-b2d9-49c4-9474-84a99a0b6544.rds
Mouse_Kidney_scRNA_dataset	Novella-Rausell et al., 2023[58]	https://datasets.cellxgene.cziscience.com/a8689831-a9b3-4915-9d62-5d85ce926224.rds
Mouse_Pancreas_scRNA_dataset	Almanzar et al., 2020[56]	https://datasets.cellxgene.cziscience.com/103dc57a-0cbc-4434-bf77-e89e70b59b10.rds
Mouse_Ovary_scRNA_dataset	Garcia-Alonso et al., 2022[59]	https://datasets.cellxgene.cziscience.com/387e9976-3c40-4de1-8009-daa62e40d616.rds
Human_heart_scRNA_dataset	Kuppe et al., 2022[60]	https://datasets.cellxgene.cziscience.com/c1f6034b-7973-45e1-85e7-16933d0550bc.rds
Human_heart_scATAC_dataset	Kuppe et al., 2022[60]	https://datasets.cellxgene.cziscience.com/9bc3febe-9043-4781-bb87-b8647c4f2162.rds

Mouse_heart_scRNA_dataset	Almanzar et al., 2020[56]	https://datasets.cellxgene.cziscience.com/e6addc4d-7227-4f6e-a7fa-ae43df336e61.rds
Human_kidney_scRNA_dataset	Wilson et al., 2022[61]	https://datasets.cellxgene.cziscience.com/14e77ad6-38ce-4aad-8385-68b21aff0737.rds
Human_kidney_scATAC_dataset	Wilson et al., 2022[61]	https://datasets.cellxgene.cziscience.com/5cac872a-685b-4038-90ea-56739fc680c0.rds

Method details

Tissue Dissection and Preservation

In this study, one healthy adult female Sprague-Dawley (SD) rat of 7-8 months of age was used. The animal was purchased from Jiangsu Ailingfei Biotechnology Company Limited. The dissection of rats was conducted by the Guangzhou Institute of Biomedicine and Health (GIBH), Chinese Academy of Sciences (CAS), in accordance with established protocols[62]. In summary, after executing the rats by carbon dioxide asphyxiation, we collected a total of nine organs, including thyroid, thymus, lung, heart, liver, spleen, pancreas, kidney, and ovary, and preserved them in liquid nitrogen tanks using cryopreservation tubes. The use of rats in the relevant experimental study was approved by the Institutional Review Board on the Ethics Committee of BGI (Permit No. BGI-IRB 23050-T1).

Single nucleus ATAC sequencing

The single-cell experiment was divided into three steps.

Step one: Preparation of single-cell suspension. The preparation of single cell suspensions was conducted in accordance with the pre-established method[63]. Briefly, each entire frozen organ was cut into small pieces and transferred into a 2 mL KIMBLE Dounce Tissue Grinder (Sigma, #D8938-1SET) containing 2 mL of ice-cold homogenising buffer [20 mM Tris pH 8.0 (Thermo Fisher Scientific), 500 mM sucrose (BBI), 50 mM KCl (Thermo Fisher Scientific), 10 mM $MgCl_2$ (MILLIPORE), 0.1% NP-40 (Roche), 1× protease inhibitor cocktail (Roche), and 1% nuclease-free BSA, and 0.1 mM DTT]. The tissues were homogenised by 15 strokes of the loose Dounce pestle, and the resulting homogenate was filtered through a 70 μM cell strainer (Falcon, # 352350). Subsequently, the filtered homogenate was subjected to 5 strokes of the tight pestle to facilitate the release of nuclei, which were then filtered once more through a 30 μM cell strainer (PLURISELECT, # 43-50030-03) and transferred to a 15 mL centrifuge tube. The filtered lysate was centrifuged at 500 g for 5 min at 4 °C. The pellet was then washed twice with 1 ml of ice-cold blocking buffer (1× PBS supplemented with 1% BSA), followed by another step of centrifugation at 500 g for 5 min at 4°C. Finally, the nuclei were resuspended in 50 μL of 1× PBS containing 1% BSA and counted with DAPI.

Step two: Construction of libraries. Single-nucleus ATAC-seq libraries were prepared using the DNBelab C Series Single-Cell ATAC Library Prep Set (MGI, #1000021878)[64]. Briefly, Chromatin-open regions were indexing in situ within the nucleus using Tn5 enzyme, after which the labelled nuclei are loaded into a DNBelab C4 microfluidic device for droplet encapsulation. This process is based on the principle of generating nanodroplets through the flow of two immiscible fluids (oil and water) within a microchannel. The droplets serve as discrete reaction chambers, each containing an individual cell nucleus and the requisite biochemical reagents[65]. Subsequently, the process entails PCR pre-amplification, emulsion breaking, bead collection, DNA amplification, and purification. In summary, we generated 25 single-cell ATAC libraries, with at least two technical replicates for each tissue sample.

Step three: Sequencing and alignment. All libraries were sequenced using the bipartite 50 sequencing protocol on the BGISEQ-500 and BGISEQ- T1 platform of the National Genebank of China (CNGB), with a minimum depth of 50,000 reads per nucleus for the libraries. Raw sequencing reads were demultiplexed using PISA[32], adapters were removed using Cutadapt[33], aligned to the rat genome (Rnor_6.0) using BWA[34], and beads were called and merged using d2c(https://github.com/STOmics/d2c). The fragment file generated from each snATAC-seq library served as the basis for downstream analysis.

Creating a custom archRGenome for Rat

In this study, the analysis of single-cell ATAC data was mainly conducted using the ArchR[35] (v.1.0.2). The genome annotation was created using the *createGenomeAnnotation* function, specifying the rat genome as "BSgenome.Rnorvegicus.UCSC.rn6". The gene annotation was generated with *createGeneAnnotation* function, using the TxDb (TxDb.Rnorvegicus.UCSC.rn6.refGene) and OrgDb (org.Rn.eg.db) objects to extract gene-related data, such as TSS, exons, and genes. Finally, the created genome and gene annotations were saved to an RData file. It should be noted that the custom ArchRGenome need to matche the reference genome used to generate the fragments file, which is crucial to avoid errors, such as issues in recognizing transcription start sites (TSS) when creating ArrowFiles.

Preprocessing

The data preprocessing primarily involved three key steps:

First, the removal of low-quality nuclei. We used the *createArrowFiles* function to generate Arrow files from the fragment data. The data were filtered to exclude cells with fewer than 1000 unique nuclear fragments per cell or fewer than 4 TSS enrichment score per cell, as these metrics are crucial for ensuring that only nuclei with adequate chromatin accessibility and transcriptional activity are retained. During the quality control process, it was observed that many cells present in the thyroid samples exhibited a TSS value below 4, which typically indicates these cells are likely dead or dying, as their nucleosomes have begun to unravel. This unraveling can lead to random

transposition events across the entire genome. Despite these cells having high levels of fragmentation (indicating potential chromatin activity), we were still removed from the analysis because they were classified as low-quality cells due to the low TSS enrichment score. This is notwithstanding the possibility that this could occur in certain biological states, such as dormant cells or specific cell types that naturally exhibit low levels of gene expression variability. Nevertheless, we were confident that we had taken the requisite precautions in sample processing. To guarantee the quality and accuracy of subsequent analyses, we retained the cells with high TSS enrichment.

Second, the elimination of potential doublets. we applied the *addDoubletScores* function to infer potential doublets, with the k parameter set to 10 to determine the number of nearest neighbors considered in the doublet detection process. Subsequently, we applied the *filterDoublets* function to remove doublets, with the filterRatio parameter set to 1. The filterRatio parameter controls the stringency of doublet removal; a higher filterRatio results in more cells potentially being identified and removed as doublets. For example, with a dataset of 5000 cells, the maximum number of cells that could be removed as doublets is computed as filterRatio * 5000^2 / 100000, which can be simplified to filterRatio * 5000 * 0.05.

Third, the exclusion of low-quality cell clusters. To enhances the accuracy of quality control, we used *subsetArchRProject* function to divide the entire dataset by organ for preprocessing. We used a for loop in R to perform same operations (like dimensionality reduction, clustering, visualization, marker gene analysis and heatmap generation) individually for each organ. For the dimensionality reduction and clustering, we employed *addIterativeLSI* and *addClusters* functions in ArchR, setting the parameters as follows: iterations at 3, resolution at c(0.2,0.4), varFeatures at 25,000, dimensions ranging from 1 to 30, and a resolution of 0.2 for clustering. For the visualization, we employed *addUMAP* in ArchR, setting the parameters as follows: nNeighbors at 60 and minDistat at 0.6. For the marker gene analysis, we employed *getMarkerFeatures* and *getMarkers* functions in ArchR, setting the parameters as follows: useMatrix at GeneScoreMatrix, groupBy at Clusters, testMethod at Wilcoxon and cutoff at FDR <= 0.01 & Log2FC >= 1. For the heatmap generation, we employed

plotMarkerHeatmap functions in ArchR, setting the parameters as follows: cutoff at FDR <= 0.01 & Log2FC >= 1. During the viewing of the UMAP plot with Gene Scores Marker Heatmap, we manually identified low-quality clusters and removed them from the dataset based on the following rules:

a. The cluster did not express distinctly specific genes.

b. The number of cells of cluster less than 50.

c. The same cell cluster simultaneously expresses marker genes typical of multiple cell types and has a high doublet score.

After removal of low-quality cell clusters， we again performed the same operation as described above with the same parameters individually for each organ until the final clustering results in composite quality requirements.

Annotation

To manually identify organ-specific cell types and states, we annotated cells within each organ dataset separately before integration, we employed *plotEmbedding* and *plotGroups* functions to visualize known marker genes of cell types individually for each organ, setting the parameters as follows: colorBy at GeneScoreMatrix, groupBy at Clusters. The list of known marker genes of cell types utilized in this study was derived from the aggregation of data from our previous investigation. Each cluster was meticulously annotated based on the established practices and insights provided by previous researchers[66].

a. If a cluster expresses less than 3 markers related to a specific cell type with low expression, it is judged that the cluster does not belong to that cell type.

b. If multiple clusters co-express more than 3 markers related to the same cell type with high expression, it is judged that these clusters all belong to the same cell type.

c. If a cluster expresses multiple markers of different cell types and the first 10 marker genes of different cell types significantly mark the same cluster, the cluster is judged to be doublet and removed.

Additionally, we comprehensively considered the highly expressed gene profiles of each cluster while identifying each cell type. For the same cluster that unambiguously expresses marker genes of two cell types separately by contour region,

the value of resolution in the *addClusters* function was appropriately increased to more accurately identify the cell type. We recommend trying multiple parameters and observing the cluster divisions when performing dimensionality reduction and cluster to balance the clustering granularity and biological interpretability.

Label transfer

To help with cluster identity assignment, we used *addGeneIntegrationMatrix* function in ArchR to directly align cells from scATAC-seq with cells from scRNA-seq by comparing the scATAC-seq gene score matrix with the scRNA-seq gene expression matrix. This function converts the gene score matrix from the ArchR project into a Seurat object and uses *FindTransferAnchors* function from the Seurat[14] package which allows you to perform CCA-based integration between the scATAC-seq data and the scRNA-seq data.

To compare predicted cell types from scRNA datasets with manually annotated cell types in scATAC datasets, we created a confusion matrix using the *confusionMatrix* function, calculated the similarity between the two sets of labels using the *jaccardIndex* function, added row and column annotations with the *HeatmapAnnotation* function, and then plotted the heatmap with customized aesthetics using the *heatmap* function.

Considering the current dearth of rat cross-organ single-cell RNA datasets, we employed data derived from mouse for integration in the present study. The download link for the mouse scRNA datasets used are as follows:

Thymus: https://datasets.cellxgene.cziscience.com/f4c7c450-16da-4279-a67c-a6da7e68f958.rds

Heart: https://datasets.cellxgene.cziscience.com/e6addc4d-7227-4f6e-a7fa-ae43df336e61.rds

Lung: https://datasets.cellxgene.cziscience.com/8c70bdd3-8701-40d4-b229-5d14bcf9606c.rds

Liver: https://datasets.cellxgene.cziscience.com/e344eaed-332d-4a6e-bfe0-3011013fd3de.rds

Spleen: https://datasets.cellxgene.cziscience.com/fab59986-b2d9-49c4-9474-84a99a0b6544.rds

Kidney: https://datasets.cellxgene.cziscience.com/a8689831-a9b3-4915-9d62-5d85ce926224.rds

Pancreas: https://datasets.cellxgene.cziscience.com/103dc57a-0cbc-4434-bf77-e89e70b59b10.rds

Ovary: https://datasets.cellxgene.cziscience.com/387e9976-3c40-4de1-8009-daa62e40d616.rds

All the above datasets were obtained from the online website CZ CELLxGENE: Discover[67,68], with thanks to them for providing free access to convenient, standardized scRNA dataset downloads to facilitate the exploration and sharing of single cell datasets.

Subcluster label assignment to full project

As described above, after identifying organ-specific cell types and low-quality clusters in each organ separately, we mapped these label matches back to the entire dataset for further analysis. For redoing dimensionality reduction and clustering, we used *addIterativeLSI* and *addClusters* functions, setting the parameters as follows: iterations at 2, resolution at 0.6, varFeatures at 25,000, dimensions ranging from 1 to 30, and a resolution of 0.2 for clustering.

To visualize the expression of marker genes for each cell type in each organ, we employed the *dotplot* function in Scanpy[55]. We first need to convert the GeneScoreMatrix from ArchR project into a Seurat object. We got the gene score matrix from ArchR project using the *getMatrixFromProject* function and created Seurat object using the *CreateAssayObject* and *CreateSeuratObject* function. The Seurat object was normalized (LogNormalize method) and variable features were identified (vst method with 2000 features) using *NormalizeData* and *FindVariableFeatures* functions. It was then saved in h5Seurat format using *SaveH5Seurat* function and converted into an h5ad format using *Convert* function, which is compatible with AnnData, often used in Python for further single-cell RNA-seq analysis. Additionally, the metadata from the ArchR project was extracted using *getCellColData* function and saved as a CSV file. In the Scanpy analysis, we used the default parameters to identify differentially expressed genes between cell subtypes and then filtered these results by

a minimum fold change of 1. We subset each organ data to generated the expression of marker genes using *sc.pl.dotplot* function.

To compute hierarchical clustering of cell subtypes, we employed *sc.tl.dendrogram* functions with using “complete” linkage and optimal ordering to understand the hierarchical relationships between different cell subtypes.

Peaks calling

We created pseudo-bulk replicates, a bulk ATAC-seq experiment, allowing for more robust downstream analyses by reducing noise and enabling statistical comparisons, based on major cell types in the dataset using *addGroupCoverages* function in ArchR. To call peaks using MACS2[69], we utilized *addReproduciblePeakSet* function to generate reproducible peak set across cells grouped by major cell types in ArchR.

Motif Enrichments

To determine which transcription factors (proteins that bind to specific DNA sequences to regulate gene expression) are responsible for binding events, we utilized *addMotifAnnotations* function to add motif information to the ArchR project, setting the parameters as follows: motifSet at cisbp[70], species at mus musculus. Although rat-specific motifs are ideal, the limited availability justifies the use of mouse motifs. Many TF binding sites are conserved across closely related species, so using mouse motifs can still provide meaningful insights.

For the motif enrichments analysis, we employed *getMarkerFeatures*, *peakAnnoEnrichment* and *plotEnrichHeatmap* functions in ArchR, setting the parameters as follows: useMatrix at PeakMatrix, groupBy at cell subtypes or organs, testMethod at wilcoxon and cutoff at FDR <= 0.1 & Log2FC >= 0.5.

For identifying and visualizing the most enriched TFs associated with chromatin accessibility in different major cell types, we identified marker genes (via GeneScoreMatrix) and motif enrichments (via PeakMatrix) across major cell types and merge gene scores with motif enrichment data, linking TF activity to specific gene expression patterns.

Analysis endothelial and stromal cell across organs

To identify and analysis of endothelial and stromal cell diversity and regulatory elements across organs, we first extracted endothelial and stromal cell from the full dataset and recalled specific peaks for cell subtypes (consistent as described in the previous methods but grouped by cell subtype). Dimensionality reduction was then performed using *addIterativeLSI* function, setting the parameters as follows: iterations at 2, resolution at 2.0, varFeatures at 25,000, useMatrix at PeakMatrix, anddimensions ranging from 1 to 35, and a resolution of 0.2 for clustering. Visualised in low-dimensional space using *addUMAP* function with nNeighbors at 40 and minDist at 0.4.

To investigate the molecular heterogeneity of the same cell type across different organs by identifying both organ-specific and conserved gene signatures, we extracted GeneScoreMatrix from the ArchR object using the *getMatrixFromProject* function in ArchR and the gene names were assigned as row names. We then converted it into a Seurat assay object using *CreateAssayObject* function and wrapped in a Seurat object using *CreateSeuratObject* function. To convert Seurat object to anndata, we firstly saved Seurat object in the Seurat format (.h5Seurat) using the *SaveH5Seurat* function and then converted it to the annData format (.h5ad) using the *Convert* function from the SeuratDisk package (v0.0.0.90).

Differential expression analysis was performed using Scanpy's rank_genes_groups() with Wilcoxon rank-sum test across organs. Significantly upregulated genes ($log2FC \geq 1$, $FDR < 0.01$) were identified per organ, and top markers were visualized. Conserved marker genes were defined as those differentially expressed in at least four organs in endothelial cells dataset or seven organs in stromal cells dataset and ranked by average z-score-normalized expression across organs. Functional enrichment analysis using gProfiler was then performed separately for conserved and organ-specific marker genes.

To ensure robustness in cross-organ motif enrichment analysis, cell populations with fewer than 500 cells were excluded, as small cell counts can lead to unreliable peak calling and inflated false positives in downstream enrichment analyses. To identify and visualize TF motif enrichment across different organs based on chromatin accessibility data, differentially accessible peaks were first identified using

getMarkerFeatures with a Wilcoxon test, accounting for TSS enrichment and fragment count biases. These peak sets were then used for motif enrichment analysis via peakAnnoEnrichment, with significant motifs defined by FDR ≤ 0.01 and log2FC ≥ 1.

Cross species integration

The process of cross-species data integration can be divided into three steps.

Step one: Data preprocessing. For the rat dataset, we extracted the GeneScoreMatrix from an ArchR project using *getMatrixFromProject* function and converted it into a Seurat object using *CreateAssayObject* and *CreateSeuratObject* functions. To ensure consistency of gene names across species datasets, we converted gene symbols in the Seurat object to Ensembl IDs using the *bitr* function from the clusterProfiler[71] package, leveraging the org.Rn.eg.db database. To ensure comparability of cell types in cross-species data integration and comparative analyses, we screened for homologous cell types across species and down-sampled according to cell type to ensure that the number of cells of each cell type in the analyses is in a reasonable range (e.g., a minimum of 50 and a maximum of 1000).

Step two: Homologous substitution of gene names. We connected to the Ensembl database using the biomaRt[47] package and converted rat and mouse gene symbols to their human homologs.

Step three: Cross species integration. We began by compiling the data into a list of Seurat objects. To normalize and standardize the data, we applied the *SCTransform* function with the glmGamPoi method to each dataset in the list, ensuring that all variable genes were retained (return.only.var.genes = F). Next, we selected 3,000 integration features across the datasets using the *SelectIntegrationFeatures* function, which identifies the most consistent and variable genes for integration. We then prepared the data for integration using *PrepSCTIntegration* and identified anchors across the datasets with *FindIntegrationAnchors*, using the selected features and the first 30 principal components (dims = 1:30) to align the datasets. We integrated the data using the *IntegrateData* function, which combined the datasets into a single Seurat object normalized with the SCT method. After integration, we reduced the dimensionality of the data using PCA with *RunPCA*, and then visualized it in a lower-

dimensional space using UMAP with *RunUMAP*. Finally, we identified cell clusters by *FindNeighbors* and clustering them with the Louvain algorithm (*FindClusters*), setting the resolution to 0.3 to control the cluster size. To identify the shared and unique DEGs among human, mouse, and rat, we subsetted into separate datasets for Human, Mouse, and Rat and down sampled each dataset to 200 cells per cell type to ensure comparable cell numbers across species. we then identified DEGs for each cell type within each species using the *FindAllMarkers* function with default parameters. To visualize represent the overlap of DEGs among Human, Mouse, and Rat, we generated Venn diagram using the eulerr package (https://github.com/jolars/eulerr). To perform differential expression analysis across species (Human vs. Mouse, Human vs. Rat, Mouse vs. Rat), we ued the *FindMarkers* function with default parameter. Volcano plots was generated using the EnhancedVolcano package (https://github.com/kevinblighe/EnhancedVolcano). Gene Ontology (GO) enrichment analysis was performed using topGO package (https://bioconductor.org/packages/release/bioc/vignettes/topGO/inst/doc/topGO.pdf).

The download link for the human or mouse scRNA/scATAC datasets used for Cross species integration are as follows:

Human_heart_scRNA-seq:

https://datasets.cellxgene.cziscience.com/c1f6034b-7973-45e1-85e7-16933d0550bc.rds

Human_heart_scATAC-seq:

https://datasets.cellxgene.cziscience.com/9bc3febe-9043-4781-bb87-b8647c4f2162.rds

Mouse_heart_scRNA-seq:

https://datasets.cellxgene.cziscience.com/e6addc4d-7227-4f6e-a7fa-ae43df336e61.rds

Human_kidney_scRNA-seq:

https://datasets.cellxgene.cziscience.com/14e77ad6-38ce-4aad-8385-68b21aff0737.rds

Human_kidney_scATAC-seq:

https://datasets.cellxgene.cziscience.com/5cac872a-685b-4038-90ea-56739fc680c0.rds

All the above datasets were obtained from the online website CZ CELLxGENE: Discover, with thanks to them for providing free access to convenient, standardized scRNA dataset downloads to facilitate the exploration and sharing of single cell datasets.

Data availability

The data supporting the findings of this study have been deposited into CNGB Sequence Archive (CNSA)[72] of China National GeneBank DataBase (CNGBdb)[73] with accession number CNP0006032.

Code availability

We have described the process of analyzing the data for this study in detail in the methods section. If you have questions about data analysis or are looking for the full code, please contact by mail at liuchuanyu@genomics.cn.

Acknowledgements

We are especially grateful to Dr. Duoyuan Chen, Dr. Xi Dai and Dr. Shijie Hao of BGI Research for their helpful comments in cross-species analysis. This work was supported by the Shenzhen Key Laboratory of Single-Cell Omics (ZDSYS20190902093613831).

Author contributions

Y.Y. and L.L. designed the project and experiments. Y.Y., S.D. and Q.D. conducted snATAC-seq experiments. W.M. processed the raw sequencing data. R.L. performed the data analysis and wrote the manuscript. C.L. participated in the supervision of this research. R.L., Y.Y., C.L., L.L. and P.G. revised the manuscript. All authors have read and approved the final manuscript. The remaining authors declare no competing interests.

Declaration of interests

The authors declare no competing interests.

Supplemental information

Document S1. Figures S1–S6

Table S1. The main marker genes were used for annotation in this paper, related to Figure S1 and S2

Data S1. The metadata of dataset in this paper, related to Figure 1 and S1

Data S2. The data frame that contains the UMAP coordinates for each cell in the ArchR project, related to Figure 1

Data S3. The data frame contains information about top20 genes identified for each cell subtype, related to Figure 2

Data S4. The data frame contains the results of specific TF binding motifs are significantly enriched in the accessible chromatin regions (peaks) associated with different cell subtypes, related to Figure 3

Data S5. The data frame contains the results of specific TF binding motifs are significantly enriched in the accessible chromatin regions (peaks) associated with different major cell types, related to Figure 3

Data S6. The data frame contains the results of specific TF binding motifs are significantly enriched in the accessible chromatin regions (peaks) associated with different organs, related to Figure 3

Data S7. The data frame contains the results of GO term enrichment of organ-specific gene scores across multiple organs in endothelial cells, related to Figure 4

Data S8. The data frame contains the results of GO term enrichment of organ-specific gene scores across multiple organs in stromal cells, related to Figure 4

Data S9. A comprehensive dataset that consolidates differential expression analysis results across multiple subclasses and species., related to Figure 5

References

1. Regev A, Teichmann SA, Lander ES, et al. The Human Cell Atlas. *Elife*. 2017;6. doi:10.7554/eLife.27041
2. Schafflick D, Wolbert J, Heming M, et al. Single-cell profiling of CNS border compartment leukocytes reveals that B cells and their progenitors reside in non-diseased meninges. *Nat Neurosci*. 2021;24(9):1225-1234. doi:10.1038/s41593-021-00880-y
3. Mays JC, Kelly MC, Coon SL, et al. Single-cell RNA sequencing of the mammalian pineal gland identifies two pinealocyte subtypes and cell type-specific daily patterns of gene expression. *PLoS One*. 2018;13(10):e0205883. doi:10.1371/journal.pone.0205883
4. Ding F, Tian X, Mo J, Wang B, Zheng J. Determination of the dynamic cellular transcriptional profiles during kidney development from birth to maturity in rats by single-cell RNA sequencing. *Cell Death Discov*. 2021;7(1):162. doi:10.1038/s41420-021-00542-9
5. Guan X, Ji M, Wen X, et al. Single-cell RNA sequencing of adult rat testes after Leydig cell elimination and restoration. *Sci Data*. 2022;9(1):106. doi:10.1038/s41597-022-01225-5
6. Ma S, Sun S, Geng L, et al. Caloric Restriction Reprograms the Single-Cell Transcriptional Landscape of Rattus Norvegicus Aging. *Cell*. 2020;180(5):984-1001.e22. doi:10.1016/j.cell.2020.02.008
7. Raredon MSB, Adams TS, Suhail Y, et al. Single-cell connectomic analysis of adult mammalian lungs. *Sci Adv*. 2019;5(12):eaaw3851. doi:10.1126/sciadv.aaw3851
8. Yu Y, Wei X, Deng Q, et al. Single-Nucleus Chromatin Accessibility Landscape Reveals Diversity in Regulatory Regions Across Distinct Adult Rat Cortex. *Front Mol Neurosci*. 2021;14:651355. doi:10.3389/fnmol.2021.651355
9. Ma P, Duan S, Ma W, et al. Single-cell chromatin accessibility landscape profiling reveals the diversity of epigenetic regulation in the rat nervous system. *Sci Data*. 2025;12(1):140. doi:10.1038/s41597-025-04432-y
10. Single-cell transcriptomics of 20 mouse organs creates a Tabula Muris. *Nature*. 2018;562(7727):367-372. doi:10.1038/s41586-018-0590-4
11. Domcke S, Hill AJ, Daza RM, et al. A human cell atlas of fetal chromatin accessibility. *Science (1979)*. 2020;370(6518). doi:10.1126/science.aba7612
12. Ashby KM, Hogquist KA. A guide to thymic selection of T cells. *Nat Rev Immunol*. Published online July 18, 2023. doi:10.1038/s41577-023-00911-8
13. Elmentaite R, Domínguez Conde C, Yang L, Teichmann SA. Single-cell atlases: shared and tissue-specific cell types across human organs. *Nat Rev Genet*. 2022;23(7):395-410. doi:10.1038/s41576-022-00449-w
14. Stuart T, Butler A, Hoffman P, et al. Comprehensive Integration of Single-Cell Data. *Cell*. 2019;177(7):1888-1902.e21. doi:10.1016/j.cell.2019.05.031

15. Lin P, Yan X, Jing S, et al. Single-cell and spatially resolved transcriptomics for liver biology. *Hepatology*. 2024;80(3):698-720. doi:10.1097/HEP.0000000000000387
16. Molitoris BA, Sandoval RM, Yadav SPS, Wagner MC. Albumin uptake and processing by the proximal tubule: physiological, pathological, and therapeutic implications. *Physiol Rev*. 2022;102(4):1625-1667. doi:10.1152/physrev.00014.2021
17. Travaglini KJ, Nabhan AN, Penland L, et al. A molecular cell atlas of the human lung from single-cell RNA sequencing. *Nature*. 2020;587(7835):619-625. doi:10.1038/s41586-020-2922-4
18. Pham TH, Minderjahn J, Schmidl C, et al. Mechanisms of in vivo binding site selection of the hematopoietic master transcription factor PU.1. *Nucleic Acids Res*. 2013;41(13):6391-6402. doi:10.1093/nar/gkt355
19. Le Coz C, Nguyen DN, Su C, et al. Constrained chromatin accessibility in PU.1-mutated agammaglobulinemia patients. *Journal of Experimental Medicine*. 2021;218(7). doi:10.1084/jem.20201750
20. Teng M, Zhou S, Cai C, Lupien M, He HH. Pioneer of prostate cancer: past, present and the future of FOXA1. *Protein Cell*. 2021;12(1):29-38. doi:10.1007/s13238-020-00786-8
21. Stemmler MP, Eccles RL, Brabletz S, Brabletz T. Non-redundant functions of EMT transcription factors. *Nat Cell Biol*. 2019;21(1):102-112. doi:10.1038/s41556-018-0196-y
22. Koyano-Nakagawa N, Garry DJ. Etv2 as an essential regulator of mesodermal lineage development. *Cardiovasc Res*. 2017;113(11):1294-1306. doi:10.1093/cvr/cvx133
23. Suresh H, Crow M, Jorstad N, et al. Comparative single-cell transcriptomic analysis of primate brains highlights human-specific regulatory evolution. *Nat Ecol Evol*. 2023;7(11):1930-1943. doi:10.1038/s41559-023-02186-7
24. Bakken TE, Jorstad NL, Hu Q, et al. Comparative cellular analysis of motor cortex in human, marmoset and mouse. *Nature*. 2021;598(7879):111-119. doi:10.1038/s41586-021-03465-8
25. Hao S, Zhu X, Huang Z, et al. Cross-species single-cell spatial transcriptomic atlases of the cerebellar cortex. *Science (1979)*. 2024;385(6716). doi:10.1126/science.ado3927
26. Song Y, Miao Z, Brazma A, Papatheodorou I. Benchmarking strategies for cross-species integration of single-cell RNA sequencing data. *Nat Commun*. 2023;14(1):6495. doi:10.1038/s41467-023-41855-w
27. Li YE, Preissl S, Miller M, et al. A comparative atlas of single-cell chromatin accessibility in the human brain. *Science (1979)*. 2023;382(6667). doi:10.1126/science.adf7044
28. Litviňuková M, Talavera-López C, Maatz H, et al. Cells of the adult human heart. *Nature*. 2020;588(7838):466-472. doi:10.1038/s41586-020-2797-4

29. Cusanovich DA, Hill AJ, Aghamirzaie D, et al. A Single-Cell Atlas of In Vivo Mammalian Chromatin Accessibility. *Cell*. 2018;174(5):1309-1324.e18. doi:10.1016/j.cell.2018.06.052
30. Zhang K, Hocker JD, Miller M, et al. A single-cell atlas of chromatin accessibility in the human genome. *Cell*. 2021;184(24):5985-6001.e19. doi:10.1016/j.cell.2021.10.024
31. Calderon D, Blecher-Gonen R, Huang X, et al. The continuum of Drosophila embryonic development at single-cell resolution. *Science*. 2022;377(6606):eabn5800. doi:10.1126/science.abn5800
32. Shi Q, Liu S, Kristiansen K, Liu L. The FASTQ+ format and PISA. *Bioinformatics*. 2022;38(19):4639-4642. doi:10.1093/bioinformatics/btac562
33. Kechin A, Boyarskikh U, Kel A, Filipenko M. cutPrimers: A New Tool for Accurate Cutting of Primers from Reads of Targeted Next Generation Sequencing. *J Comput Biol*. 2017;24(11):1138-1143. doi:10.1089/cmb.2017.0096
34. Li H, Durbin R. Fast and accurate short read alignment with Burrows–Wheeler transform. *Bioinformatics*. 2009;25(14):1754-1760. doi:10.1093/bioinformatics/btp324
35. Granja JM, Corces MR, Pierce SE, et al. ArchR is a scalable software package for integrative single-cell chromatin accessibility analysis. *Nat Genet*. 2021;53(3):403-411. doi:10.1038/s41588-021-00790-6
36. Wickham H. *Ggplot2*. Springer International Publishing; 2016. doi:10.1007/978-3-319-24277-4
37. Hadley Wickham, Romain François, Lionel Henry, Kirill Müller, Davis Vaughan. *Dplyr: A Grammar of Data Manipulation*.; 2023.
38. Schep AN, Wu B, Buenrostro JD, Greenleaf WJ. chromVAR: inferring transcription-factor-associated accessibility from single-cell epigenomic data. *Nat Methods*. 2017;14(10):975-978. doi:10.1038/nmeth.4401
39. Gu Z. Complex heatmap visualization. *iMeta*. 2022;1(3). doi:10.1002/imt2.43
40. Team TBD. BSgenome.Rnorvegicus.UCSC.rn6: Full genome sequences for Rattus norvegicus (UCSC version rn6). Published online 2014.
41. Team BC, Maintainer BP. TxDb.Rnorvegicus.UCSC.rn6.refGene: Annotation package for TxDb object(s). Published online 2019.
42. Rainer J, Gatto L, Weichenberger CX. ensembldb: an R package to create and use Ensembl-based annotation resources. *Bioinformatics*. 2019;35(17):3151-3153. doi:10.1093/bioinformatics/btz031
43. Carlson M. org.Rn.eg.db: Genome wide annotation for Rat. Published online 2019.
44. Hadley Wickham, Thomas Lin Pedersen, Dana Seidel. *Scales: Scale Functions for Visualization*.; 2023. doi:https://CRAN.R-project.org/package=scales

45. Paul Hoffman, Rahul Satija. Interfaces for HDF5-Based Single Cell File Formats. Published online 2023. doi:https://github.com/mojaveazure/seurat-disk
46. Tan G, Lenhard B. TFBSTools: an R/bioconductor package for transcription factor binding site analysis. *Bioinformatics*. 2016;32(10):1555-1556. doi:10.1093/bioinformatics/btw024
47. Durinck S, Spellman PT, Birney E, Huber W. Mapping identifiers for the integration of genomic datasets with the R/Bioconductor package biomaRt. *Nat Protoc*. 2009;4(8):1184-1191. doi:10.1038/nprot.2009.97
48. Ahlmann-Eltze C, Huber W. glmGamPoi: fitting Gamma-Poisson generalized linear models on single cell count data. *Bioinformatics*. 2021;36(24):5701-5702. doi:10.1093/bioinformatics/btaa1009
49. Larsson J. eulerr: Area-Proportional Euler and Venn Diagrams with Ellipses. Published online 2024. doi:https://CRAN.R-project.org/package=eulerr
50. Ritchie ME, Phipson B, Wu D, et al. limma powers differential expression analyses for RNA-sequencing and microarray studies. *Nucleic Acids Res*. 2015;43(7):e47-e47. doi:10.1093/nar/gkv007
51. Kevin Blighe, Sharmila Rana, Myles Lewis. EnhancedVolcano: Publication-ready volcano plots with enhanced colouring and labeling. Published online 2023. doi:10.18129/B9.bioc.EnhancedVolcano
52. Adrian Alexa, Jorg Rahnenfuhrer. topGO: Enrichment Analysis for Gene Ontology. Published online 2023. doi:10.18129/B9.bioc.topGO
53. Marc Carlson. org.Hs.eg.db: Genome wide annotation for Human. Published online 2024.
54. Hadley Wickham. stringr: Simple, Consistent Wrappers for Common String Operations. Published online 2023. doi:https://CRAN.R-project.org/package=stringr
55. Wolf FA, Angerer P, Theis FJ. SCANPY: large-scale single-cell gene expression data analysis. *Genome Biol*. 2018;19(1):15. doi:10.1186/s13059-017-1382-0
56. Almanzar N, Antony J, Baghel AS, et al. A single-cell transcriptomic atlas characterizes ageing tissues in the mouse. *Nature*. 2020;583(7817):590-595. doi:10.1038/s41586-020-2496-1
57. Zepp JA, Morley MP, Loebel C, et al. Genomic, epigenomic, and biophysical cues controlling the emergence of the lung alveolus. *Science (1979)*. 2021;371(6534). doi:10.1126/science.abc3172
58. Novella-Rausell C, Grudniewska M, Peters DJM, Mahfouz A. A comprehensive mouse kidney atlas enables rare cell population characterization and robust marker discovery. *iScience*. 2023;26(6):106877. doi:10.1016/j.isci.2023.106877
59. Garcia-Alonso L, Lorenzi V, Mazzeo CI, et al. Single-cell roadmap of human gonadal development. *Nature*. 2022;607(7919):540-547. doi:10.1038/s41586-022-04918-4

60. Kuppe C, Ramirez Flores RO, Li Z, et al. Spatial multi-omic map of human myocardial infarction. *Nature*. 2022;608(7924):766-777. doi:10.1038/s41586-022-05060-x
61. Wilson PC, Muto Y, Wu H, Karihaloo A, Waikar SS, Humphreys BD. Multimodal single cell sequencing implicates chromatin accessibility and genetic background in diabetic kidney disease progression. *Nat Commun*. 2022;13(1):5253. doi:10.1038/s41467-022-32972-z
62. Lőw P, Molnár K, Kriska G. Dissection of the Rat (Rattus norvegicus). In: *Atlas of Animal Anatomy and Histology*. Springer International Publishing; 2016:325-399. doi:10.1007/978-3-319-25172-1_12
63. Yu Y, Wei X, Deng Q, et al. Single-Nucleus Chromatin Accessibility Landscape Reveals Diversity in Regulatory Regions Across Distinct Adult Rat Cortex. *Front Mol Neurosci*. 2021;14:651355. doi:10.3389/fnmol.2021.651355
64. Han L, Wei X, Liu C, et al. Cell transcriptomic atlas of the non-human primate Macaca fascicularis. *Nature*. 2022;604(7907):723-731. doi:10.1038/s41586-022-04587-3
65. Liu C, Wu T, Fan F, et al. A portable and cost-effective microfluidic system for massively parallel single-1 cell transcriptome profiling 2 3. *bioRxiv*. Published online October 25, 2019. doi:10.1101/818450
66. Russ DE, Cross RBP, Li L, et al. A harmonized atlas of mouse spinal cord cell types and their spatial organization. *Nat Commun*. 2021;12(1):5722. doi:10.1038/s41467-021-25125-1
67. Prins L, Badajoz S, Mccandless B, et al. cellxgene: a performant, scalable exploration platform for high dimensional sparse matrices Last authors (direction and supervision). *bioRxiv*. Published online April 5, 2021. doi:10.1101/2021.04.05.438318
68. Abdulla S, Aevermann B, Assis P, et al. CZ CELL×GENE Discover: A single-cell data platform for scalable exploration, analysis and modeling of aggregated data CZI Single-Cell Biology Program. *bioRxiv*. Published online October 30, 2023. doi:10.1101/2023.10.30.563174
69. Zhang Y, Liu T, Meyer CA, et al. Model-based Analysis of ChIP-Seq (MACS). *Genome Biol*. 2008;9(9):R137. doi:10.1186/gb-2008-9-9-r137
70. Ray D, Kazan H, Cook KB, et al. A compendium of RNA-binding motifs for decoding gene regulation. *Nature*. 2013;499(7457):172-177. doi:10.1038/nature12311
71. Xu S, Hu E, Cai Y, et al. Using clusterProfiler to characterize multiomics data. *Nat Protoc*. Published online July 17, 2024. doi:10.1038/s41596-024-01020-z
72. Guo X, Chen F, Gao F, et al. CNSA: a data repository for archiving omics data. *Database*. 2020;2020. doi:10.1093/database/baaa055
73. Chen FZ, You LJ, Yang F, et al. CNGBdb: China National GeneBank DataBase. *Yi Chuan*. 2020;42(8):799-809. doi:10.16288/j.yczz.20-080

Click here to access/download
Supplementary Material
Data S1.xlsx

Click here to access/download
Supplementary Material
Data S2.xlsx

Click here to access/download
Supplementary Material
Data S3.xlsx

Click here to access/download
Supplementary Material
Data S4.xlsx

Click here to access/download
Supplementary Material
Data S5.xlsx

Click here to access/download
Supplementary Material
Data S6.xlsx

Click here to access/download
Supplementary Material
Data S7.xlsx

Click here to access/download
Supplementary Material
Data S8.xlsx

Click here to access/download
Supplementary Material
Data S9.xlsx

Supplementary Figure

Click here to access/download
Supplementary Material
Document S1.docx

Supplementary Table

Click here to access/download
Supplementary Material
Table S1.xlsx

Cover Letter

Click here to access/download;Personal Cover;Cover_Letter.pdf

华大生命科学研究院
BGI·Research

August 12th, 2025

Dear Editor,

Please find enclosed our manuscript titled "**Single-nucleus multiple-organ chromatin accessibility landscape in the adult rat**" by Ronghai Li et al., which we would like you to consider for publication in ***Gigascience***. We believe that our work would be a valuable addition to your journal, as it presents a single-nucleus chromatin accessibility landscape across nine adult rat organs (Rattus norvegicus), which facilitates the exploration of cell type-specific regulatory features and enables cross-organ and cross-species comparisons of gene regulatory programs.

The Human Cell Atlas (HCA) project aims to create a comprehensive reference cell atlas of all cells in the human body (the basic unit of life). This will serve as a basis for understanding human health and diagnosing, monitoring, and treating disease[1]. In recent years, single-cell analysis of transposase-accessible chromatin sequencing (scATAC-seq) has emerged as a powerful tool for elucidating regulatory patterns and cellular heterogeneity. Numerous exciting single-cell chromatin accessibility profiles have been generated via this tool, providing a robust foundation for analyzing gene regulatory programs across tissues, developmental stages, and cell types within species[2–5]. In our previous work, we constructed single-nucleus chromatin accessibility landscape of the rat brain and spinal cord, providing valuable resources for understanding region- and cell type-specific gene regulation in the mammalian nervous system[6,7]. Building upon this foundation, the present study extends our efforts to profile chromatin accessibility across nine major organs in the rat. This multi-organ dataset not only supplements and extends our previous dataset but also provides a valuable resource for the research community, supporting studies of epigenomic diversity and gene regulation across different organs and cell types in the rat.

To address this gap, we used MGI DNBelab C4 scATAC-seq to create a single-cell atlas of chromatin accessibility from nine organs in the adult rat. This atlas of chromatin accessibility offers new insights into cellular regulation, uncovering gene activity in specific cell types and allowing systematic and comparative studies of cell types across various organs. The dataset comprises over 110,000 cells captured from nine organs, with 77 identified cell types and hundreds of thousands of cCREs. Additionally, we characterize cell type-specific and organ-specific TFs, offering detailed insights into the regulatory landscapes of various cell types and organs. The highlights of our research are as follows:

1. **Generation of a single-cell atlas of chromatin accessibility in nine organs of the rat**

Here, we report the cellular composition and chromatin accessibility landscape of multiple organs in the adult rat. The atlas consists of single-cell epigenomic data from 115,723 nuclei isolated from nine organs (namely the thyroid, thymus, heart, lung, liver, spleen, kidney, pancreas, and ovary), with 77 identified cell types and approximately 450,000 open chromatin regions, encompassing candidate cis-regulatory elements such as promoters and distal regulatory regions, as well as accessible sites within intronic and exonic sequences. We integrated the publicly available mouse scRNA dataset with our snATAC dataset for each organ individually through cellular alignment, and observed a high degree of correspondence between cell types. This validates and enhances the reliability of the dataset for cell type annotation and allows further exploration of similarities and differences between mice and rats for cell type annotation in the same organ.

Furthermore, our findings emphasize two fundamental cellular relationships between different organs: cell type specificity (similarity across organs) and organ specificity (similarity within the same organ). The same major cell types (e.g., immune cells, endothelial cells, and stromal cells) display comparable chromatin accessibility profiles

across different organs, indicating that these cells share common functional attributes in diverse tissues. In contrast, organ specificity denotes the environmental adaptation and functional differentiation of cells within a specific organ (e.g., epithelial cells), resulting in heightened similarity between different cell types within the same organ.

2. Characterization of cell type- and organ-specific transcription factors (TFs)

Our dataset offers a valuable resource of the TF motifs in different organs, major cell types, and cell subtypes in the adult rat. We characterized numerous TFs as either shared or specific to individual organs. This information will facilitate the elucidation of gene regulatory networks in various organs and cell types and the identification of the potential roles of TFs in cell function and provide a crucial foundation for subsequent basic research and disease studies.

3. Shared and organ-specific features of endothelial and stromal cells

Cross-organ single-cell datasets enable systematic investigation of chromatin accessibility variation in the same cell type across different organs. In this study, endothelial and stromal cells were used as examples to illustrate how the same cell type can exhibit both shared and organ-specific molecular signatures across different organs. We found that both endothelial and stromal cells display organ-specific chromatin accessibility patterns near their typical marker genes, with most clusters dominated by cells from a single organ, while only a few clusters contained mixed-organ cells, indicating partial molecular conservation. Our analysis revealed that organ-specific differences in both gene scores and TF motif enrichments in endothelial and stromal cells largely correspond to the unique functional demands of each organ, with enriched genes and TFs aligning closely with organ-specific physiological roles and microenvironmental contexts. Together, these elements form a foundation for cross-organ cell type identification, annotation, and functional interpretation.

4. **Conservation and specificity of gene expression patterns among humans, mice, and rats revealed by cross-species analysis**

To further expand the applications of rat single-cell chromatin accessibility mapping, we attempted cross-omics and cross-species integration analyses to explore the conservation and species specificity of gene expression patterns in different organs. Our results demonstrate that gene scores can be utilized as reliable proxies for gene expression in cross-species data integration analyses, with the capacity to discern biologically salient features across species. We employed this method to investigate the degree of conservation of cell types and the extent of shared and distinct gene expression profiles in human, mouse, and rat hearts. The results provide a valuable foundation for elucidating cross-species differences and similarities in heart biology while offering insights into species-specific gene regulatory mechanisms.

In summary, we constructed a comprehensive single-nucleus multiple-organ chromatin accessibility map in the adult rat, which will serve as a valuable resource for investigating gene regulation and functional studies across organs, species, and disease models. Our work will attract significant attention and numerous citations in the field. Our team, including Yue Yuan, Chang Liu, and others, has extensive expertise in single-cell multi-omics research and epigenetics. Based on all these considerations, we hope you will consider our manuscript for publication in ***Gigascience.***

This manuscript has not been published or presented elsewhere in part or entirety and is not under consideration by another journal. All study participants provided informed consent, and the appropriate ethics review board approved the study design. We have read and understood your journal's policies and believe that neither the manuscript nor the study violates any of these.

Thank you for your consideration. I look forward to hearing from you.

Best regards,

Sincerely,

Yue Yuan on behalf of all authors

BGI-Research,

Hangzhou, China

袁 月

References:

1. Regev A, Teichmann SA, Lander ES, et al. The Human Cell Atlas. *Elife*. 2017;6. doi:10.7554/eLife.27041
2. Bakken TE, Jorstad NL, Hu Q, et al. Comparative cellular analysis of motor cortex in human, marmoset and mouse. *Nature*. 2021;598(7879):111-119. doi:10.1038/s41586-021-03465-8
3. Cusanovich DA, Hill AJ, Aghamirzaie D, et al. A Single-Cell Atlas of In Vivo Mammalian Chromatin Accessibility. *Cell*. 2018;174(5):1309-1324.e18. doi:10.1016/j.cell.2018.06.052
4. Zhang K, Hocker JD, Miller M, et al. A single-cell atlas of chromatin accessibility in the human genome. *Cell*. 2021;184(24):5985-6001.e19. doi:10.1016/j.cell.2021.10.024
5. Calderon D, Blecher-Gonen R, Huang X, et al. The continuum of Drosophila embryonic development at single-cell resolution. *Science*. 2022;377(6606):eabn5800. doi:10.1126/science.abn5800
6. Yu Y, Wei X, Deng Q, et al. Single-Nucleus Chromatin Accessibility Landscape Reveals Diversity in Regulatory Regions Across Distinct Adult Rat Cortex. *Front Mol Neurosci*. 2021;14:651355. doi:10.3389/fnmol.2021.651355
7. Ma P, Duan S, Ma W, et al. Single-cell chromatin accessibility landscape profiling reveals the diversity of epigenetic regulation in the rat nervous system. *Sci Data*. 2025;12(1):140. doi:10.1038/s41597-025-04432-y
