# Supplementary material for: Single-nucleus multiple-organ chromatin accessibility landscape in the adult rat
Source: Gigascience. 2026 Feb 3;15:giag013. doi: 10.1093/gigascience/giag013 (PMC12954174; doi:10.1093/gigascience/giag013)
Supplement: giag013_Reviewer_1_Report_original_submission — Reviewer 1 -- 9/15/2025 [file giag013_reviewer_1_report_original_submission.pdf]

Reviewer Report

Title: Single-nucleus multiple-organ chromatin accessibility landscape in the adult rat

Version: Original Submission **Date:** 9/15/2025

Reviewer name: Reviewer 1

Reviewer Comments to Author:

Li et al presents a manuscript where they generated a snATAC-seq atlas of 9 major organs in adult rat and integrated the atlas with mouse and human scRNA-seq and scATAC-seq data, revealing that chromatin accessbility is largely conserved between celltypes across species and that there also tissue-specific regulation in some celltypes even when they are common across several tissues. Overall, this looks like a great carefully analysed and annotated resource that would be useful for the community. I appreciate the amount of work that went into curating and analysing this dataset and i thought that the manuscript was very well written and clear.
I think the most interesting finding is in figure 3 where the authors found unique TFs regulating the same cell-types but in different organs. However, the analysis ends abruptly other than listing these TFs. Can the authors comment on what are the functional consequences/associations of these tissue-specific TFs, perhaps in the discussion?
The raw data is deposited into a database and can be openly downloaded but i find that the lack of processed data e.g. processed and labelled expression matrices or objects may prevent the adoption of this data by the community as it is a lengthy process to reach the author's conclusions. The authors might also want to consider incorporating an interactive platform for users to explore and navigate this dataset.
While i appreciate that the authors have detailed in their manuscripts how they performed the data analysis, i would still encourage the authors to upload their scripts/notebooks to an open code repository otherwise again it would be prohibitive for adoption by the community as it is.

Methods

Are the methods appropriate to the aims of the study, are they well described, and are necessary controls included? Choose an item.

Conclusions

Are the conclusions adequately supported by the data shown? Choose an item.

Reporting Standards

Does the manuscript adhere to the journal’s guidelines on minimum standards of reporting? Choose an item.

Choose an item.

Statistics

Are you able to assess all statistics in the manuscript, including the appropriateness of statistical tests used? Choose an item.

Quality of Written English

Please indicate the quality of language in the manuscript: Choose an item.

Declaration of Competing Interests

Please complete a declaration of competing interests, considering the following questions:

- Have you in the past five years received reimbursements, fees, funding, or salary from an organisation that may in any way gain or lose financially from the publication of this manuscript, either now or in the future?
- Do you hold any stocks or shares in an organisation that may in any way gain or lose financially from the publication of this manuscript, either now or in the future?
- Do you hold or are you currently applying for any patents relating to the content of the manuscript?
- Have you received reimbursements, fees, funding, or salary from an organization that holds or has applied for patents relating to the content of the manuscript?
- Do you have any other financial competing interests?
- Do you have any non-financial competing interests in relation to this paper?

If you can answer no to all of the above, write 'I declare that I have no competing interests' below. If your reply is yes to any, please give details below.

I declare that I have no competing interests

I agree to the open peer review policy of the journal. I understand that my name will be included on my report to the authors and, if the manuscript is accepted for publication, my named report including any attachments I upload will be posted on the website along with the authors' responses. I agree for my report to be made available under an Open Access Creative Commons CC-BY license (http://creativecommons.org/licenses/by/4.0/). I understand that any comments which I do not wish to be included in my named report can be included as confidential comments to the editors, which will not be published.

Choose an item.

To further support our reviewers, we have joined with Publons, where you can gain additional credit to further highlight your hard work (see: https://publons.com/journal/530/gigascience). On publication of this paper, your review will be automatically added to Publons, you can then choose whether or not to claim your Publons credit. I understand this statement.

Yes Choose an item.
