# Supplementary material for: Single-nucleus multiple-organ chromatin accessibility landscape in the adult rat
Source: Gigascience. 2026 Feb 3;15:giag013. doi: 10.1093/gigascience/giag013 (PMC12954174; doi:10.1093/gigascience/giag013)
Supplement: giag013_Reviewer_2_Report_original_submission — Reviewer 2 -- 9/23/2025 [file giag013_reviewer_2_report_original_submission.pdf]

Reviewer Report

Title: Single-nucleus multiple-organ chromatin accessibility landscape in the adult rat

Version: Original Submission **Date:** 9/23/2025

Reviewer name: Reviewer 2

Reviewer Comments to Author:

In this manuscript, Li et al. present a single-nucleus rat atlas of chromatin accessibility, with over 110,000 cells measured. They annotate cell types, compare types across organs, and identify organ-specific or shared features, inferring transcription factors and gene regulatory programs. They also integrate human and mouse tissue to validate many of these findings. I found the manuscript to be of value for many biologists due to the addition of an exhaustive effort to characterize cells in the rat, however the actual analyses and findings are not entirely novel. However, that is not the overall goal of the manuscript, so my enthusiasm remains high. Specific comments are as follows:

- The introduction is quite short and relatively shallow, with very little justification for the use of a rat atlas. The authors should elaborate on this justification and provide a more comprehensive overview of the use of these atlases in greater detail, rather than just providing a list of undefined analyses which may not be familiar to the reader.
- The authors extracted tissues from a single rat. Although it is beneficial to have a deep characterization of an individual, such a small sample size would not be the best representation for an entire atlas (this is mentioned in the limitations).
- The authors could consider integrating other rat snATAC-seq data sets, of which several exist.
- The TSS enrichment scores and number of fragments are negatively correlated, why is that?
- How reliable are the identified doublets? If the authors run the algorithm to detect doublets on individual samples or replicates versus all samples, are the same cells identified?
- Figure 1 has icons and images which are quite small.
- The authors mention LSI dimensionality reduction for clustering, but then display everything with UMAP which is known to distort distances.
- UMAP is also notoriously bad at handling batch effects, which seem to be present in this atlas. Has any effort been used to mitigate this issue? It would be better to use an alternative visualization that maintains distances.
- How reliable are the cell types from the automated annotator given the relationships in the hierarchical clustering? For example, "Immature_T_cell"s are more closely clustered with "Goblet" cells than "Thymic_T_Cell"s, and these types of relationships are throughout the tree. Additional validation should be completed, including at minimum a gene expression scoring of markers for each of these cell types to see if they match the annotation.
- To determine the organ specificity of different cell types, the authors can additionally look at, for instance, alveolar / interstitial macrophages in the lung or another tissue or tissue resident fibroblasts, comparing those specific cell types rather than alveolar vs. any other epithelial cell type.
- The finding that endothelial clusters were mostly grouped by organs should be verified in the context

of batch correction. The authors could also consider clustering befor dimensionality reduction and using consensus clustering using subsampling.
- l. 369: CMs not defined.
- l. 396: ECs not defined.
- "sc" and "sn" used interchangeably (e.g. Figure 5A), which should be "snATAC-seq".
- l. 445: The central dogma of molecular biology defined here is stated incorrectly and should be fixed or removed, although it appears irrelevant for the discussion.

Methods

Are the methods appropriate to the aims of the study, are they well described, and are necessary controls included? Choose an item.

Conclusions

Are the conclusions adequately supported by the data shown? Choose an item.

Reporting Standards

Does the manuscript adhere to the journal's guidelines on minimum standards of reporting? Choose an item.

Choose an item.

Statistics

Are you able to assess all statistics in the manuscript, including the appropriateness of statistical tests used? Choose an item.

Quality of Written English

Please indicate the quality of language in the manuscript: Choose an item.

Declaration of Competing Interests

Please complete a declaration of competing interests, considering the following questions:

- Have you in the past five years received reimbursements, fees, funding, or salary from an organisation that may in any way gain or lose financially from the publication of this manuscript, either now or in the future?
- Do you hold any stocks or shares in an organisation that may in any way gain or lose financially from the publication of this manuscript, either now or in the future?
- Do you hold or are you currently applying for any patents relating to the content of the manuscript?

- Have you received reimbursements, fees, funding, or salary from an organization that holds or has applied for patents relating to the content of the manuscript?
- Do you have any other financial competing interests?
- Do you have any non-financial competing interests in relation to this paper?

If you can answer no to all of the above, write 'I declare that I have no competing interests' below. If your reply is yes to any, please give details below.

I declare that I have no competing interests

I agree to the open peer review policy of the journal. I understand that my name will be included on my report to the authors and, if the manuscript is accepted for publication, my named report including any attachments I upload will be posted on the website along with the authors' responses. I agree for my report to be made available under an Open Access Creative Commons CC-BY license (http://creativecommons.org/licenses/by/4.0/). I understand that any comments which I do not wish to be included in my named report can be included as confidential comments to the editors, which will not be published.

Choose an item.

To further support our reviewers, we have joined with Publons, where you can gain additional credit to further highlight your hard work (see: https://publons.com/journal/530/gigascience). On publication of this paper, your review will be automatically added to Publons, you can then choose whether or not to claim your Publons credit. I understand this statement.

Yes Choose an item.
