# Supplementary material for: Single-nucleus multiple-organ chromatin accessibility landscape in the adult rat
Source: Gigascience. 2026 Feb 3;15:giag013. doi: 10.1093/gigascience/giag013 (PMC12954174; doi:10.1093/gigascience/giag013)
Supplement: giag013_Reviewer_3_Report_original_submission — Reviewer 3 -- 9/25/2025 [file giag013_reviewer_3_report_original_submission.pdf]

Reviewer Report

Title: Single-nucleus multiple-organ chromatin accessibility landscape in the adult rat

Version: Original Submission **Date:** 9/25/2025

Reviewer name: Reviewer 3

Reviewer Comments to Author:

In this study, Ronghai Li and colleagues constructed an extensive multi-organ single-nucleus chromatin accessibility atlas of the model organism Rattus norvegicus. The authors generated a comprehensive dataset encompassing 115,723 single-nucleus chromatin accessibility profiles across nine organs (thyroid, thymus, heart, lung, liver, spleen, kidney, pancreas, and ovary). Each organ was profiled in duplicate or triplicate, thereby ensuring reproducibility and robustness of the dataset. The authors also performed rigorous preprocessing and filtering steps, which provided a high-quality foundation for downstream analyses.

The downstream analyses were multifaceted and thoughtfully executed in the following steps: 1) Low-dimensional visualization of all cells within the atlas, represented by organs, which led to the identification of six major cell types; 2) A census of the distribution of major cell types across the analyzed organs; 3) Integration with existing mouse single-cell RNA-seq atlases to refine cell type annotations (77 cell subtypes) and ensure cross-species comparability; 4) Inference of transcription factor activities from open chromatin profiles, providing important insights into gene regulatory mechanisms.

Building upon these analyses, the authors focused on shared and organ-specific features of endothelial and stromal cells, thereby highlighting both conserved and divergent regulatory programs. Finally, through integration of human, rat, and mouse scRNA- and scATAC-seq atlases for the heart and kidney, they investigated cross-species similarities and differences in gene expression and regulatory patterns, further strengthening the relevance and translational potential of this resource.

In my opinion, the manuscript by Ronghai Li et al. is well written, the data are of very high quality, and the study represents a significant data resource. The rat (Rattus norvegicus) is a widely used and indispensable model organism in biomedical research, particularly for studies related to disease onset, progression, and therapeutic development. The generation of this single-nucleus chromatin accessibility atlas, together with the comprehensive analyses provided, constitutes a valuable resource for dissecting organ- and tissue-specific regulatory landscapes. This work not only enhances our understanding of gene regulation across organs but also facilitates cross-species comparisons that will be of great importance for translational research. I therefore strongly support acceptance of the manuscript by Ronghai Li et al. for publication in GigaScience., contingent only upon minor revisions as outlined below:

Minor revisions:

1) While the data preprocessing and analysis steps are clearly described in the Methods section, the code used for data analysis is currently available only upon request. I believe that future readers and, in particular, potential users of this valuable resource would greatly benefit if the analysis code were made publicly accessible. Open availability of the code would not only enhance transparency and

reproducibility but also facilitate broader adoption of the dataset. This is especially important as new single-cell ATAC-seq and RNA-seq datasets become available, since ready access to the analysis pipeline will accelerate and streamline future studies that build upon the provided atlas.
2) Line 131: Stromal cells, immune cells, and endothelial cells from different organs tend to be clustered together (i.e., by cel type) rather than clustered according to the organ of origin or sample batch (Figure 1E).
and
Line 137: However, these cells tended to cluster by organ rather than by cell type (Figure 1E).
This observation can be clearly seen in the UMAPs (Figure 1C-D), but not in the census plot (Figure 1E). I therefore recommend revising the figure reference to (Figure 1C-D) to improve accuracy and clarity for the reader.
3) Authors often assess, across manuscript, whether cells cluster in a cell type-specific or organ-specific manner. For immune and epithelial cells, however, the conclusions can be confounded, as they vary depending on the analytical method used (e.g., scATAC-seq alone versus integration with scRNA-seq data). Could the authors elaborate on the robustness of these observations with respect to the choice of UMAP parameters, particularly the number of features included and the dimensionality of the LSI applied?
4) The authors used the CIS-BP database of transcription factor motifs to assess cell type-specific transcription factor activities. However, JASPAR, which is a curated database, is more commonly used in scATAC-seq studies. Could the authors clarify the rationale for choosing CIS-BP over JASPAR?

Methods

Are the methods appropriate to the aims of the study, are they well described, and are necessary controls included? Choose an item.

Conclusions

Are the conclusions adequately supported by the data shown? Choose an item.

Reporting Standards

Does the manuscript adhere to the journal’s guidelines on [minimum standards of reporting?]() Choose an item.

Choose an item.

Statistics

Are you able to assess all statistics in the manuscript, including the appropriateness of statistical tests used? Choose an item.

Quality of Written English

Please indicate the quality of language in the manuscript: Choose an item.

Declaration of Competing Interests

Please complete a declaration of competing interests, considering the following questions:

- Have you in the past five years received reimbursements, fees, funding, or salary from an organisation that may in any way gain or lose financially from the publication of this manuscript, either now or in the future?
- Do you hold any stocks or shares in an organisation that may in any way gain or lose financially from the publication of this manuscript, either now or in the future?
- Do you hold or are you currently applying for any patents relating to the content of the manuscript?
- Have you received reimbursements, fees, funding, or salary from an organization that holds or has applied for patents relating to the content of the manuscript?
- Do you have any other financial competing interests?
- Do you have any non-financial competing interests in relation to this paper?

If you can answer no to all of the above, write 'I declare that I have no competing interests' below. If your reply is yes to any, please give details below.

I declare that I have no competing interests.

I agree to the open peer review policy of the journal. I understand that my name will be included on my report to the authors and, if the manuscript is accepted for publication, my named report including any attachments I upload will be posted on the website along with the authors' responses. I agree for my report to be made available under an Open Access Creative Commons CC-BY license (http://creativecommons.org/licenses/by/4.0/). I understand that any comments which I do not wish to be included in my named report can be included as confidential comments to the editors, which will not be published.

Choose an item.

To further support our reviewers, we have joined with Publons, where you can gain additional credit to further highlight your hard work (see: https://publons.com/journal/530/gigascience). On publication of this paper, your review will be automatically added to Publons, you can then choose whether or not to claim your Publons credit. I understand this statement.

Yes Choose an item.
